# The endocrine product of renal (preglomerular) contractile pericytes depends on prolyl‐4‐hydroxylases 2 and 3

**DOI:** 10.1113/JP290695

**Published:** 2026-05-04

**Authors:** Bettina K. M. Firmke, Lena M. Süß, Anna‐Lena Forst, Armin Kurtz, Katharina A.‐E. Broeker

**Affiliations:** ^1^ Physiology I; Institute of Physiology University of Regensburg Regensburg Germany; ^2^ Medical Cell Biology, Institute of Physiology University of Regensburg Regensburg Germany

**Keywords:** endocrine plasticity, erythropoietin, HIF‐2α stabilization, preglomerular vascular smooth muscle cells, prolyl‐4‐hydroxylases, renal contractile pericytes, renin

## Abstract

**Abstract:**

Renal juxtaglomerular renin‐producing cells and preglomerular vascular smooth muscle cells (VSMCs) are specialized pericytes with notable plasticity. Preglomerular VSMCs can convert to renin‐producing cells during severe hypotension or salt depletion, and renin cells can transform into erythropoietin (EPO)‐producing cells when hypoxia‑inducible factor (HIF)‐2α is stabilized through deletion of prolyl‐4‐hydroxylases (PHD) 2 and 3. These findings raise the question of whether PHD2 and PHD3 likewise regulate the endocrine plasticity of preglomerular VSMCs. To investigate the role of PHD2 and/or PHD3 in (preglomerular) contractile pericytes, inducible mouse models with smooth muscle myosin heavy chain (SMMHC)‐specific deletion of PHD2 and/or PHD3 were examined under basal conditions or after stimulation of renin production by treating the mice with a low‐salt diet and angiotensin converting enzyme inhibitor enalapril (LSE). At baseline, none of the deletions altered renin production or induced EPO expression in preglomerular pericyte‐like VSMCs, despite HIF‐2α stabilization in PHD2/PHD3‐deficient mice. However, HIF‐2α stabilization resulting from PHD2 or PHD2/PHD3 deletion triggered EPO production in interstitial SMMHC^+^ contractile pericytes. LSE treatment induced renin in VSMCs and extraglomerular mesangial cells of control, SMMHC^CreERT2^ PHD2^ff^ and SMMHC^CreERT2^ PHD3^ff^ mice. In contrast, VSMCs of PHD2/PHD3‐deficient mice produced EPO rather than renin, while renin induction persisted only in mesangial cells. Notably, this LSE‐induced EPO production was reversible despite ongoing HIF‐2α stabilization. Transcriptional changes indicated a shift in PHD2/PHD3‐deficient VSMCs from a contractile/renin cell‐like to a contractile/EPO cell‐like signature. These findings indicate that HIF‐2α stabilization determines the endocrine product of preglomerular VSMCs and interstitial pericytes. Notably, loss of PHD2/PHD3 does not compromise the plasticity of VSMCs to reversibly adopt endocrine functions.

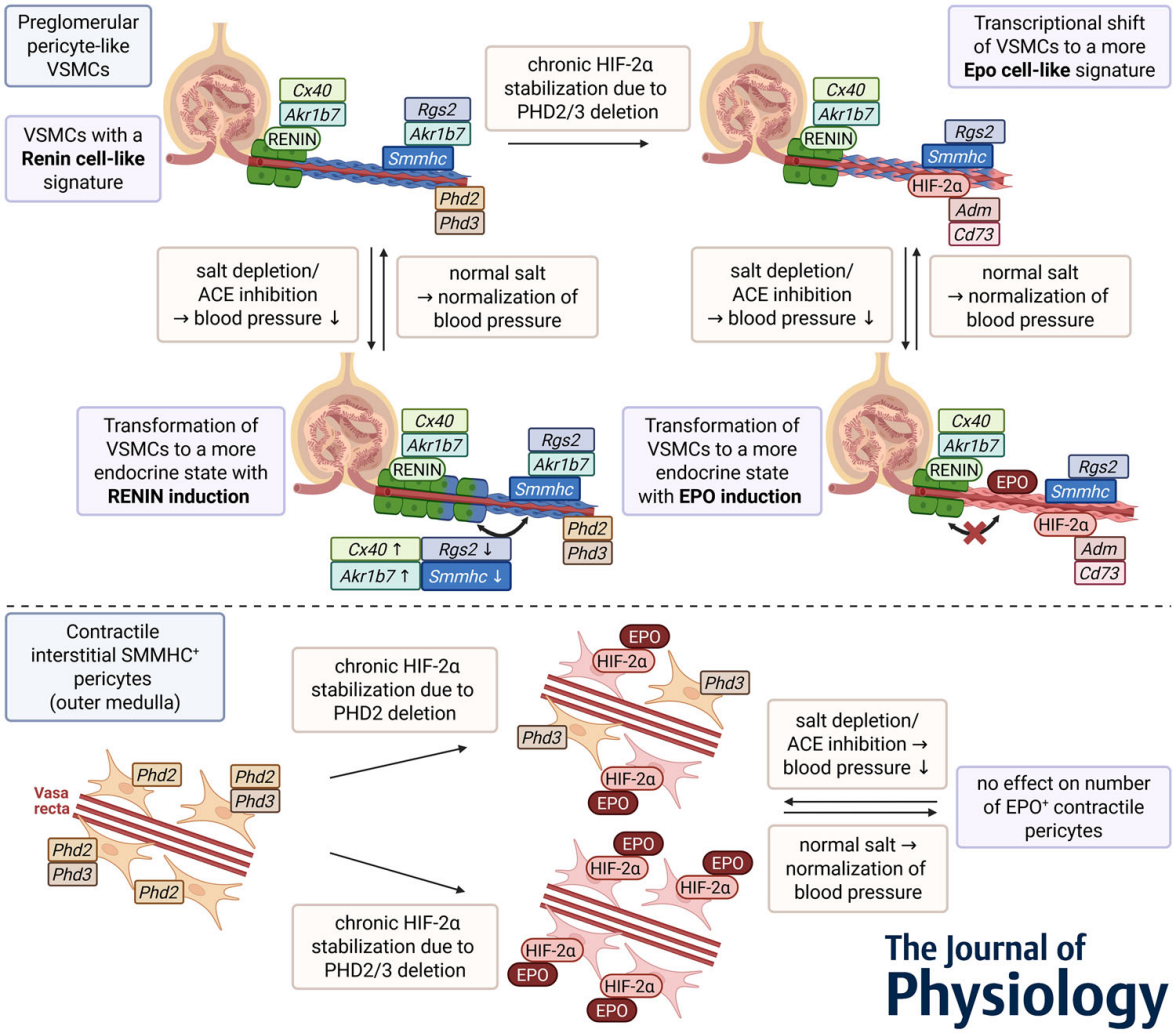

**Key points:**

- Smooth muscle myosin heavy chain (SMMHC)‐specific deletion of the prolyl‑4‑hydroxylases PHD2 and PHD3 stabilized hypoxia‑inducible factor (HIF)‑2α in preglomerular pericyte‑like vascular smooth muscle cells (VSMCs), prompting a transcriptional shift from a contractile/renin cell‑like toward a more contractile/EPO cell‑like signature without activating erythropoietin (EPO) transcription.
- A reduction in systolic blood pressure through treatment with low‐salt diet and angiotensin converting enzyme inhibitor enalapril induced EPO synthesis instead of renin in preglomerular PHD2/PHD3‐deficient VSMCs. Transformation of preglomerular VSMCs into EPO‐producing cells was reversible despite persistent HIF‐2α stabilization.
- SMMHC cell‐specific deletion of PHD2 and PHD2/PHD3 activated EPO production in interstitial contractile pericytes independent of systolic blood pressure.
- Short‑term HIF‑2α stabilization was insufficient to induce EPO production in preglomerular VSMCs or contractile pericytes.
- Τaken together these findings demonstrate that HIF‐2α stabilization governs the endocrine output of preglomerular VSMCs and interstitial pericytes. Notably, the loss of PHD2/PHD3 does not impair the capacity of VSMCs to reversibly assume endocrine functions.

## Graphical abstract

**Abstract figure legend** Preglomerular pericyte‐like SMMHC^+^ vascular smooth muscle cells (VSMCs) display marked endocrine plasticity. Hypotension caused by salt depletion and angiotensin‐converting enzyme (ACE) inhibition induces a reversible transformation of these VSMCs toward an endocrine state that produces renin. Chronic HIF‐2α stabilization – by SMMHC‐specific PHD2/PHD3 deletion – reprograms the VSMCs from a contractile/renin cell‐like toward a more contractile/erythropoietin (EPO) cell‐like transcriptional signature, yet without inducing EPO synthesis. Under these conditions, hypotension triggers a reversible switch to an endocrine state that produces EPO instead of renin. In the closely related SMMHC^+^ interstitial contractile pericytes, deletion of PHD2 or combined PHD2/PHD3 loss induces HIF‐2α‐dependent EPO production in the subset of solely PHD2^+^ pericytes or in both PHD2^+^ and PHD2/3^+^ pericytes, respectively, independent of systolic blood pressure. Overall, PHD2 and PHD3 determine the endocrine output of preglomerular VSMCs and interstitial pericytes by regulating HIF‐2α, while their loss does not impair the plasticity of VSMCs to reversibly adopt endocrine functions. Created with BioRender.com.

## Introduction

The kidneys fulfil several endocrine functions like the production of renin and erythropoietin (EPO). Renin is primarily produced by the juxtaglomerular cells, specialized myoendocrine pericyte‐like cells of the afferent arterioles (Stefanska et al., 2016). As a central component of the renin–angiotensin–aldosterone system, renin plays a pivotal role in regulating blood pressure, extracellular volume and electrolyte balance. EPO, on the other hand, is primarily produced by interstitial platelet‐derived growth factor receptor‐β (PDGFR‐β) positive fibroblasts/pericytes located along the corticomedullary border (Bachmann et al., 1993; Gerl et al., 2016; Maxwell et al., 1993). Despite EPO and renin operating within different regulatory systems, the regulation of EPO and renin production shares notable similarities. An increased demand for either protein is met by transforming additional cells into renin‐ or EPO‐producing cells rather than enhancing production per cell. In the case of renin, a subset of pericyte‐like vascular smooth muscle cells (VSMCs) along the afferent arterioles and interlobular arteries revert to a renin‑producing phenotype (Sequeira López et al., 2004). These preglomerular VSMCs derive from the renin cell lineage during kidney development and therefore retain characteristics of renin cells, pericytes and smooth muscle cells (Brunskill et al., 2011; Stefanska et al., 2016; Yamaguchi et al., 2023). Extraglomerular mesangial cells can also undergo transformation into renin‑producing cells (Sequeira López et al., 2004). During this reversible transformation, which is triggered, for example, by intrarenal hypotension or salt deficiency, pericyte‐like preglomerular VSMCs lose contractile properties and adopt a more endocrine phenotype (Brunskill et al., 2011).

Increased EPO production involves the induction of *Epo* transcription in additional resident interstitial PDGFR‐β^+^ fibroblasts/pericytes located within the cortex and the outer stripe of the outer medulla. Depending on the strength of the EPO‑inducing stimulus, fibroblasts in the deep cortex and the outer stripe of the outer medulla are activated first. With stronger stimuli, *Epo* expression is also induced in interstitial fibroblasts that are closer to the cortical surface (Bachmann et al., 1993; Eckardt et al., 1993). Physiologically, EPO production is stimulated by hypoxaemic conditions. At the cellular level, *Epo* gene regulation is controlled by hypoxia‑inducible factor (HIF)‐2α, a heterodimeric transcription factor composed of the HIF‑2α and HIF‑1β subunits (Haase, 2013; Paliege et al., 2010). Oxygen sensing is mediated by prolyl‑4‑hydroxylases (PHD) 1–3, among which PHD2 and PHD3 are the main isoforms involved in renal EPO regulation (Kobayashi et al., 2016; Minamishima et al., 2008). Under normoxic conditions, the HIF‑2α subunit is hydroxylated by PHD2 and PHD3 in an oxygen‑dependent manner. Hydroxylated HIF‑2α is subsequently recognized by the von Hippel–Lindau (VHL) protein, an E3 ubiquitin ligase, which ubiquitinates HIF‑2α and marks it for proteasomal degradation. Under hypoxaemic conditions, reduced tissue oxygen availability prevents PHD‐mediated hydroxylation of HIF‑2α, thereby allowing the protein to evade proteasomal degradation. Instead, stabilized HIF‑2α translocates into the nucleus, where it dimerizes with HIF‑1β and, together with additional transcriptional cofactors, drives the expression of *Epo* and other HIF‑2α‐dependent target genes (Dahl, Bapst et al., 2022; Koivunen & Kietzmann, 2018).

Juxtaglomerular renin cells, preglomerular pericyte‐like VSMCs and (EPO‐producing) interstitial fibroblasts originate from a common progenitor population of FoxD1‐positive stromal cells that also give rise to intra‐ and extraglomerular mesangial cells (Gerl et al., 2017; Humphreys et al., 2010; Sequeira Lopez et al., 2001). Furthermore, we and others previously demonstrated that renin and EPO cells are even more closely related than previously thought: in adult kidneys, a subset of interstitial fibroblasts expresses renin and following anaemia or pharmacological PHD inhibition, some interstitial fibroblasts even co‐express EPO and renin (Broeker et al., 2021; Miyauchi et al., 2021). Notably, juxtaglomerular renin cells can also undergo metaplastic transformation into EPO‐producing cells not only during kidney development but also in adulthood (Gerl et al., 2015; Kurt et al., 2015). This transformation is driven by the loss of PHD2 and PHD3, key regulators of hypoxia signalling and EPO expression. Their deletion leads to chronic activation of the hypoxia pathway, resulting in HIF‐2α‐dependent downregulation of renin and induction of EPO production. During this process, juxtaglomerular cells stop the expression of characteristic renin cell markers such as *Akr1b7* and *Cx40*, while initiating the expression of EPO‐associated markers including *Nt5e* (CD73) and *Pdgfrb* (Broeker et al., 2021).

Given that preglomerular pericyte‐like VSMCs can revert to a renin‐producing phenotype and share the same developmental origin as renin cells and interstitial PDGFR‐β^+^ (EPO)‐producing fibroblasts, we investigated the significance of the PHD isoforms PHD2 and PHD3 for the endocrine function and plasticity of VSMCs. Do they influence the endocrine product of VSMCs? Do they affect the reversible transformation of preglomerular VSMCs in response to a renin‐inducing stimulus? To address these questions, we examined changes in renal renin and EPO production in mouse models with tamoxifen‐inducible smooth muscle myosin heavy chain (SMMHC)‐specific deletion of PHD2, PHD3, or both PHD2 and PHD3 under basal conditions. Furthermore, the different PHD‐deficient mouse models were fed a low‐salt diet combined with the angiotensin‐converting enzyme (ACE) inhibitor enalapril, as this treatment is well established for stimulating renin production in VSMCs and extraglomerular mesangial cells by lowering systolic blood pressure (Guessoum et al., 2020; Neubauer et al., 2013; Pentz et al., 2012). Additionally, we employed targeted spatial analysis to evaluate the expression of characteristic cell markers of juxtaglomerular renin cells, VSMCs and EPO‐producing cells.

## Methods

### Ethical approval

Approval for all animal experiments was granted by the local ethics committee (Regierung von Unterfranken; RUF‐55.2.2‐2532‐2‐1754; RUF‐55.2.2‐2532‐2‐2173). All experiments comply with the Directive 2010/63/EU of the European Parliament and of the Council on the protection of animals used for scientific purposes. They were also conducted in accordance with the guidelines of the University of Regensburg's animal research centre and comply with the animal ethics checklist of this journal (Grundy, 2015).

### Animals

All animals were bred and kept in the animal facility at the University of Regensburg. The following controlled housing conditions apply there: a 12‐h light–dark cycle, controlled temperature levels (22 ± 2°C) and humidity (55 ± 10%). The animals had free access to autoclaved tap water and were fed a standard rodent chow (normal salt diet, NS, 0.6% NaCl; Ssniff Spezialdiäten GmbH, Soest, Germany).

To analyse the significance of PHD2 and/or PHD3 for SMMHC^+^ cells, mice expressing a tamoxifen‐inducible Cre recombinase under the SMMHC promotor (SMMHC^CreERT2^; JAX stock #19079) (Wirth et al., 2008) were bred with mice carrying loxP‐flanked PHD2 alleles (Franke et al., 2013; Singh et al., 2013) and/or PHD3 alleles (Takeda et al., 2006). The resulting mouse models, SMMHC^CreERT2^ PHD2^ff^, SMMHC^CreERT2^ PHD3^ff^ and SMMHC^CreERT2^ PHD2^ff^ PHD3^ff^, are also referred to in the text as PHD2‐KO, PHD3‐KO and PHD2/PHD3‐KO, respectively. The primers used for genotyping are listed in Table 1. As SMMHC^CreERT2^ is located on the Y chromosome, only male mice could be used in the experiments. Mice that were negative for SMMHC^CreERT2^ were used as controls.

**Table 1:** Genotyping primer sequences

Genotype	Sequence (5′ to 3′), fwd	Sequence (5′ to 3′), rev	Sequence (5′ to 3′), rev2
SMMHC^CreERT2^	TGACCCCATCTCTTCACTCC	AGTCCCTCACATCCTCAGGTT	
PHD2 flox	CGCATCTTCCATCTCCATTT	CTCACTGACCTACGCCGTGT	GGCAGTGATAACAGGTGCAA
PHD3 flox	ATGGCCGCTGTATCACCTGTAT	CCACGTTAACTCTAGAGCCACTGA	

SMMHC^CreERT2^ activity was induced in the mice at 8–12 weeks of age by feeding them a tamoxifen‐containing diet (400 mg tamoxifen citrate per kilogram; A115T00404; Ssniff) for 21 days. The mice were then kept on the standard rodent diet (normal salt diet, NS) for a further 21 days.

To stimulate renin production, the mice were fed a low‐salt diet (<0.03% Na^+^, e.g. Ssniff) and treated with the ACE inhibitor enalapril for 14 days (low‐salt/enalapril treatment, LSE, treatment). The mice received enalapril as enalapril maleate in their drinking water (50 mg/l; solution of Enadog tablets in drinking water; Dechra Veterinary Products Deutschland GmbH, Aulendorf, Germany).

To stabilize HIF‐2α continuously for 12 h, wild‐type mice that had been fed an NS or LSE diet for 14 days were given eight doses of the PHD inhibitor roxadustat at 90‐min intervals. The roxadustat solution (50 mg/kg; solution of Evrenzo^TM^ tablets in 0.5 m Tris–HCl buffer (pH 9.0) and raspberry syrup; Astellas Pharma, Munich, Germany) was administered orally using the micropipette‐guided drug application method. Similar numbers of male and female mice (aged 12–24 weeks, C57/Bl6J genetic background) were included in the experimental groups.

Systolic blood pressure in conscious control and SMMHC^CreERT2^ PHD2^ff^ PHD3^ff^ mice was measured using a tail‐cuff manometry system (TSE Systems, Berlin, Germany). Prior to data collection, mice underwent a 7‐day acclimation period: during the first 2 days, they were placed in the restraining device for 15 min without measurements, followed by 5 days of measurement training. For analysis under NS or LSE treatment conditions, blood pressure values obtained on days 10–14 of the respective treatment period were used (= five consecutive days at the end of the treatment phase). Each day, 10 measurement cycles were performed per animal and averaged. Systolic blood pressure under NS and LSE was assessed in the same mice across consecutive NS and LSE cycles. Control and PHD2/PHD3‐KO mice were sex and age matched.

To obtain plasma for plasma renin, plasma EPO and plasma ACE measurements, blood was collected from the facial vein or abdominal artery (during organ removal) in ammonium‐heparin‐coated capillaries (KABE Labortechnik GmbH, Nümbrecht, Germany). The blood samples were centrifuged for 4 min at 13684 g at room temperature. Haematocrit values were then determined, and the plasma samples were stored at −80°C prior to use.

For kidney removal, the mice were anaesthetized with ketamine/xylazine (ketamine, 100 mg/kg body weight; xylazine 10 mg/kg body weight, intraperitoneal) and the animals died by exsanguination (blood collection). The left kidneys were removed and flash‐frozen in liquid nitrogen for RNA isolation and the right kidneys were perfusion‐fixed for tissue analysis.

### Determination of plasma renin, erythropoietin and ACE concentrations

Plasma renin and erythropoietin concentrations were determined using the Invitrogen Mouse Renin 1 ELISA Kit (Thermo Fisher Scientific Inc., Waltham, MA, USA) and the Mouse Erythropoietin/EPO Quantikine ELISA Kit (Bio‐Techne, Minneapolis, MN, USA), respectively, following the manufacturers' protocols. Plasma ACE concentrations were quantified using the Mouse ACE ELISA Kit (Antibodies.com Europe AB, Stockholm, Sweden) in accordance with the manufacturer's instructions.

### Determination of renal mRNA expression levels

RNA was isolated from the left kidney according to the protocol described by Chomczynski & Sacchi (1987). Subsequently, 1 µg of RNA was transcribed into cDNA using oligo‐dTs and Moloney murine leukaemia reverse transcriptase (Thermo Fisher Scientific). Renal mRNA abundances were determined by real‐time qPCR using the LightCycler 96 instrument and the SYBR Green I Master Kit (Roche Diagnostics, Mannheim, Germany). Ribosomal protein L32 (*Rpl32*) was used as the housekeeping gene for normalization. The primer sequences (Eurofins, Munich, Germany) are listed in Table 2.

**Table 2:** RT‐qPCR primer

Genes	Sequence (5′ to 3′), fwd	Sequence (5′ to 3′), rev	Product size (bp)
*Rpl32*	TTAAGCGAAACTGGCGGAAAC	TTGTTGCTCCCATAACCGATG	100
*Epo*	AATGGAGGTGGAAGAACAGG	ACCCGAAGCAGTGAAGTGA	174
*Renin*	ATGAAGGGGGTGTCTGTGGGGTC	ATGTCGGGGAGGGTGGGCACCTG	194

Abbreviations: *Rpl32*, ribosomal protein L32; *Epo*, erythropoietin

### *In situ* hybridization using RNAscope technology

*In situ* hybridization was performed using the RNAscope Multiplex Fluorescent v2 Kit (Bio‐Techne), following the manufacturer's protocol (Wang et al., 2012). To prepare the tissue, the kidneys were perfused with 30 ml of sterile phosphate‐buffered saline (PBS pH 7.4), followed by 40 ml of a 10% neutral buffered formalin solution (NBF, pH 7.0). The kidneys were then post‐fixed for 30–36 h in 10% NBF under gentle agitation. Following dehydration using an ascending ethanol–isopropanol series, the kidneys were embedded in paraffin. Kidney sections 5 µm thick were used for the assay. Fluorophores TSA Vivid 570 and 650 (Bio‐Techne), as well as Opal 780 (Akoya Biosciences, Marlborough, MA, USA), were used to detect the target mRNAs. Nuclei were counterstained with 4′,6‐diamidino‐2‐phenylindole (DAPI). To ensure long‐term signal stability, the sections were covered with ProLong Gold Antifade Mounting Medium (Thermo Fisher Scientific) and stored in the dark at 4°C. Table 3 lists the RNAscope probes used in this study.

**Table 3:** RNAscope probes

RNAscope probe	Cat. no.	RNAscope probe	Cat. no.
Mm‐*Adm*	493601	Mm‐*Pdgfrb*	411381
Mm‐*Akr1b7*‐O1	589271	Mm‐*Pdgfrb*‐C3	411381‐C3
Mm‐*Crebbp*‐C2	1129261‐C2	Mm‐*Renin*‐C3	433461‐C3
Mm‐*Egln1* (PHD2)	315491	Mm‐*Rgs2*‐C2	492931‐C2
Mm‐*Egln3* (PHD3)	434931	Mm‐*Rgs4*	467461
Mm‐*Epo*	315501	Mm‐*Rgs4*‐C3	467461‐C3
Mm‐*Epo*‐C2	315501‐C2	Mm‐*Rgs5*	430181
Mm‐*Gja5* (Cx40)	518041	Negative control probe	320751
Mm‐*Gjc1* (Cx45)	538911	Positive control probe	321651
Mm‐*Myh11* (Smmhc)	316101		

Abbreviations: *Adm*, adrenomedullin; *Akr1b7*, aldo‐keto‐reductase family 1, member B7; *Creb‐bp*, CREB‐binding protein; *Egln1/3*, Egl nine homolog 1/3 (PHD2/PHD3, prolyl‐4‐hydroxylases 2/3); *Epo*, erythropoietin; *Gja5*, gap junction protein alpha 5 (*Cx40*, connexin 40); *Gjc1*, gap junction protein gamma 1 (*Cx45*, connexin 45); *Myh11*, myosin heavy chain 11 (*Smmhc*; smooth muscle myosin heavy chain), *Pdgfrb*, platelet‐derived growth factor receptor beta; *Rgs2/4/5*, regulator of G‐protein signalling 2/4/5

For detection of *Epo* mRNA in SMMHC^+^ interstitial pericytes, RNAscope was combined with immunofluorescence using the RNA‐Protein Co‐Detection Ancillary kit (Bio‐Techne). Kidney sections (5 µm) from NBF‑fixed, paraffin‑embedded kidneys were deparaffinized and pretreated according to the manufacturer's protocol. The sections were then incubated overnight at 4 °C with a SMMHC antibody (rabbit polyclonal to smooth muscle myosin heavy chain 11, dilution 1:100; ab53219, Abcam Inc., Waltham, MA, USA) diluted in Co‑Detection Antibody Diluent. The next day, the sections were washed in 1× PBS with 0.1% Tween‑20 (PBS‑T) and post‑fixed for 30 min in 10% NBF. After another wash in PBS‑T, *Epo* mRNA was hybridized and detected using the RNAscope assay. Subsequently, the sections were incubated for 30 min at room temperature with donkey anti‑rabbit horseradish peroxidase (HRP)‑linked antibody (dilution 1:200; CS7074; Cell Signaling Technology, Danvers, MA, USA), washed twice with PBS‑T, and then incubated for 10 min with the fluorophore TSA Vivid 650. Following counterstaining with DAPI, the sections were mounted with ProLong Gold Antifade Mounting Medium.

### Determination of EPO protein concentration in tissue homogenates

To quantify renal EPO protein levels, mice were euthanized by cervical dislocation and immediately perfused with ice‑cold sterile PBS to remove blood from the kidneys. Kidneys were excised and flash‑frozen in liquid nitrogen. For total protein extraction, kidneys were weighed and lysed in PBS at a 1:5 (w/v) ratio by sonication. Tissue lysates were kept on ice for 1 h, centrifuged at 8000 *g* for 30 min at 4°C, aliquoted, and stored at −80°C. Kidney lysates were assayed without prior dilution using the Mouse Erythropoietin/EPO Quantikine ELISA Kit (Bio‑Techne) according to the manufacturer's instructions. Kidney EPO concentration was expressed as the amount of EPO detected normalized to the total protein content of each sample (pg/mg). Total protein concentration was determined using the Pierce BCA Protein Assay Kit (Thermo Fisher Scientific) according to the manufacturer's instructions.

### Detection of HIF‐2α stabilization

Immunohistochemical staining to detect HIF‐2α stabilization was performed as previously described (Firmke et al., 2025). In brief, the kidneys were perfused with 3% paraformaldehyde solution, immediately dehydrated using an ascending methanol–isopropanol series and embedded in paraffin. To permeabilize the tissue, 5 µm‐thick sections were boiled in Target Retrieval Solution (Agilent Technologies, Waldbronn, Germany). The sections were then blocked with an avidin solution (Avidin/Biotin Blocking Kit, Vector Laboratories, Newark, CA, USA), treated with a 3% hydrogen peroxide solution, and blocked with Serum‐Free Protein Block (Agilent Technologies). The sections were then incubated at 4°C overnight with a HIF‐2α antibody (polyclonal rabbit anti‐HIF‐2α; dilution 1:5000; NB100‐122; Bio‐Techne), diluted in 1% BSA/PBS. The next day, the sections were washed with PBS, incubated with donkey anti‐rabbit HRP‐linked antibody (dilution 1:500; CS7074; Cell Signaling Technology) for 45 min at room temperature and treated with the TSA Plus Biotin Kit (dilution 1:100, 15 min, Akoya Biosciences) for signal amplification. Next, the sections were incubated with streptavidin‐HRP (Abcam, Cambridge, UK) for 30 min and signals were detected using a freshly prepared 3,3′‐diaminobenzidine (DAB) solution (DAB Peroxidase Substrate Kit, Vector Laboratories). Dako Glycergel (Agilent Technologies) was used as mounting medium.

### Microscopy

The RNAscopes and immunohistochemical stainings were captured with a Zeiss Axio Observer.Z1 microscope equipped with both, an Axiocam 506 Mono fluorescence camera and an Axiocam 305 Brightfield camera. A 20x objective lens (Plan‐Apochromat 20x/0.8) and a Colibri 7 light source were used for imaging (Zeiss, Jena, Germany). The following filters were used: filter set 43‐Cy3 (EX BP 545/25; EM BP 605/70), filter set 50‐Cy5 shift free (EX BP 640/30; EM BP 690/50), filter set 96 HE BFP (EX BP 390/40; EM BP 450/40) and filter set 115‐Cy7 (EX BP 710/87; EM BP 814/91) (Zeiss). Overview images of transverse kidney sections were generated by combining tile images. For detailed fluorescence images, 10–12 z‐stack images were captured using the Apotome.2 system (Zeiss), deconvoluted and combined using the ‘maximum projection’ tool. The images in the same figure were captured with identical light intensities and exposure times and were modified in the same way.

### Image analysis

ImageJ software was used to determine the number of target mRNA‐positive cells per kidney section. The analysis was performed on a minimum of six subjects per genotype. For each data point, the number of cells positive for the target mRNA was counted and labelled on three sections per kidney using the ‘cell counter’ tool. Then, the mean values were calculated.

The Intellesis software (Zeiss) was used for automated image analysis to determine cortical renin expression. First, the zonal boundaries were defined. Then, renin expression was determined in the cortex and normalized to the area of the nuclei. Segmentation was achieved through background subtraction using the rolling ball method to ensure the inclusion of all RNAscope signals. For renin, pixel intensity values ranging from 1500 to the maximum measurable intensity (16,384) were included, with a tolerance level set at 1%. A radius of 30 pixels was used for the rolling ball algorithm to optimize segmentation accuracy. No size exclusion criteria were implemented during the analysis to ensure the inclusion of all detected RNAscope signals. A total of at least six representative images per genotype and experimental condition were processed and analysed.

### Statistical analyses

For data analysis, GraphPad Prism 10.6.1 (GraphPad Software, Boston, MA, USA) was used. All data are presented as means ± SD. Statistical significance was determined using a one‐way ANOVA with Tukey's correction or an unpaired Student's *t* test, with Welch's correction, two‐tailed as stated in the Results section. The Results section also states the *P*‐values and group sizes. After correction for multiple testing, *P* ≤ 0.05 was considered statistically significant.

## Results

### PHD2 and PHD2/PHD3 deficiency do not induce EPO production in preglomerular VSMCs but only in interstitial contractile pericytes under basal conditions

To analyse the significance of PHD2 and/or PHD3 for the endocrine function of preglomerular pericyte‐like VSMCs, a mouse model with tamoxifen‐inducible Cre recombinase driven by the promotor for smooth muscle myosin heavy chain (SMMHC^CreERT2^), a gene highly expressed in VSMCs, was used to delete PHD2 and/or PHD3. In addition, interstitial contractile pericytes – a subpopulation of PDGFR‐β^+^ fibroblasts predominantly located in the outer medulla along the vasa recta – also express SMMHC (Broeker et al., 2020). Thus, the effect of the SMMHC cell‐specific deletion of the PHDs was also analysed in these interstitial contractile pericytes.

**Figure 1:**
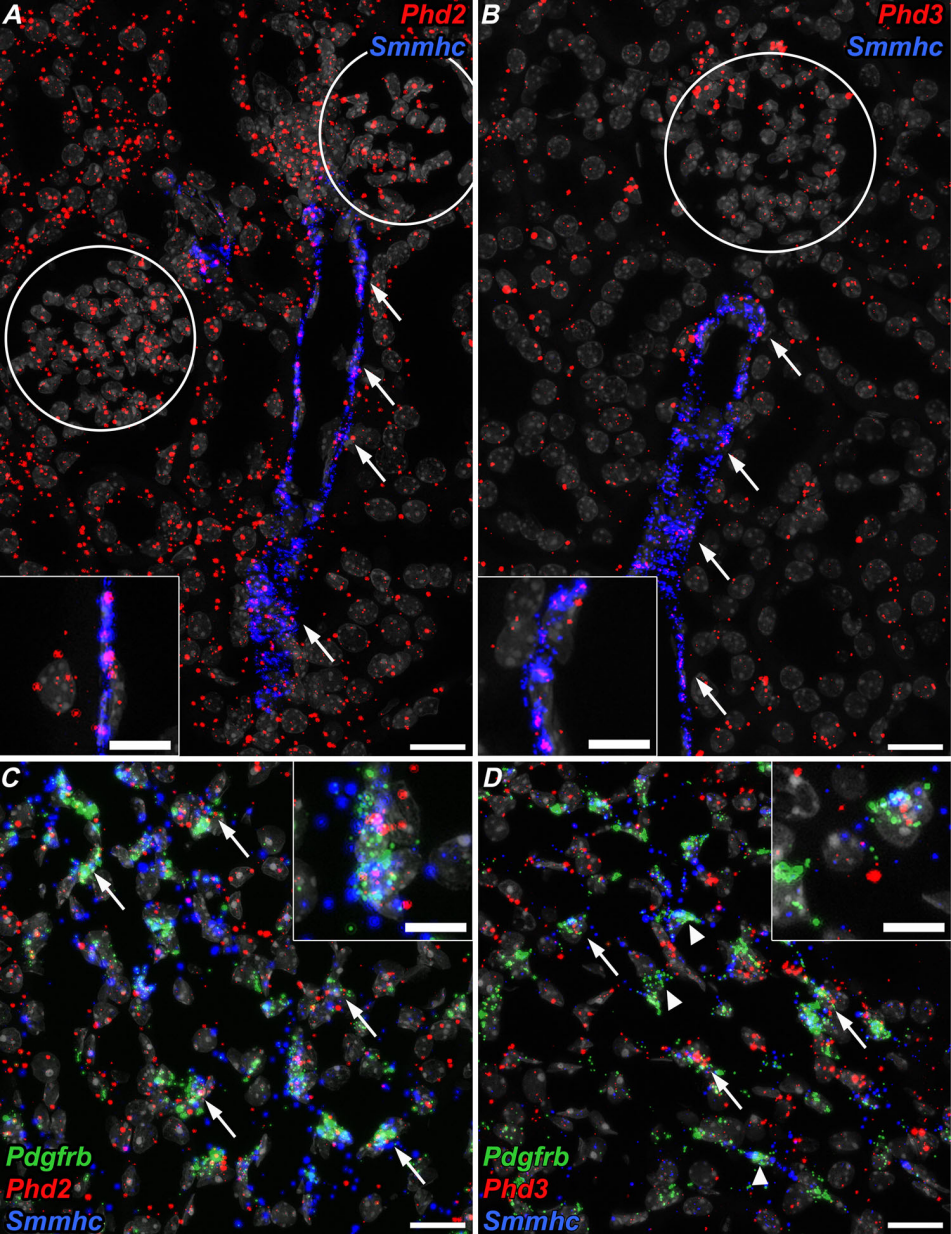
*Phd2* and *Phd3* mRNA expression in preglomerular *Smmhc^+^* pericyte‐like vascular smooth muscle cells and interstitial contractile *Smmhc/Pdgfrb^+^* pericytes of wild‐type mice *A* and *B*, cortical details of kidney sections from a wild‐type mouse showing an RNAscope for *Phd2* or *Phd3* (red) and *Smmhc* (blue) transcripts. Both, *Phd2* (*A*) and *Phd3* (*B*) mRNA expression could be detected in *Smmhc* ^+^ vascular smooth muscle cells (arrows). Insets in the lower left corner show some VSMCs at a higher magnification. *C* and *D*, medullary details of kidney sections from a wild‐type mouse showing multiplex RNAscope for *Phd2* or *Phd3* (red), *Smmhc* (blue) and *Pdgfrb* (green) mRNA. *Phd2* (*C*) mRNA expression was detected in all *Smmhc/Pdgfrb^+^* contractile pericytes (exemplarily highlighted by arrows). *Phd3* (*D*) mRNA expression could only be detected in part of the contractile pericytes. Arrows highlight some *Phd3^+^* pericytes, while arrowheads indicate some examples of interstitial contractile pericytes negative for *Phd3* transcripts. Insets in the upper right corner show some interstitial contractile pericytes at a higher magnification. Circles indicate glomeruli. Nuclei were counterstained with DAPI (grey). Scale bars 20 µm; scale bars in insets: 10 µm.

The expression patterns of *Phd2* and *Phd3* transcripts were analysed in preglomerular *Smmhc^+^* VSMCs and interstitial contractile pericytes of wild‐type mice. Using multiplex RNAscope, *Phd2* or *Phd3* mRNA was visualized together with *Smmhc* and *Pdgfrb* transcripts to identify VSMCs and interstitial contractile pericytes, respectively. *Smmhc^+^* VSMCs were all positive for *Phd2* and *Phd3* mRNA, although expression levels of *Phd3* mRNA seemed lower per cell compared to *Phd2* (Fig. 1*A* and *B*). In addition, all *Smmhc/Pdgfrb^+^* interstitial contractile pericytes were positive for *Phd2* transcripts (Fig. 1*C*), while *Phd3* mRNA expression was only detected in about 50–60% of these cells (Fig. 1*D*).

As VSMCs are positive for both *Phd2* and *Phd3* transcripts, their functional significance was analysed by deleting PHD2 and/or PHD3 specifically in SMMHC^+^ cells and evaluating renal renin and EPO production.

**Figure 2:**
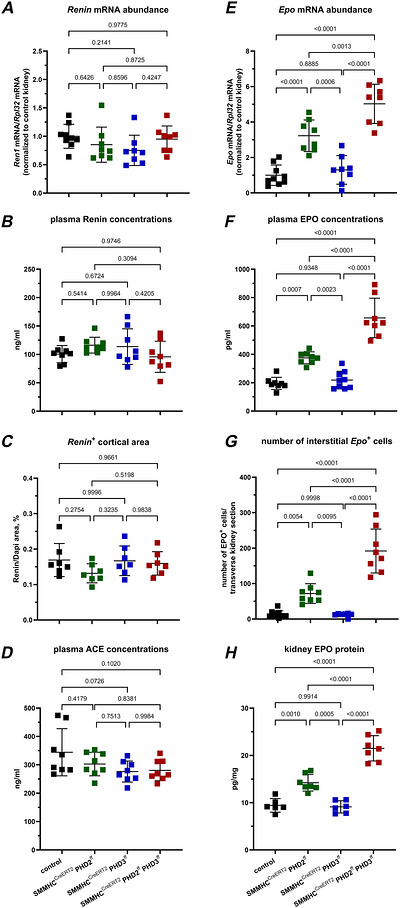
Renal renin and EPO production in control mice and mice with SMMHC‐specific PHD deletions *A*, renal *Renin* mRNA abundances were determined by RT‐qPCR. Values are displayed relative to the ribosomal protein L32 and normalized to the control kidney. *B*, plasma renin concentrations were determined by ELISA. *C*, automated quantification of the proportion of the *Renin^+^* cortical area per transverse kidney section. *D*, plasma angiotensin‐converting enzyme (ACE) concentrations were determined by ELISA. *E*, renal *Epo* mRNA abundances were determined by RT‐qPCR. Values are displayed relative to the ribosomal protein L32 and normalized to the control kidney. *F*, plasma EPO concentrations were determined by ELISA. *G*, the number of interstitial *Epo* ^+^ cells per transverse kidney section was determined using ImageJ. *H*, renal EPO protein concentrations were determined in kidney lysates as pg/mg of total protein. Statistical significance between groups with different genotypes was determined using one‐way ANOVA with Tukey's correction. All *P*‐values are stated above the lines. Values are means ± SD of *n* ≥ 6 mice per genotype.

**Figure 3:**
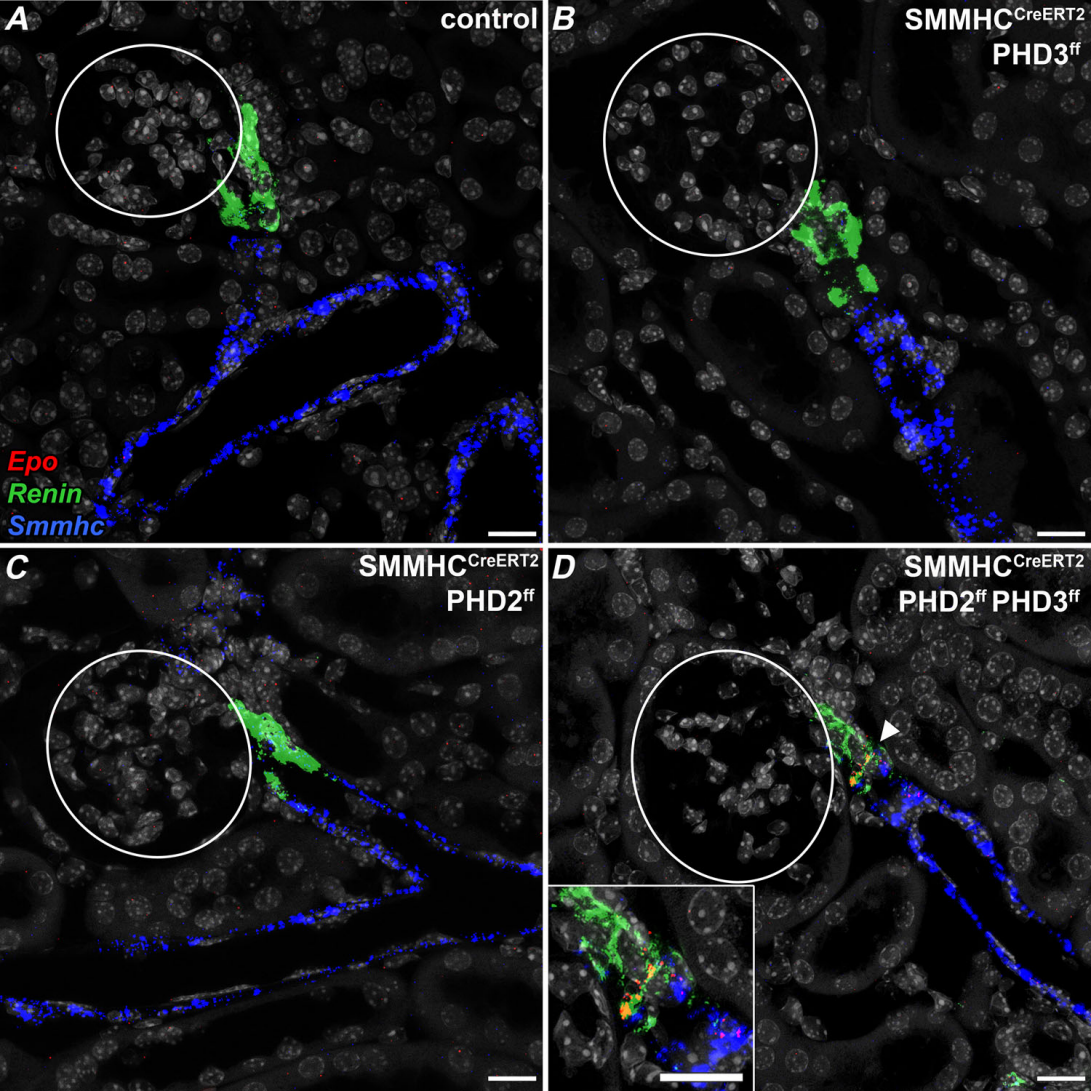
*Epo* and *Renin* mRNA expression in preglomerular VSMCs of control mice and mice with SMMHC‐specific PHD deletions Cortical details showing multiplex RNAscope for *Epo* (red), *Renin* (green) and *Smmhc* (blue) transcripts on kidney sections of control (*A*), SMMHC^CreERT2^ PHD3^ff^ (*B*), SMMHC^CreERT2^ PHD2^ff^ (*C*), or SMMHC^CreERT2^ PHD2^ff^ PHD3^ff^ (*D*) mice. Circles indicate glomeruli. The arrowhead highlights *Epo* mRNA expression in a juxtaglomerular cell (*D*) that is shown in higher magnification in the lower left corner. Nuclei were counterstained with DAPI (grey). Scale bars: 20 µm.

In the control group, mean plasma renin concentrations were 100.6 ± 15.1 ng/ml. In the SMMHC^CreERT2^ PHD2^ff^ (PHD2‐KO), SMMHC^CreERT2^ PHD3^ff^ (PHD3‐KO) or SMMHC^CreERT2^ PHD2^ff^ PHD3^ff^ (PHD2/PHD3‐KO) mice, no changes in *Renin* mRNA expression or plasma renin concentrations were detected compared to control animals. The mean plasma renin concentrations in these groups ranged from 95.7 ± 27.3 to 116.2 ± 14.0 ng/ml (Fig. 2*A* and *B*). Consistent with the qPCR data, RNAscope analysis confirmed the unchanged localization and abundance of *RRenin* mRNA expression in afferent arterioles across all genotypes (Figs 2*C* and 3). Because HIF‑dependent upregulation of ACE expression has been reported in (smooth) muscle cells (Yin et al., 2024; Zhang et al., 2009), and ACE represents a key enzyme of the renin–angiotensin system alongside renin, we assessed whether SMMHC‑specific PHD deletions alter plasma ACE concentrations. Across all knockout models, plasma ACE levels remained unchanged compared to controls (Fig. 2*D*).

In contrast, *Epo* mRNA expression was significantly upregulated in PHD2‐KO mice (about 3‐fold), whereas PHD3 deletion alone had no effect. Notably, after SMMHC cell‐specific PHD2/PHD3 codeletion, *Epo* mRNA abundances were significantly higher than in SMMHC^CreERT2^ PHD2^ff^ mice (about 5‐fold compared to controls) (Fig. 2*E*). Consistent with the transcriptional data, renal EPO protein concentrations increased from 9.4 ± 1.4 pg/mg in control mice to 14.2 ± 1.8 pg/mg in PHD2‑KO mice and to 21.5 ± 2.7 pg/mg in PHD2/PHD3‑KO mice. Correspondingly, plasma EPO levels rose to 375.3 ± 42.1 pg/ml in PHD2‑KO mice and to 656.6 ± 139.3 pg/ml in PHD2/PHD3‑KO mice, compared with 195.1 ± 42.8 pg/ml in controls, resulting in mean haematocrit values of 57.0 ± 4.9% and 68.8 ± 6.4%, respectively (controls: 51.3 ± 2.4%). PHD3‑KO mice showed no changes in renal EPO, plasma EPO or hematocrit compared to the control group (Fig. 2*F* and *H*).

**Figure 4:**
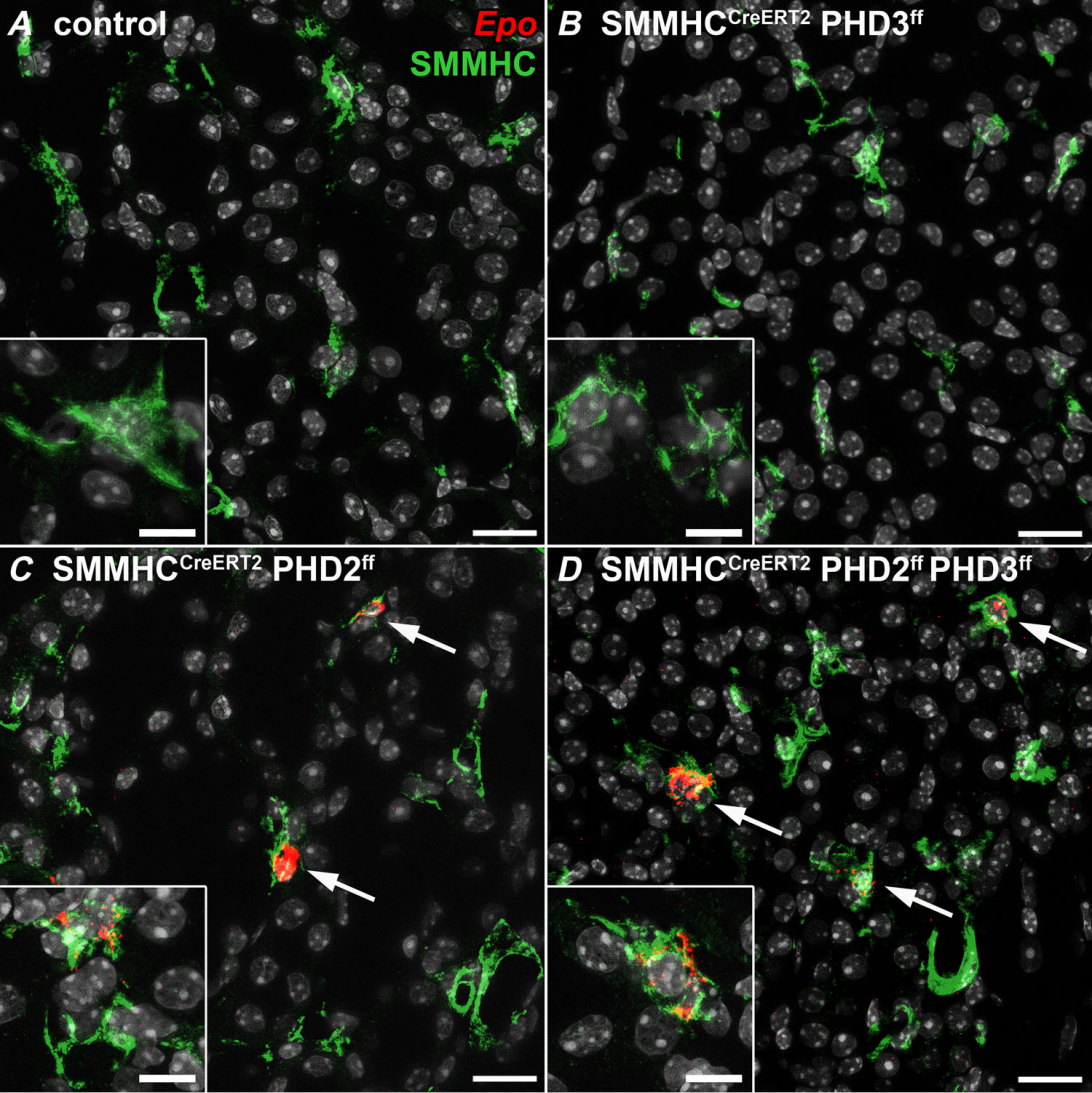
*Epo* mRNA expression in interstitial contractile pericytes in kidneys of control mice and mice with SMMHC‐specific PHD deletions Details of the outer medulla showing RNAscope for *Epo* mRNA (red) in combination with an immunofluorescence staining for SMMHC to visualize interstitial contractile pericytes on kidney sections of control (*A*), SMMHC^CreERT2^ PHD3^ff^ (*B*), SMMHC^CreERT2^ PHD2^ff^ (*C*), or SMMHC^CreERT2^ PHD2^ff^ PHD3^ff^ (*D*) mice. The square boxes in the lower left corner of each image show contractile pericytes at a higher magnification. *Epo* mRNA induction occurred only in PHD2‐KO and PHD2/PHD3‐KO mice. Arrows highlight interstitial contractile *Epo* expressing pericytes. In control and PHD3‐KO mice contractile pericytes do not express *Epo* mRNA. Nuclei were counterstained with DAPI (grey). Scale bars: 20 µm; scale bars in insets: 10 µm.

**Figure 5:**
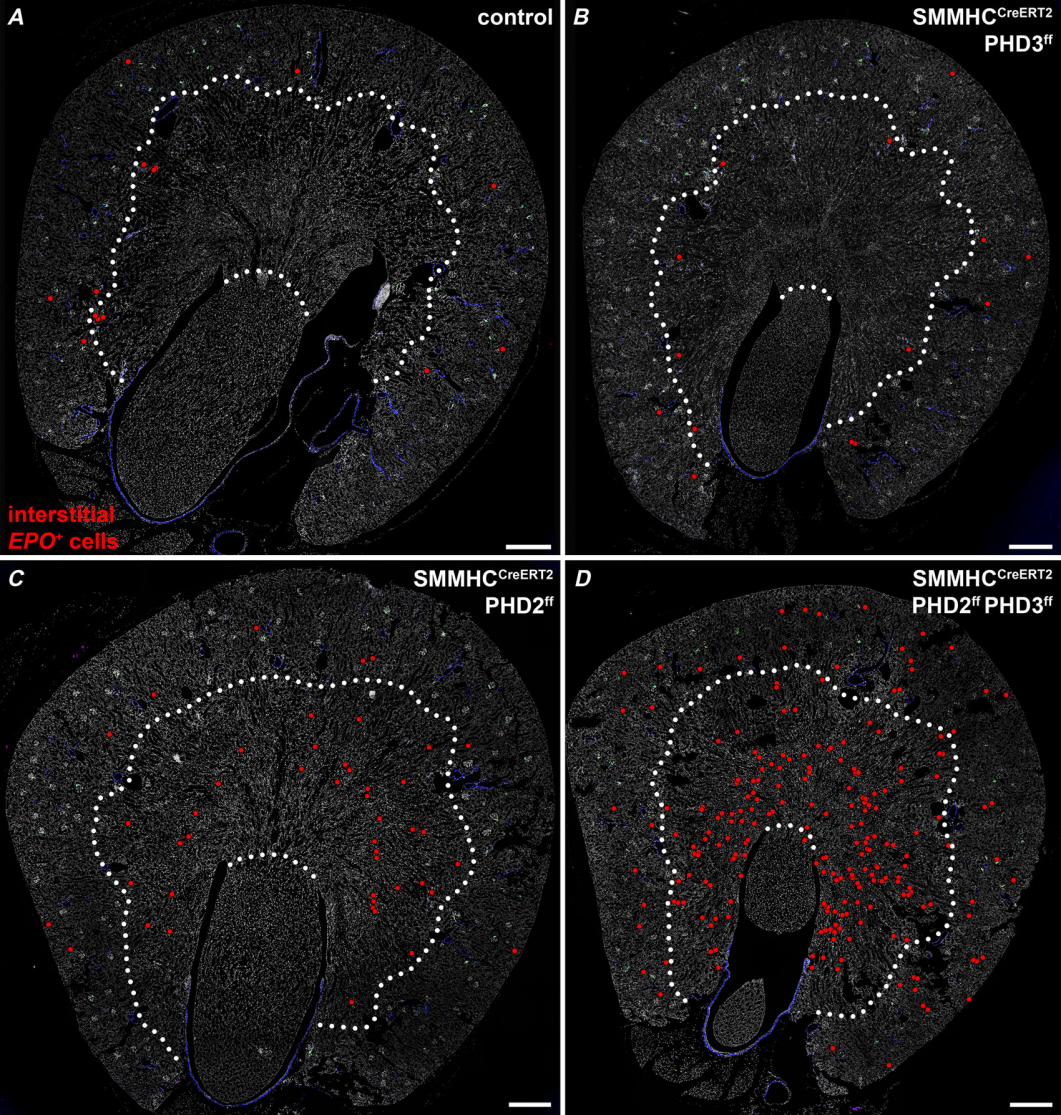
Distribution of interstitial *Epo* mRNA expressing cells in control mice and mice with different SMMHC cell‐specific PHD deletions Overviews of transverse kidney sections showing the expression pattern of interstitial *Epo* mRNA expressing cells (red dots) detected by RNAscope in control mice (*A*) and after SMMHC cell‐specific PHD3 (*B*), PHD2 (*C*), or PHD2/PHD3 (*D*) deletion. Each red dot highlights an interstitial *Epo^+^* cell marked using ImageJ. White dotted lines indicate zonal borders. Nuclei were counterstained with DAPI (grey). Scale bars: 500 µm.

Multiplex RNAscope demonstrated that *Epo* mRNA induction in PHD2‑KO and PHD2/PHD3‑KO mice occurred exclusively in *Smmhc* ^+^/*Pdgfrb* ^+^ contractile pericytes of the outer medulla, and in the latter group also in a small number of cortical interstitial pericytes (Figs 4 and 5). No *Epo* mRNA induction was detected in preglomerular VSMCs of either genotype (Fig. 3*C* and *D*). Only a few juxtaglomerular renin‑producing cells, which exhibit low *Smmhc* mRNA expression, began expressing *Epo* mRNA in PHD2/PHD3‑KO mice (Fig. 3*D*). Consistent with previous observations following renin cell‐specific PHD2/PHD3 deletion (Broeker et al., 2021), *Renin* expression seemed downregulated in these cells. In control and PHD3‑KO mice, *Epo* mRNA was confined to the few native EPO‐producing PDGFR‐β^+^ fibroblasts along the cortico‑medullary border (Fig. 5*A* and *B*).

Quantification of *Epo*‐expressing interstitial pericytes mirrored the RT‐qPCR and renal EPO protein data: PHD2/PHD3‐KO mice displayed significantly more *Epo^+^* interstitial cells per transverse section (191.8 ± 61.8) than PHD2‐KO mice (71.9 ± 27.7). Control and PHD3‐KO mice showed only 15.6 ± 9.8 and 11.9 ± 5.4 *Epo^+^* cells, respectively (Figs 2*G* and 5). For a better overview, all parameters presented here are summarized in Table 4.

**Table 4:** Summary of the measured parameters associated with EPO and renin synthesis

	*Epo* mRNA/*Rpl32* mRNA	Kidney EPO (pg/mg)	Hct (%)	Plasma‐EPO (pg/ml)	*Renin* mRNA/*Rpl32* mRNA	Plasma renin (ng/ml)	Plasma‐ACE (ng/ml)	Systolic blood pressure (mmHg)
Control	1.0 ± 0.6	9.4 ± 1.4	51.3 ± 2.4	195.1 ± 42.8	1.0 ± 0.2	100.6 ± 15.1	344.3 ± 83.0	135.8 ± 6.9
Control + LSE	0.1 ± 0.2	6.5 ± 1.0	43.9 ± 3.7	155.6 ± 26.7	13.3 ± 2.8	1726.9 ± 340.2	453.4 ± 134.0	100.5 ± 5.4
SMMHC^Cre^ PHD2 ^ff^	3.2 ± 0.9	14.2 ± 1.8	57.0 ± 4.9	375.3 ± 42.1	0.9 ± 0.3	116.2 ± 14.0	302.8 ± 41.5	n.d.
SMMHC^Cre^ PHD2 ^ff^ + LSE	2.4 ± 0.6	13.4 ± 2.3	56.6 ± 2.1	357.0 ± 68.1	12.0 ± 3.4	1595.8 ± 313.9	398.4 ± 165.0	n.d.
SMMHC^Cre^ PHD3 ^ff^	1.3 ± 0.8	9.1 ± 1.3	49.1 ± 1.8	218.4 ± 62.4	0.8 ± 0.3	113.8 ± 31.3	276.2 ± 37.1	n.d.
SMMHC^Cre^ PHD3 ^ff^ + LSE	0.2 ± 0.3	7.1 ± 1.5	42.8 ± 4.1	189.0 ± 45.0	11.3 ± 1.8	1532.5 ± 490.0	373.9 ± 109.9	n.d.
SMMHC^Cre^ PHD2 ^ff^ PHD3 ^ff^	5.0 ± 1.1	21.5 ± 2.7	68.8 ± 6.4	656.6 ± 139.3	1.0 ± 0.2	95.7 ± 27.3	280.6 ± 36.7	137.4 ± 5.9
SMMHC^Cre^ PHD2 ^ff^ PHD3 ^ff^ + LSE	8.1 ± 1.8	30.7 ± 3.9	75.1 ± 4.8	979.0 ± 98.4	6.3 ± 1.6	779.1 ± 90.3	375.4 ± 69.3	97.8 ± 3.7

All data are presented as means ± SD. n.d., not determined.

**Figure 6:**
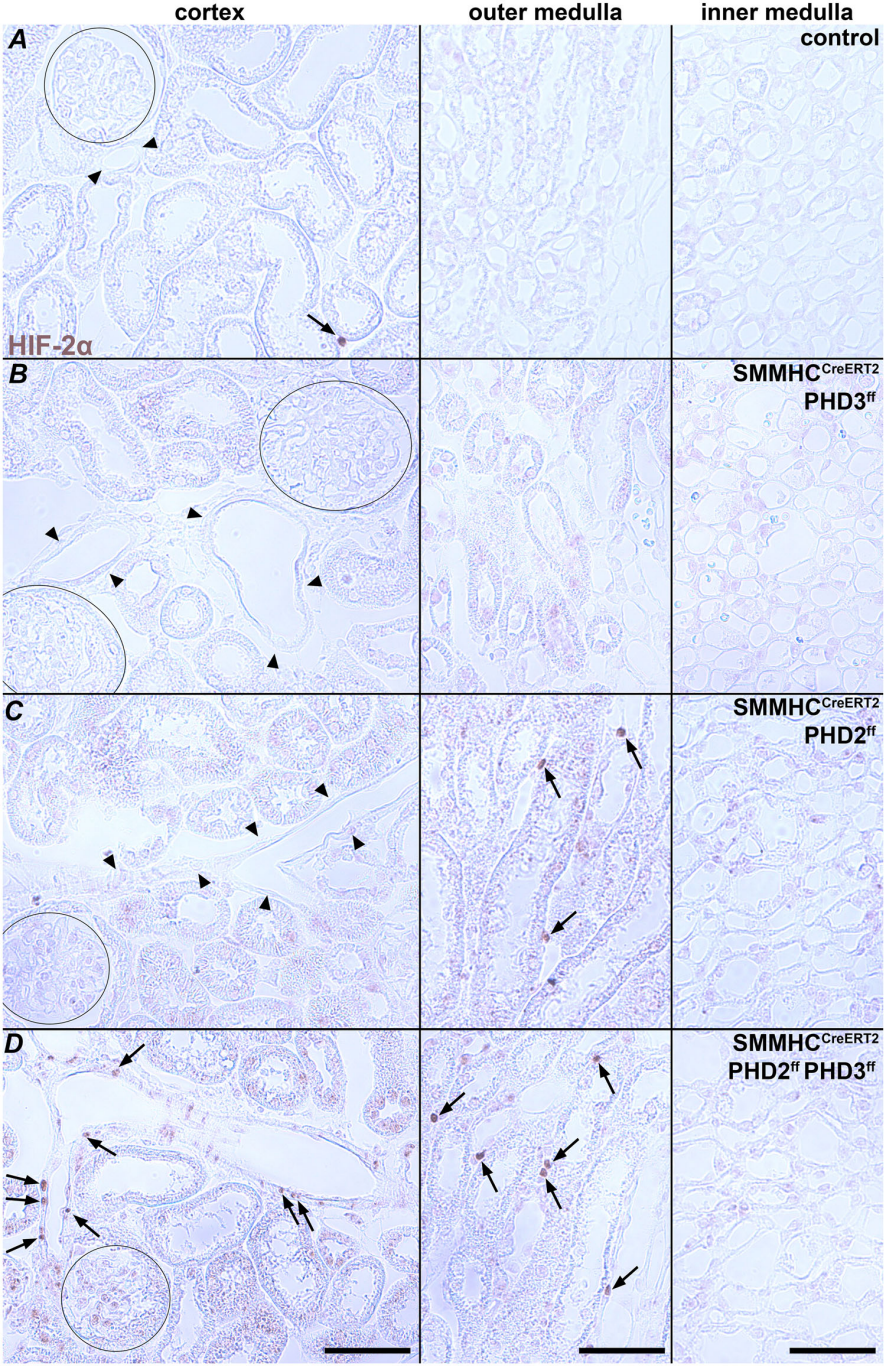
HIF‐2α stabilization on kidney sections of control mice and mice with SMMHC‐specific PHD deletions Details of the cortex (left), outer medulla (middle) and inner medulla (right) of kidney sections of the respective genotypes showing immunohistochemical staining for HIF‐2α (brown nuclei). *A*, in control mice, no HIF‐2α positive nuclei were detected in VSMCs (arrowheads) or in interstitial pericytes of the outer medulla. Only occasional HIF‐2α positive interstitial cells were observed along the corticomedullary border (arrow). *B*, similarly, SMMHC^CreERT2^ PHD3^ff^ mice showed no detectable HIF‑2α positive nuclei in VSMCs (arrowheads) or interstitial pericytes. *C*, in SMMHC^CreERT2^ PHD2^ff^ mice, VSMCs remained negative for HIF‑2α (arrowheads), but several HIF‐2α positive nuclei were present within the outer medullary interstitium (arrows). *D*, in SMMHC^CreERT2^ PHD2^ff^ PHD3^ff^ mice, clear HIF‐2α positive nuclei were detected both in preglomerular VSMCs and within the interstitium of the outer medulla (arrows). Circles indicate glomeruli. Scale bars (apply to the entire column): 50 µm.

To analyse whether the lack of EPO induction in preglomerular VSMCs is due to inadequate HIF‐2α stabilization, we investigated HIF‐2α stabilization by immunohistochemistry. In kidneys of control and PHD3‐KO mice, neither preglomerular VSMCs nor interstitial contractile pericytes of the outer medulla displayed detectable HIF‐2α staining (Fig. 6*A* and *B*). Sporadically, few HIF‐2α positive interstitial cells could be observed in the deeper cortex or at the corticomedullary border. Following SMMHC‐specific PHD2 deletion, VSMCs remained negative for HIF‐2α (Fig. 6*C*). However, some interstitial cells in the outer medulla were clearly positive for HIF‐2α in addition to the sporadic interstitial signal along the corticomedullary border. Co‐deletion of PHD2 and PHD3 resulted in robust HIF‐2α stabilization in preglomerular VSMCs (Fig. 6*D*) and further increased the number of HIF‐2α‐positive interstitial cells in the outer medulla compared with PHD2‐KO mice. The inner medulla was devoid of HIF‐2α staining in all genotypes.

### Low salt diet combined with the ACE‐inhibitor enalapril induces EPO instead of renin in preglomerular VSMCs deficient for PHD2 and PHD3

Since preglomerular VSMCs revert to a renin‑producing phenotype in response to low blood pressure, salt deficiency or dehydration, the role of PHD2 and PHD3 in the transformation of these VSMCs into renin cells was examined. A well‐established approach to experimentally stimulate renin production in preglomerular VSMCs and extraglomerular mesangial cells is treatment with a low‐salt diet combined with the ACE‐inhibitor enalapril, an antihypertensive drug (Neubauer et al., 2013; Pentz et al., 2012). Accordingly, following induction of SMMHC‑specific PHD deletions, the mice were placed on a low‐salt diet and given enalapril in their drinking water (LSE) for 14 days.

**Figure 7:**
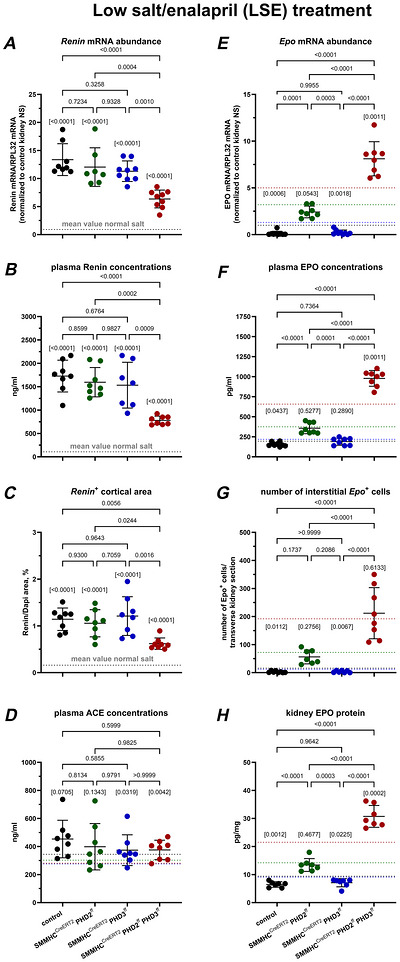
Renal renin and EPO production in control mice and mice with SMMHC‐specific PHD deletions after low salt/enalapril (LSE) treatment *A*–*C*, renal *Renin* mRNA abundances, plasma renin concentrations and the *Renin^+^* area in the cortex of kidney sections. For comparison, the grey dotted line shows the average of the mean values of the untreated groups that were fed a normal salt diet. *A*, renal *Renin* mRNA abundances were determined by RT‐qPCR. Values are displayed relative to the ribosomal protein L32 and normalized to the untreated control kidney. *B*, plasma renin concentrations were determined by ELISA. *C*, automated quantification of the proportion of the *Renin^+^* cortical area per transverse kidney section. *D*, plasma angiotensin‐converting enzyme (ACE) concentrations were determined by ELISA. For comparison, black, green, blue and red dotted lines indicate the mean values for each group on a normal salt diet. *E–H*, renal *Epo* mRNA abundances, plasma EPO concentrations, the number of interstitial *Epo*‐expressing cells per transverse kidney section and renal EPO protein concentrations. For comparison, black, green, blue and red dotted lines indicate the mean values for each group on a normal salt diet. *E*, renal *Epo* mRNA abundances were determined by RT‐qPCR. Values are displayed relative to the ribosomal protein L32 and normalized to the untreated control kidney. *F*, plasma EPO concentrations were determined by ELISA. *G*, the number of interstitial *Epo* ^+^ cells per transverse kidney section was determined using ImageJ. *H*, renal EPO protein concentrations were determined in kidney lysates as pg/mg of total protein. Statistical significance between groups with different genotypes was determined using one‐way ANOVA with Tukey's correction. *P*‐values are stated above the lines. Statistical significance between the LSE and normal salt (NS = untreated) groups (see Fig. 2) of the same genotype were determined using an unpaired *t* test with Welch's correction, two‐tailed. The respective *P*‐values are stated in brackets. Values are means ± SD of *n* ≥ 7 mice per genotype.

In control mice, LSE treatment induced a 13‑fold increase in *Renin* mRNA abundance compared with untreated animals on a normal‑salt diet (Figs 2 and 7*A*), resulting in plasma renin concentrations of 1726.9 ± 340.2 ng/ml (*vs*. 100.6 ± 15.1 ng/ml) (Fig. 7*B*). LSE‑treated SMMHC^CreERT2^ PHD2^ff^ and SMMHC^CreERT2^ PHD3^ff^ mice displayed similar increases in renal *Renin* mRNA expression, with mean plasma renin levels of 1595.8 ± 313.9 and 1532.5 ± 490.0 ng/ml, respectively.

In SMMHC^CreERT2^ PHD2^ff^ PHD3^ff^ mice, LSE treatment also stimulated *Renin* mRNA expression. However, the increase was only 7‐fold – significantly lower than in LSE‐treated controls (Fig. 7*A*) – and accompanied by markedly reduced plasma renin concentrations (779.1 ± 90.3 ng/ml) compared to LSE‐treated controls (Fig. 7*B*).

**Figure 8:**
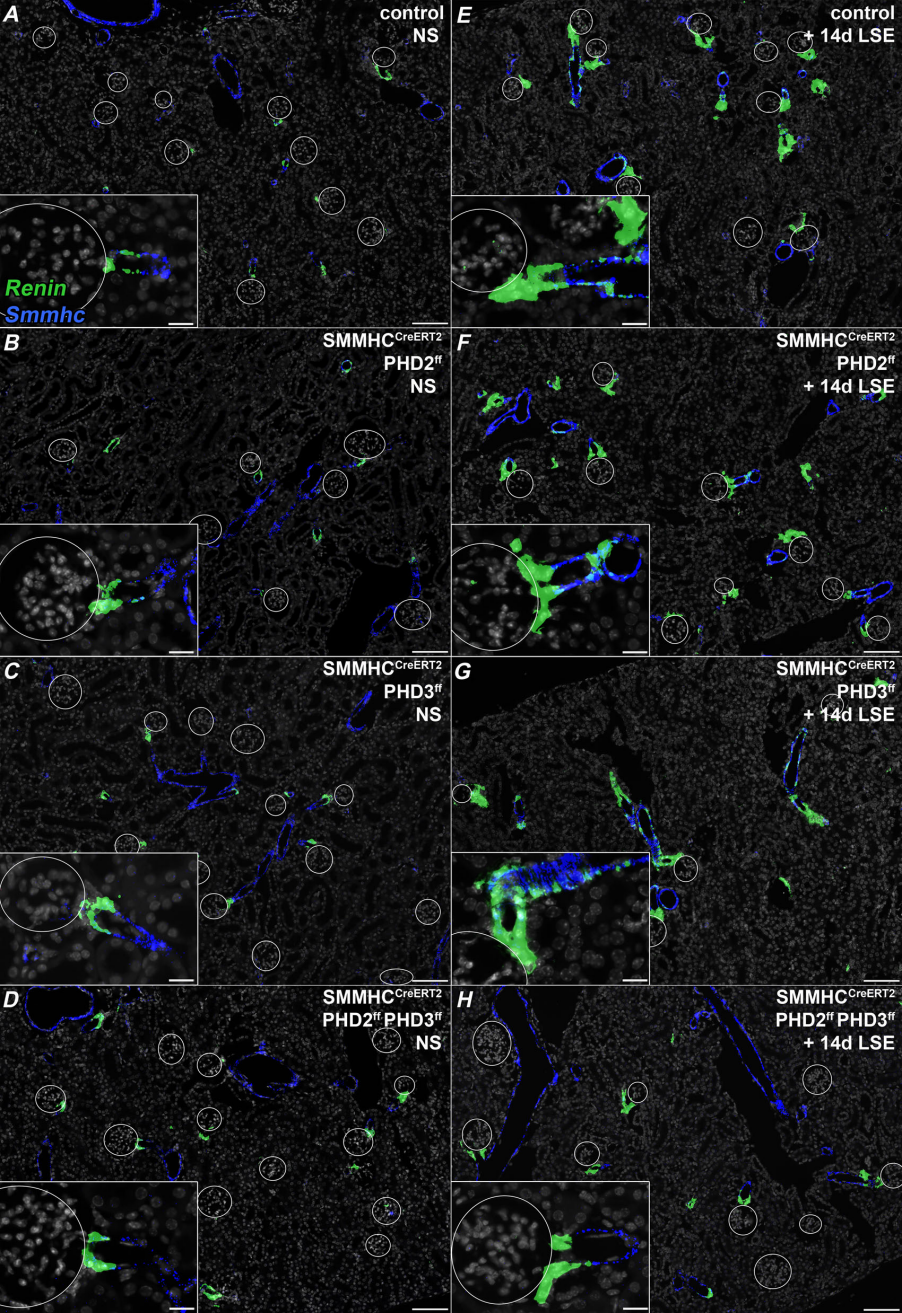
Extent and pattern of *Renin* mRNA expression after normal‐salt diet or low‐salt diet in combination with enalapril (LSE) on kidney sections from control animals and animals with different PHD deletions *A*–*D*, co‐RNAscope showing *Renin* (green) and *Smmhc* (blue) mRNA expression on kidney sections from mice of the different analysed mouse models on a normal salt diet. *E*–*H*, co‐RNAscope showing *Renin* (green) and *Smmhc* (blue) mRNA expression on kidney sections from the analysed mouse models on an LSE diet. Square insets in the lower left corner of each image display a glomerulus with its afferent arteriole at higher magnification. Circles highlight glomeruli. Nuclei were counterstained with DAPI (grey). Scale bars: 100 µm; scale bars in insets: 20 µm.

RNAscope analysis confirmed the characteristic induction of renin synthesis in preglomerular VSMCs and extraglomerular mesangial cells in LSE‐treated control mice (Fig. 8*E*), with similar patterns in PHD2‐ and PHD3‑deficient mice (Fig. 8*F* and *G*). In contrast, LSE‐treated SMMHC^CreERT2^ PHD2^ff^ PHD3^ff^ mice showed induction of *Renin* mRNA expression predominantly in extraglomerular mesangial cells, with little to no transformation along preglomerular vessels (Fig. 8*H*). Automated quantification of the cortical renin‑positive area per kidney section further confirmed the markedly reduced number of LSE‐induced renin cells in the PHD2/PHD3‐KO animals (Fig. 7*C*).

Plasma ACE concentrations were elevated by approximately 100 ng/ml in all LSE‐treated groups compared with ACE levels under a normal‑salt diet. However, there was no difference in plasma ACE concentrations between the LSE‐treated controls and the respective LSE‑treated PHD‐KO groups (Fig. 7*D*).

Because renin induction was significantly reduced in PHD2/PHD3‐KO animals compared with their respective controls, systolic blood pressure was measured in both groups under NS diet and LSE treatment. Under NS conditions, the controls and PHD2/PHD3‐KO animals displayed comparable systolic blood pressure values (135.8 ± 6.9 *vs*. 137.4 ± 5.9 mmHg; *P* = 0.5154; *n* = 7). LSE treatment lowered systolic blood pressure in both groups by approximately 40 mmHg, resulting in values of 100.5 ± 5.4 and 97.8 ± 3.7 mmHg, respectively, with no significant difference between controls and PHD2/PHD3‐KO animals (*P* = 0.0851; *n* = 7).

**Figure 9:**
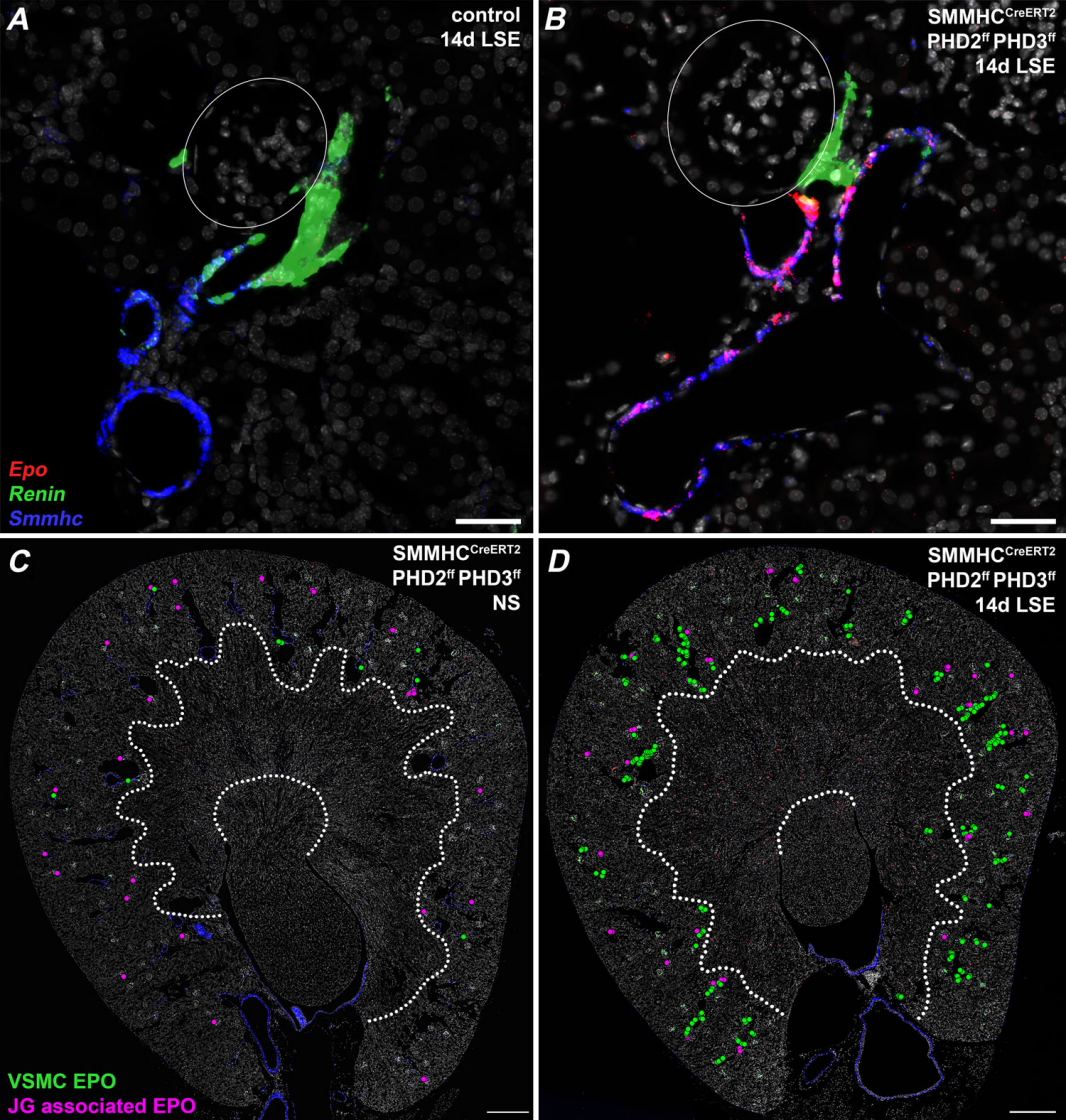
*Renin* and *Epo* mRNA expression in preglomerular VSMCs of control and SMMHC^CreERT2^ PHD2^ff^ PHD3^ff^ mice after low salt/enalapril (LSE) treatment as well as the distribution of vascular *Epo* mRNA expression in kidneys of SMMHC^CreERT2^ PHD2^ff^ PHD3^ff^ mice without and with LSE treatment *A* and *B*, cortical details of an RNAscope for *Epo* (red), *Renin* (green) and *Smmhc* (blue) transcripts on kidney sections of control and PHD2/PHD3‐KO mice after LSE diet. In control mice LSE treatment led to a strong induction of *Renin* mRNA expression in VSMCs and extraglomerular mesangial cells (*A*). In PHD2/PHD3‐KO mice, *Renin* mRNA induction was restricted to extraglomerular mesangial cells. VSMCs no longer transcribed *Renin* mRNA but instead started to express *Epo* mRNA (*B*). Circles indicate glomeruli. Nuclei were counterstained with DAPI (grey). Scale bars: 50 µm. *C* and *D*, overviews of transverse kidney sections showing the expression pattern of *Epo* mRNA‐expressing preglomerular VSMCs (green dots) and *Epo* mRNA‐expressing juxtaglomerular (JG) cells (pink dots) in PHD2/PHD3‐KO mice fed a NS or LSE diet. The LSE treatment resulted in a clear induction of *Epo* mRNA expression in preglomerular VSMCs (*D*), while in PHD2/PHD3‐KO mice without LSE diet, *Epo* mRNA induction was mostly restricted to JG cells (*C*). Dotted lines indicate zonal borders. Nuclei were counterstained with DAPI (grey). Scale bars: 500 µm.

Renal *Epo* mRNA abundances, renal EPO protein concentrations (30.7 ± 3.9 pg/mg) and plasma EPO concentrations (979.0 ± 98.4 pg/ml) of the LSE‐treated SMMHC^CreERT2^ PHD2^ff^ PHD3^ff^ mice were significantly increased compared to PHD2/PHD3‐KO animals without LSE treatment (kidney EPO: 21.5 ± 2.7 pg/mg; plasma EPO: 656.6 ± 139.3 pg/ml) (Fig. 7*E*, *F* and *H*). Mean haematocrit values increased to 75.1 ± 4.8%. The additional *Epo* mRNA expression could not be allocated to further interstitial pericytes. On the contrary, RNAscope analysis revealed distinct *Epo* mRNA signals in VSMCs along the preglomerular vessels in the kidneys of LSE‐treated SMMHC^CreERT2^ PHD2^ff^ PHD3^ff^ mice (Fig. 9*B*). Per transverse kidney section, 257 ± 52 *Epo* ^+^ VSMCs were detectable. In SMMHC^CreERT2^ PHD2^ff^ PHD3^ff^ mice on a normal salt diet, only 42 ± 15 EPO^+^ VSMCs per transverse kidney section were detected restricted to the juxtaglomerular position (Fig. 9*C* and *D*). The number of interstitial *Epo* ^+^ pericytes remained unchanged with an average of 211.9 ± 91.1 cells after LSE treatment compared to 191.8 ± 61.8 cells on the NS diet (Fig. 7*G*).

In control and PHD3‐KO mice the number of interstitial *Epo* ^+^ cells per kidney section was reduced after LSE treatment (2.8 ± 3.1 and 3.7 ± 3.8 cells per kidney section, respectively) compared to normal salt diet (Fig. 7*G*). In parallel, *Epo* mRNA expression levels and kidney EPO protein concentrations (6.5 ± 1.0 and 7.1 ± 1.5 pg/mg, respectively) were significantly downregulated compared to the respective normal salt fed‐groups (Fig. 7*E* and *H*). This resulted in reduced plasma EPO concentrations of about 155.6 ± 26.7 pg/ml in LSE‐treated controls and 189.0 ± 45.0 pg/ml in SMMHC^CreERT2^ PHD3^ff^ mice (Hct in controls: 43.9% ± 3.7%; Hct in PHD3‐KO: 42.8% ± 4.1%). In LSE‐treated SMMHC^CreERT2^ PHD2^ff^ mice renal EPO production was not significantly changed compared to the NS‐group (kidney EPO: 13.4 ± 2.3 pg/mg; plasma EPO: 357.0 ± 68.1 pg/ml; Hct: 56.6% ± 2.1%) (Fig. 7*E*–*H*). For greater clarity, all parameters presented here are summarized in Table 4.

**Figure 10:**
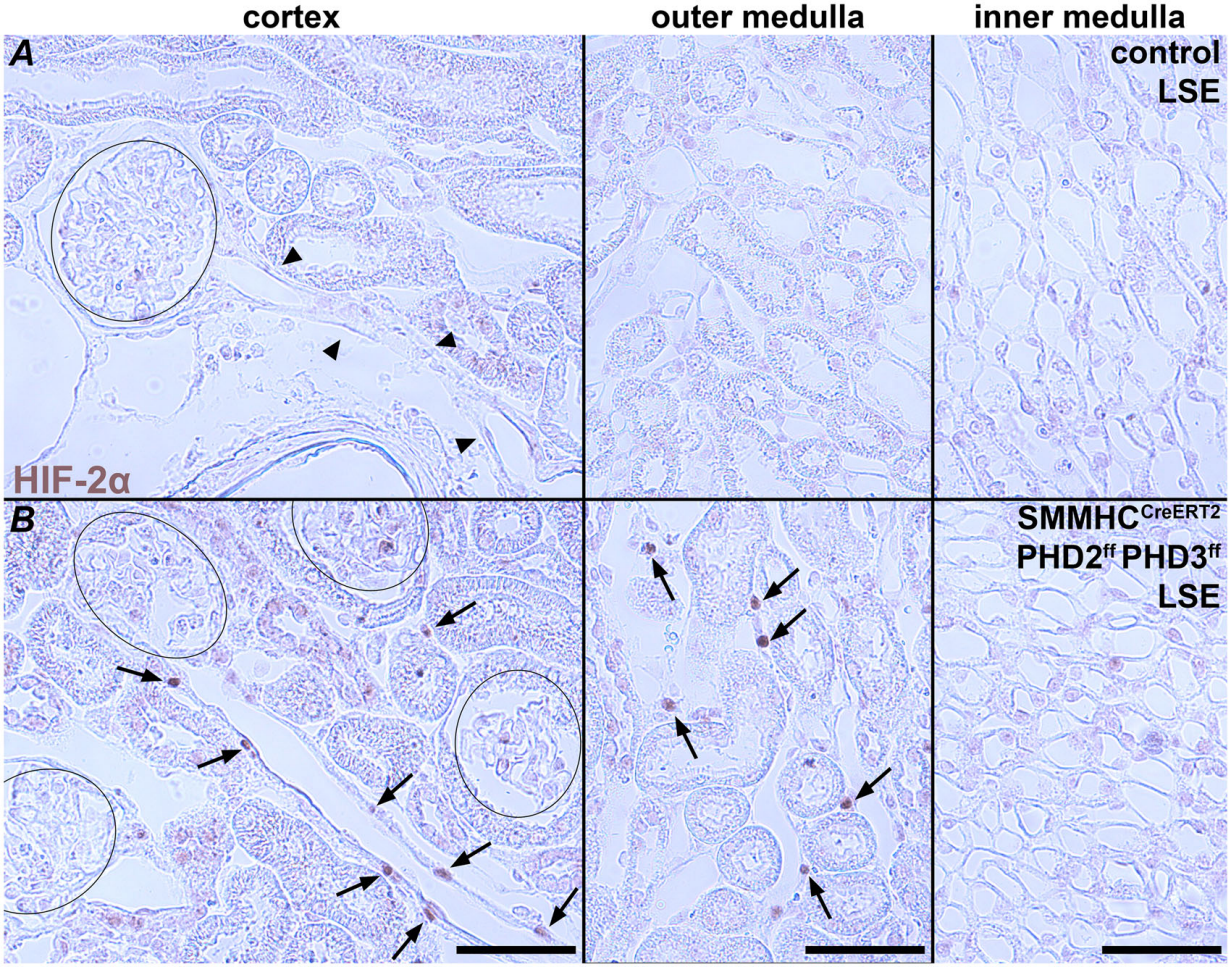
HIF‐2α stabilization in kidney sections of control mice and SMMHC^CreERT2^ PHD2^ff^ PHD3^ff^ mice after 14 days of LSE treatment Representative details of the cortex (left), outer medulla (middle) and inner medulla (right) showing immunohistochemical staining for HIF‐2α (brown nuclei). *A*, in control mice, no HIF‐2α positive nuclei could be detected in preglomerular VSMCs (arrowheads) or in interstitial pericytes of the outer medulla. *B*, in SMMHC^CreERT2^ PHD2^ff^ PHD3^ff^ mice, distinct HIF‐2α positive nuclei were present in preglomerular VSMCs and within the outer medullary interstitium (arrows). Circles indicate glomeruli. Scale bars (apply to the entire column): 50 µm.

Based on the finding that LSE treatment induced *Epo* mRNA expression along the preglomerular vessels, HIF‑2α stabilization was analysed in LSE‑treated control kidneys as well as in kidneys from LSE‑treated SMMHC^CreERT2^ PHD2^ff^ PHD3^ff^ mice. Compared with HIF‑2α immunohistochemistry in the respective genotypes under NS conditions (see Fig. 6*A* and *D*), no obvious changes in the distribution or intensity of HIF‑2α positive nuclei were detected in kidneys from LSE‑treated control animals or PHD2/PHD3‐KO mice. Preglomerular VSMCs and medullary interstitial pericytes remained negative for HIF‑2α in LSE‐treated controls (Fig. 10*A*). Occasionally, very few HIF‑2α positive interstitial cells were observed in the corticomedullary region (even less than under NS conditions). In kidneys of LSE‑treated PHD2/PHD3‐KO animals, HIF‑2α positive nuclei were detected in the VSMCs of the preglomerular vessels and in interstitial cells of the outer medulla (Fig. 10*B*), as previously observed under NS diet. The inner medulla remained negative for HIF‑2α staining in both groups.

### Low salt/enalapril‐induced EPO expression in preglomerular VSMCs of SMMHC^CreERT2^ PHD2^ff^ PHD3^ff^ mice is reversible

The induction of renin production in preglomerular VSMCs is reversible. Once blood pressure or electrolyte homeostasis has been restored, preglomerular VSMCs lose their endocrine character and revert to their contractile phenotype (Gomez & Lopez, 2017). To investigate, whether EPO induction in PHD2/PHD3‐deficient VSMCs is also reversible, we alternately fed SMMHC^CreERT2^ PHD2^ff^ PHD3^ff^ and control mice with an LSE diet and a normal salt diet in 14‐day cycles, measuring the plasma EPO and renin concentrations at the end of each cycle. Additionally, we monitored plasma EPO and renin concentrations every 14 days in SMMHC^CreERT2^ PHD2^ff^ PHD3^ff^ and control mice that received a normal salt diet continuously.

**Figure 11:**
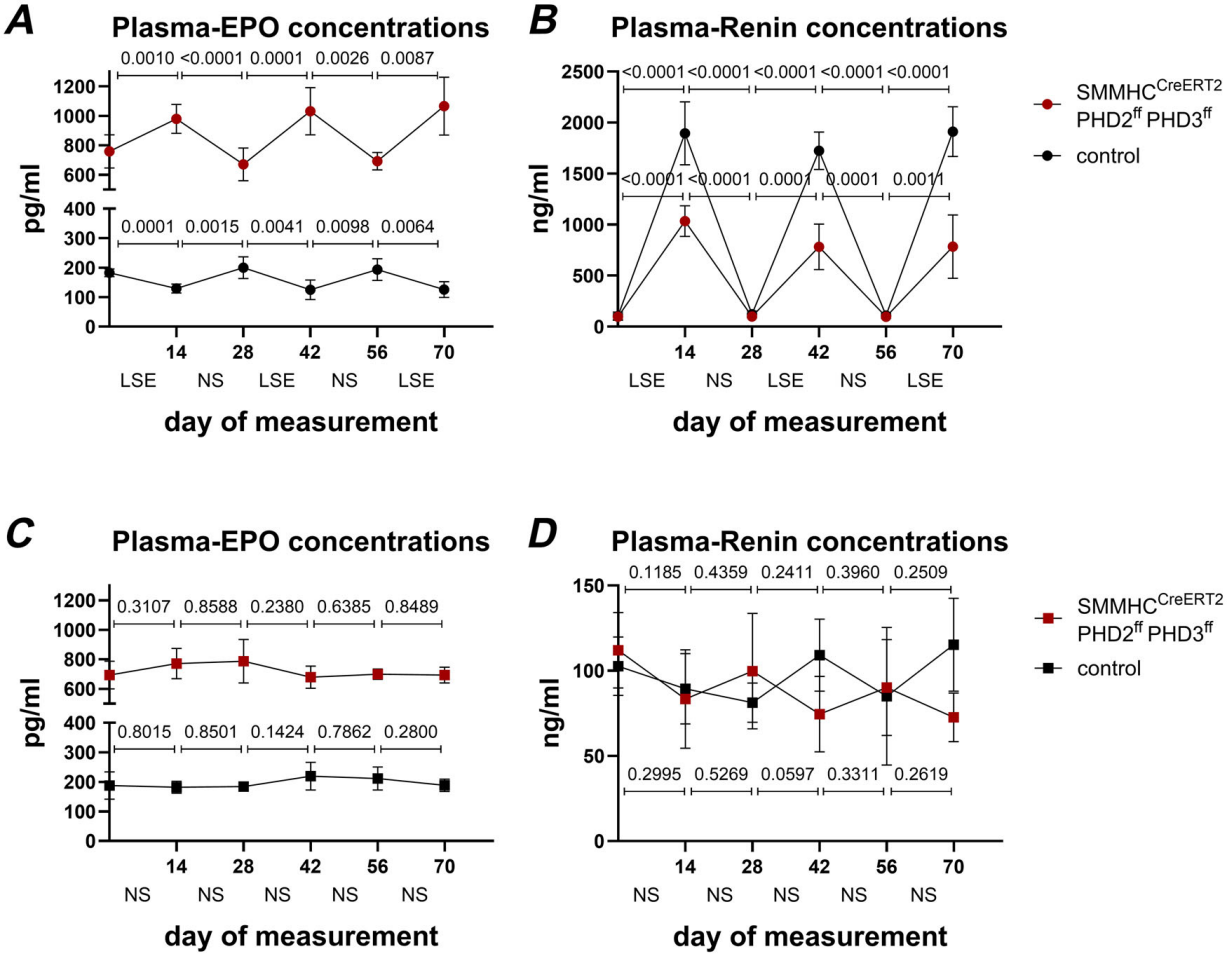
Plasma EPO and renin concentrations in control and SMMHC^CreERT2^ PHD2^ff^ PHD3^ff^ mice that were fed repeated cycles of low salt/enalapril (LSE) and normal salt (NS) diet or that were fed a NS diet continuously Statistical significance between alternating treatments was determined using an unpaired *t* test with Welch's correction, two‐tailed. *P*‐values are stated above the lines. In *D* the upper *P*‐values refer to SMMHC^CreERT2^ PHD2^ff^ PHD3^ff^ mice, the lower *P*‐values refer to the control group. Values are means ± SD of *n* = 5 mice per genotype. *A* and *B*, plasma EPO and renin concentrations of control and SMMHC^CreERT2^ PHD2^ff^ PHD3^ff^ mice measured every 14 days after repeated cycles of 14 days on an LSE diet alternating with 14 days on an NS diet. *C* and *D*, plasma EPO and renin concentrations of control and SMMHC^CreERT2^ PHD2^ff^ PHD3^ff^ mice measured every 14 days while mice were continuously fed a normal salt diet.

The plasma EPO concentrations of control mice on a continuous NS diet were between 180 and 220 pg/ml, whereas the plasma EPO concentrations of PHD2/PHD3‐KO animals remained within the range 680–790 pg/ml (Fig. 11*C*).

In contrast, each LSE cycle resulted in increased plasma EPO concentrations in SMMHC^CreERT2^ PHD2^ff^ PHD3^ff^ mice with mean values between 980 and 1070 pg/ml, which returned to baseline levels of 670–760 pg/ml after the subsequent normal salt diet cycle. In control animals, plasma EPO concentrations decreased during each LSE cycle to mean values between 125 and 130 pg/ml and returned to values between 180 and 200 pg/ml during NS diet, which corresponds well to the baseline level (Fig. 11*A*).

Plasma renin concentrations in control and SMMHC^CreERT2^ PHD2^ff^ PHD3^ff^ mice on a continuous NS diet ranged between 80 and 120 ng/ml (Fig. 11*D*). After each LSE treatment cycle, plasma renin concentrations increased significantly in both groups and returned to baseline values of around 100 ng/ml after the subsequent NS cycle. However, as was previously observed after one 14‐day cycle of LSE treatment, PHD2/PHD3‐KO mice only reached plasma renin concentrations of 780–1000 ng/ml during an LSE cycle, whereas the control group reached values of 1700‐1900 ng/ml (Fig. 11*B*).

**Figure 12:**
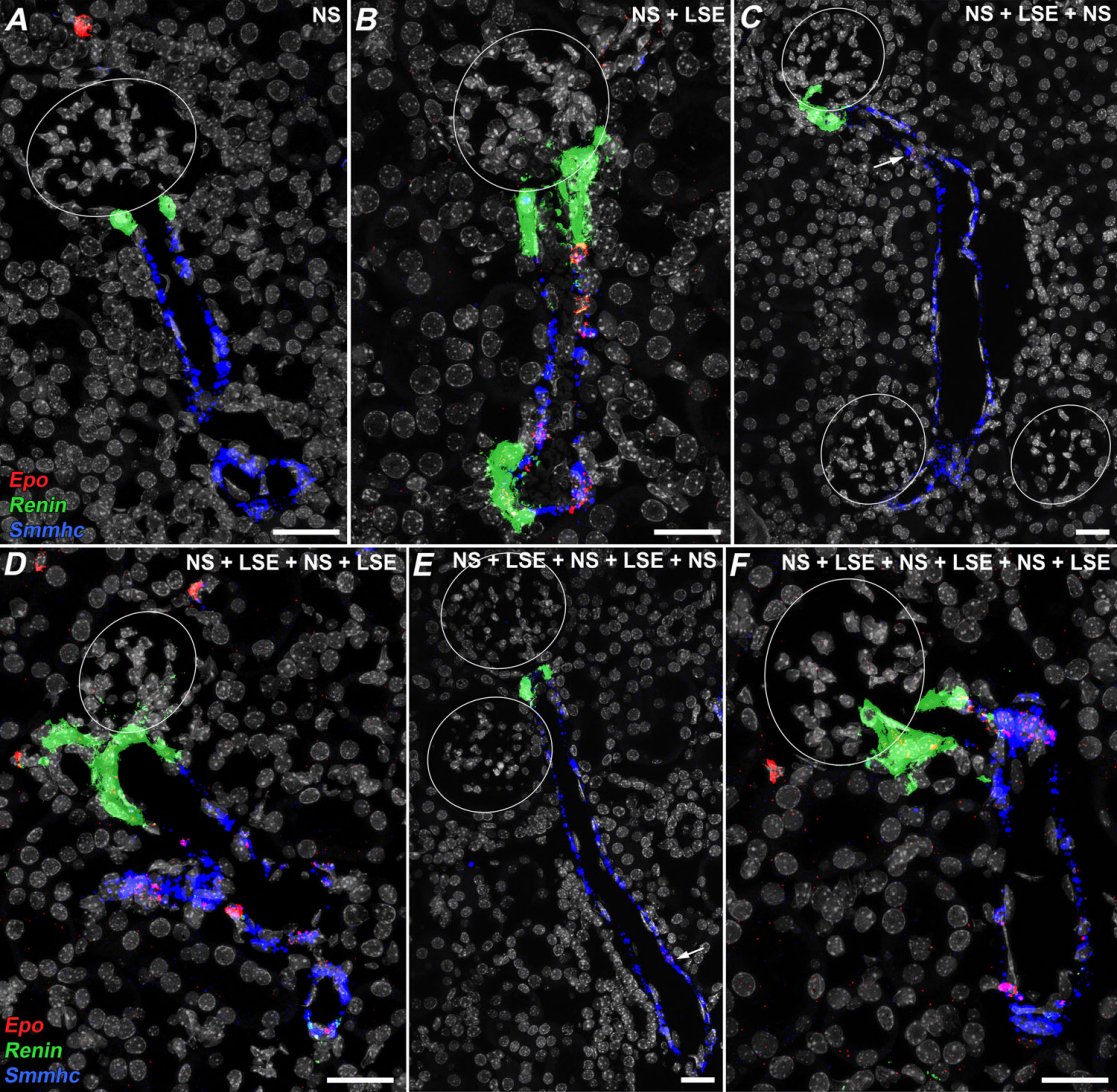
*Renin* and *Epo* mRNA expression in VSMCs of SMMHC^CreERT2^ PHD2^ff^ PHD3^ff^ mice that were fed repeated cycles of normal salt (NS) and low salt/enalapril (LSE) diet Representative details of an RNAscope for *Epo* (red), *Renin* (green) and *Smmhc* (blue) transcripts on kidney sections of PHD2/PHD3‐KO mice after the respective diet cycle stated in the image. The arrows (*C* and *E*) indicate preglomerular VSMCs that still show weak *Epo* positive signals. Circles indicate glomeruli. Nuclei were counterstained with DAPI (grey). Scale bars: 50 µm.

Additionally, *Epo* and *renin* transcripts were analysed on kidney sections after each dietary cycle using RNAscope. While *Epo* positive preglomerular VSMCs were detectable after each LSE cycle, *Epo* expression was no longer observed after the subsequent NS cycle. Sporadically, VSMCs were identified that still showed weak *Epo* positive signals (Fig. 12).

### Short‐term pharmacological PHD inhibition does not induce EPO expression in preglomerular VSMCs or interstitial contractile pericytes

**Figure 13:**
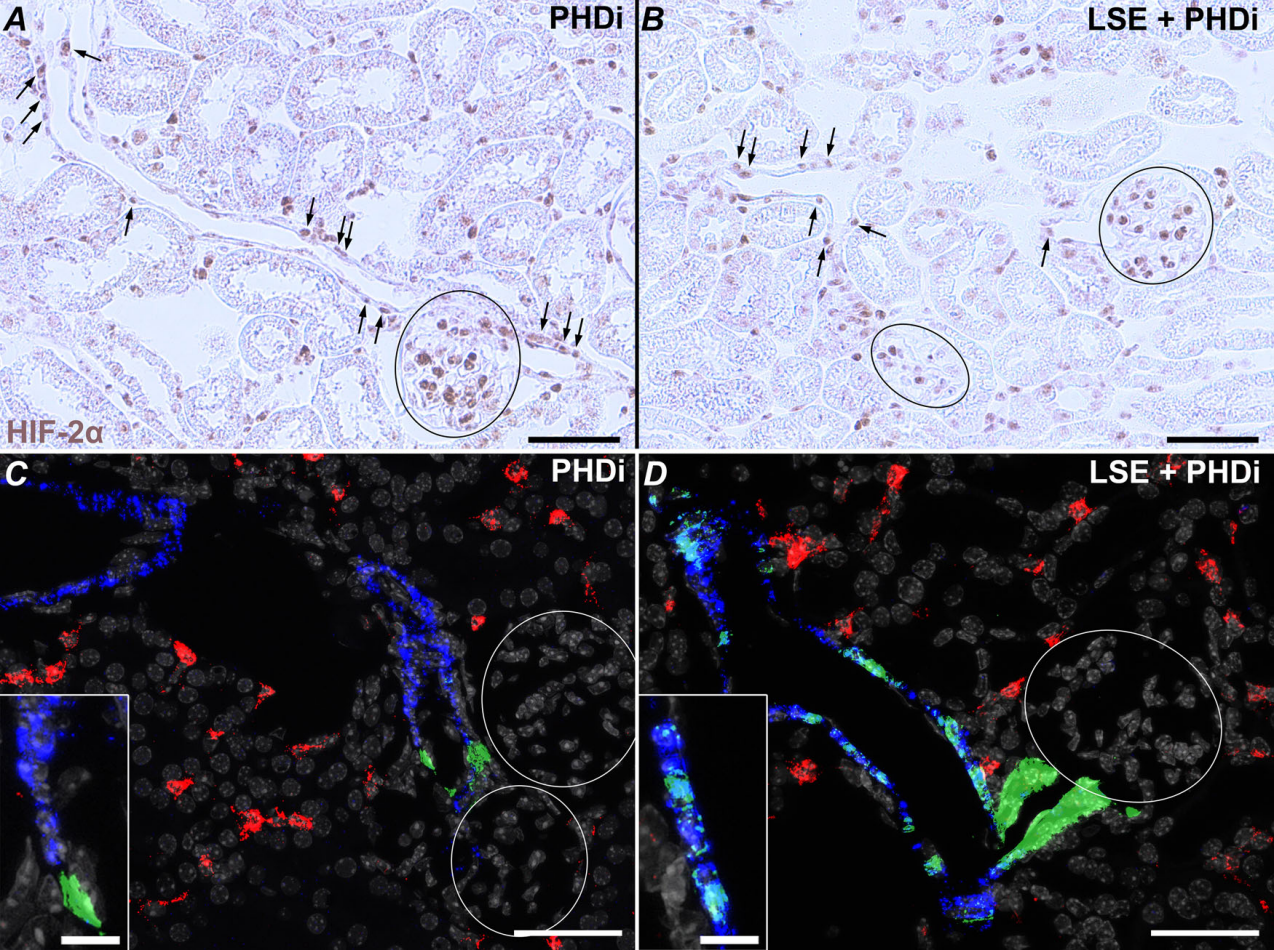
HIF‐2α stabilization and *Epo* mRNA induction on kidney sections of NS‐ or LSE‐fed wild‐type mice treated with the prolyl‐4‐hydroxylase inhibitor (PHDi) roxadustat *A* and *B*, cortical details showing HIF‐2α stabilization by immunohistochemistry on kidney sections of control mice treated with roxadustat or a combination of LSE and the PHDi roxadustat. Clear HIF‐2α positive staining (brown nuclei) was observed in VSMCs (arrows), juxtaglomerular cells, intraglomerular mesangial cells and interstitial fibroblasts under both conditions. Circles indicate glomeruli. Scale bars: 50 µm. *C* and *D*, cortical details showing multiplex RNAscope for *Epo* (red), *Smmhc* (blue) and *Renin* (green) transcripts. Under both conditions, no *Epo* mRNA expression could be detected in preglomerular VSMCs or renin‐producing juxtaglomerular cells. Strong *Epo* mRNA expression was only detectable in interstitial fibroblasts. Insets in the lower left corner show some VSMCs of the preglomerular vessel at a higher magnification. Circles indicate glomeruli. Nuclei were counterstained with DAPI (grey). Scale bars: 50 µm; scale bars of insets: 10 µm.

**Figure 14:**
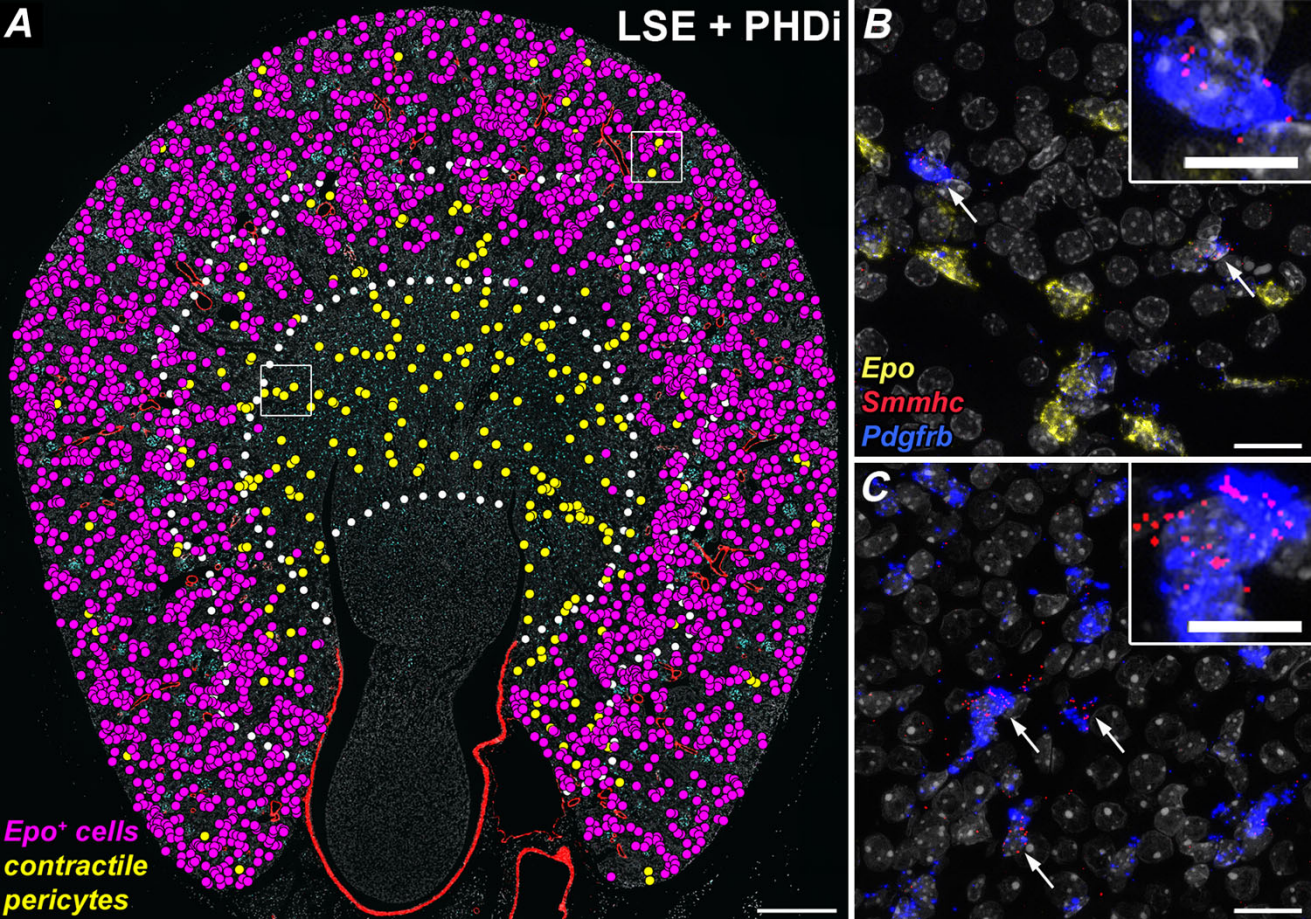
*Epo* mRNA induction in kidneys of wild‐type mice treated with LSE and the PHDi roxadustat *A*, overview of a transverse kidney section showing the distribution of *Epo* expressing cells (pink dots) and interstitial contractile pericytes (yellow dots) detected by RNAscope on kidney sections of wild‐type mice treated with LSE and roxadustat. White dotted lines indicate zonal borders. The square boxes in the cortex and the inner stripe of the outer medulla indicate the areas shown in detail in *B* and *C*, respectively. Nuclei were counterstained with DAPI (grey). Scale bar: 500 µm. *B* and *C*, details showing multiplex RNAscope for *Epo* (yellow), *Smmhc* (red) and *Pdgfrb* (blue) transcripts on kidney sections of wild‐type mice treated with LSE and roxadustat. Arrows highlight interstitial contractile pericytes in the cortex (*B*) or the inner stripe of the outer medulla (*C*). Insets in the upper right corner show a contractile pericyte at a higher magnification. Nuclei were counterstained with DAPI (grey). Scale bars: 20 µm; scale bars of insets: 10 µm.

Many patients with high blood pressure, including those with chronic kidney disease, are prescribed ACE inhibitors such as enalapril. They are also advised to limit their salt intake. Based on this and our findings, we examined whether inactivating PHDs with a pharmacological inhibitor could affect the endocrine product of preglomerular VSMCs. To investigate this, we treated wild‐type mice that were fed either a normal salt diet or an LSE diet for 14 days with the PHD inhibitor roxadustat. Roxadustat was administered eight times at 90‐min intervals to stabilize HIF‐2α continuously for 12 h on the last day of the LSE treatment. Under both conditions, we detected clear HIF‐2α stabilization in VSMCs, juxtaglomerular cells, intraglomerular mesangial cells and interstitial fibroblasts throughout the kidneys (Fig. 13*A* and *B*). However, *Epo* mRNA induction was not detectable in preglomerular VSMCs or juxtaglomerular cells using RNAscope (Fig. 13*C* and *D*). Interstitial *Smmhc^+^* contractile pericytes were also negative for *Epo* (Fig. 14). Strong *Epo* mRNA induction was only observed in interstitial fibroblasts of the cortex and in the outer stripe of the outer medulla under both conditions (Figs 13*C*, *D* and 14). This resulted in plasma EPO concentrations of about 113,000.0 ± 30,012.2 pg/ml in the normal salt group and 109,807.5 ± 31,408.6 pg/ml in the LSE group. Juxtaglomerular renin expression patterns or renin expression patterns in VSMCs and extraglomerular mesangial cells as well as plasma renin concentrations were comparable to those of control mice on an NS or LSE diet (plasma renin concentrations: 131.2 ± 46.8 ng/ml and 1679.4 ± 594.2 ng/ml, respectively).

### PHD2/PHD3 deletion induces a transcriptional switch in preglomerular VSMCs from a contractile/renin cell‐like signature to a contractile/EPO cell‐like signature under normal salt conditions

In a previous study, we demonstrated that renin cell‐specific deletion of PHD2 and PHD3 induces EPO and upregulates EPO cell‐associated markers, while downregulating renin and renin cell markers (Broeker et al., 2021). These observations suggested a metaplastic transformation of renin cells towards a more fibroblast‐like, EPO‐producing cell type. Therefore, we investigated whether a similar transformation occurs in VSMCs following the codeletion of PHD2 and PHD3. To this end, we performed targeted spatial analysis of different cell markers using the RNAscope multiplexing technique. Typical renin cell markers include aldo‐keto‐reductase 1 family b7 (*Akr1b7*) and connexin 40 (*Cx40*). Preglomerular VSMCs typically express *Cx45*, *Smmhc* and regulator of G protein signalling (*Rgs*) 2, 4 and 5 transcripts (Brunskill et al., 2011; Siedlecki et al., 2011), while native EPO‐producing cells are positive for *Cd73* and the HIF‐2α target genes *Rgs4* and *Adm*. The cell marker expression patterns were analysed in native VSMCs (control mice on NS diet), renin^+^ VSMCs (control mice on LSE diet), PHD2/PHD3 deficient VSMCs (SMMHC^CreERT2^ PHD2^ff^ PHD3^ff^ mice on NS diet) and EPO^+^ PHD2/PHD3 deficient VSMCs (SMMHC^CreERT2^ PHD2^ff^ PHD3^ff^ mice on LSE diet).

Indeed, transcriptional changes occurred in PHD2/PHD3‐deficient preglomerular VSMCs not only under LSE‐treatment conditions but also under NS‐conditions.

**Figure 15:**
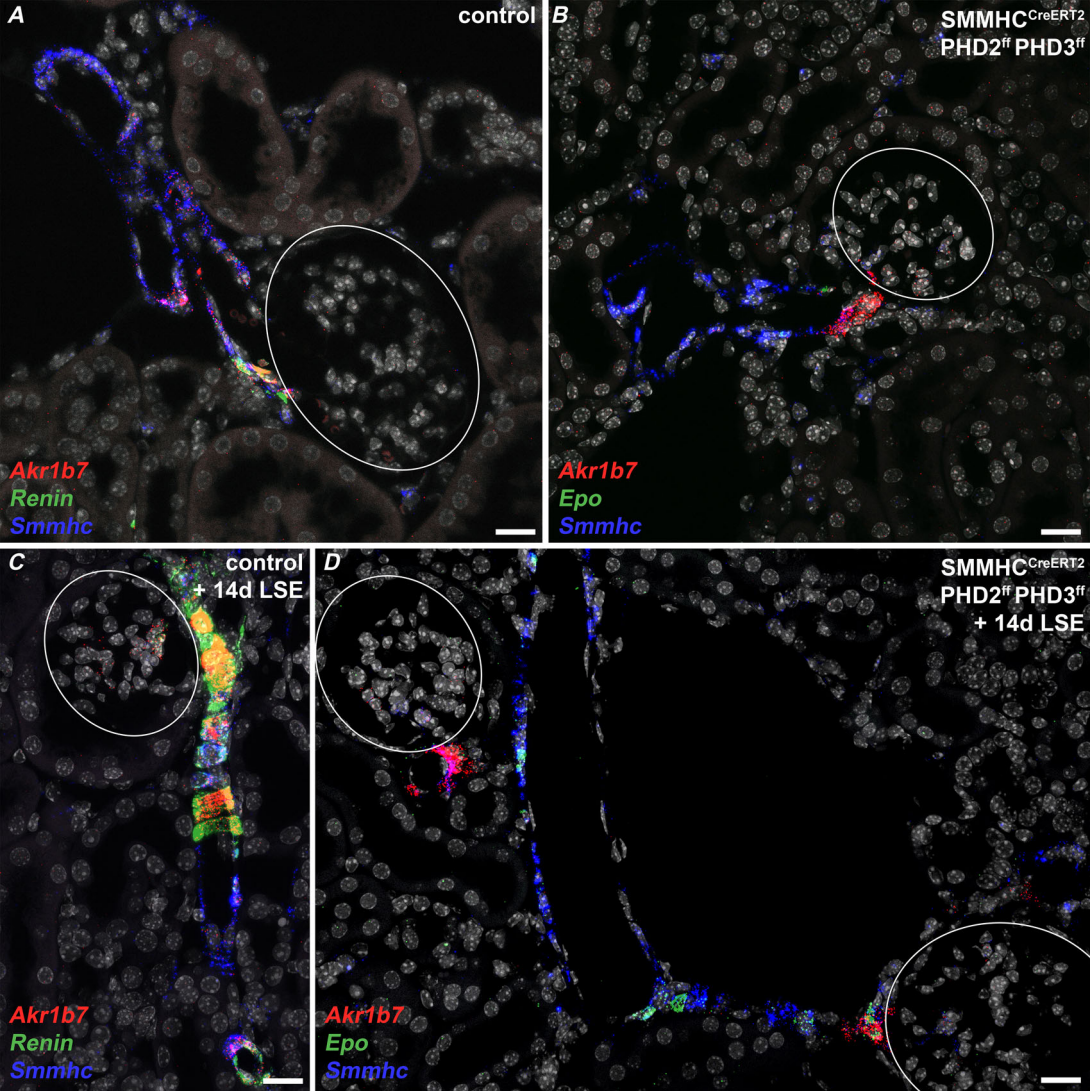
Coexpression of *Renin* or *Epo* with *Akr1b7* mRNA in VSMCs of control and SMMHC^CreERT2^ PHD2^ff^ PHD3^ff^ mice with and without LSE treatment Details of an RNAscope for *Renin* (green) or *Epo* (green), *Akr1b7* (red) and *Smmhc* (blue) transcripts on kidney sections of control or SMMHC^CreERT2^ PHD2^ff^ PHD3^ff^ mice without or with LSE diet. Circles indicate glomeruli. Nuclei were counterstained with DAPI (grey). Scale bars: 50µm. *A* and *C*, in control mice *Akr1b7 s*ignals could be detected in juxtaglomerular renin producing cells as well as in preglomerular VSMCs (*A*). After LSE treatment *Akr1b7* mRNA expression was upregulated together with renin in transformed VSMCs (*C*). *B* and *D*, in SMMHC^CreERT2^ PHD2^ff^ PHD3^ff^ mice *Akr1b7* transcripts could no longer be detected in VSMCs after PHD2/PHD3 deletion, either under basal conditions (*B*) or after LSE treatment (*D*). *Akr1b7* mRNA expression could only be detected at the juxtaglomerular position.

While native renin‐negative VSMCs already expressed the renin‐cell marker *Akr1b7* mRNA, *Akr1b7* mRNA expression increased markedly in transformed *Renin^+^* VSMCs following LSE treatment (Fig. 15*A* and *C*). However, *Akr1b7* mRNA expression was lost in SMMHC^CreERT2^ PHD2^ff^ PHD3^ff^ mice fed a normal salt diet, and it was not re‐induced in (*Epo^+^*) VSMCs after LSE treatment (Fig. 15*B* and *D*). Similar results were obtained for Cx40. Under LSE conditions, *Cx40* mRNA expression was induced together with renin in transformed VSMCs, while native VSMCs were negative for *Cx40* transcripts. In PHD2/PHD3‐deficient VSMCs, *Cx40* mRNA expression was not detected, either with or without an LSE diet (Table 5).

**Table 5:** Expression profile of preglomerular VSMCs under different conditions analysed by RNAscope

Marker	Native VSMCs (control)	Renin^+^ VSMCs (control + LSE)	PHD2/PHD3 deficient VSMCs	EPO^+^ PHD2/PHD3 deficient VSMCs (+ LSE)
*Renin*	−	+	−	−
*Epo*	−	−	−	+
*Akr1b7*	+	++	−	−
*Cx40*	−	+	−	−
*Cx45*	+	+	+	+
*Smmhc*	++	(+)↓	++	++
*Rgs2*	+	−	+	+
*Rgs4*	+	+	+	+
*Rgs5*	++	++	++	++
*Adm*	−	−	+	+
*Cd73*	−	−	+	+
*Creb‐bp*	+	+	+	+

(−) no expression; (+) expression; (++) stronger expression. *Epo*, *erythropoietin*; *Akr1b7*, aldo‐keto‐reductase family 1, member B7; *Cx40*, connexin 40; *Cx45*, connexin 45; Smmhc, smooth muscle‐myosin heavy chain; *Rgs2/Rgs4/Rgs5*, Regulator of G protein signalling 2/4/5; *Adm*, adrenomedullin; *Cd73*, cluster of differentiation 73; *Creb‐bp*, CREB‐binding protein (CBP).

**Figure 16:**
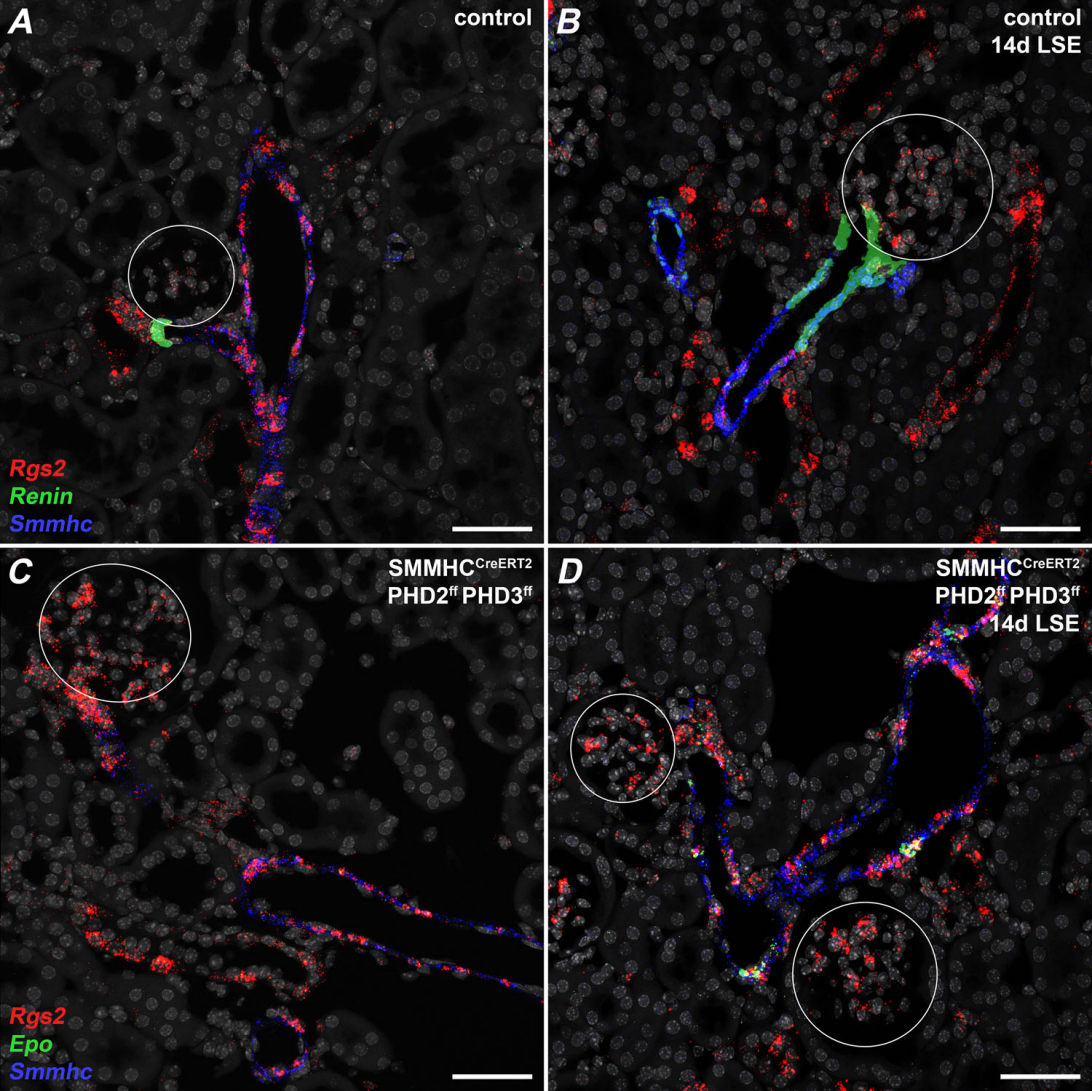
Coexpression of *Renin* or *Epo* with *Rgs2* mRNA in VSMCs of control and SMMHC^CreERT2^ PHD2^ff^ PHD3^ff^ mice with and without LSE treatment Details of an RNAscope for *Renin* (green) or *Epo* (green), *Rgs2* (red) and *Smmhc* (blue) transcripts on kidney sections of control or SMMHC^CreERT2^ PHD2^ff^ PHD3^ff^ mice without or with LSE diet. Circles indicate glomeruli. Nuclei were counterstained with DAPI (grey). Scale bars: 50µm. *A* and *B*, in control mice *Rgs2* mRNA expression could be detected in preglomerular VSMCs. Juxtaglomerular renin producing cells showed only weak *Rgs2 s*ignals (*A*). After LSE treatment *Rgs2* mRNA expression was downregulated in preglomerular (renin‐producing) VSMCs (*B*). *C* and *D*, in SMMHC^CreERT2^ PHD2^ff^ PHD3^ff^ mice, *Rgs2* mRNA expression was not changed compared to control mice without LSE treatment (*C*). (EPO^+^) VSMCs were also positive for *Rgs2* transcripts under LSE conditions (*D*).

The expression of *Cx45* mRNA, the connexin isoform expressed in preglomerular VSMCs of normal‐salt‐fed control mice, did not change in any of the analysed conditions (Table 5). However, when VSMCs are transformed into renin‐producing cells following LSE treatment, the expression of contractile proteins such as *Smmhc* is downregulated. This was not observed when PHD2/PHD3‐deficient preglomerular VSMCs started to produce EPO due to LSE treatment. RNAscope revealed a strong, mosaic‐like *Rgs2* mRNA expression in preglomerular VSMCs of control mice on a normal salt diet, whereas juxtaglomerular renin cells showed only weak *Rgs2 s*ignals (Fig. 16*A*). Following LSE treatment, *Rgs2* mRNA expression was markedly downregulated in renin^+^ VSMCs (Fig. 16*B*). PHD2/PHD3 codeletion did not affect *Rgs2* mRNA expression in preglomerular VSMCs under normal salt conditions and it was no longer downregulated with LSE treatment (Fig. 16*C* and *D*). The expression patterns of *Rgs4* and *Rgs5* did not change in any of the analysed conditions (Table 5).

**Figure 17:**
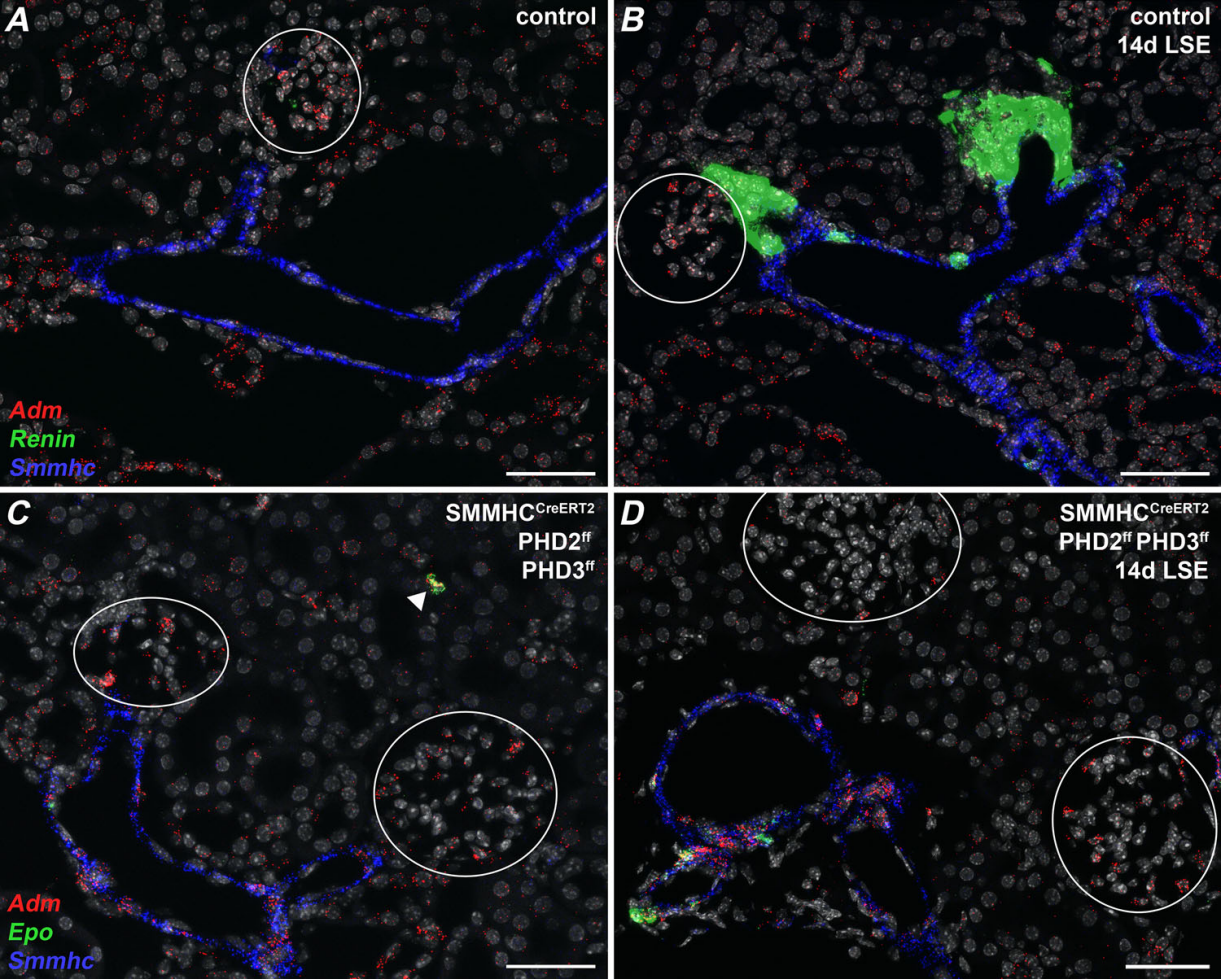
Coexpression of *Renin* or *Epo* with *Adm* mRNA in SMMHC^+^ VSMCs of control and SMMHC^CreERT2^ PHD2^ff^ PHD3^ff^ mice with and without LSE treatment Details of an RNAscope for *Renin* (green) or *Epo* (green), *Adm* (red) and *Smmhc* (blue) transcripts on kidney sections of control or SMMHC^CreERT2^ PHD2^ff^ PHD3^ff^ mice without or with LSE diet. Circles indicate glomeruli. Nuclei were counterstained with DAPI (grey). Scale bars: 50µm. *A* and *B*, *Adm* gene expression could not be detected in preglomerular VSMCs of control mice without (*A*) or with LSE (*B*) diet. *Adm* transcripts were restricted to some tubular and intraglomerular cells under both conditions. *C* and *D*, after SMMHC cell‐specific PHD2/PHD3 deletion, *Adm* mRNA expression could be detected in preglomerular VSMCs (*C*). After LSE treatment, also *Epo*/*Adm* coexpression could be detected in VSMCs (*D*). In addition, interstitial *Epo^+^* cells coexpressed *Adm* (arrowhead; *C*).

Expression of neither the EPO cell marker gene *Cd73* nor the HIF‐2α‐regulated target gene *Adm* could be detected in native or renin‐positive VSMCs in control mice. However, expression of both genes could be detected in VSMCs of SMMHC^CreERT2^ PHD2^ff^ PHD3^ff^ mice, whether or not they were on an LSE diet (Fig. 17 and Table 5).

Table 5 summarizes the changes, which indicate a transcriptional switch in preglomerular VSMCs from a contractile/renin cell‐like signature to a more contractile/EPO cell‐like signature following PHD2/PHD3 deletion.

## Discussion

Given the endocrine plasticity of preglomerular VSMCs and juxtaglomerular renin‐producing cells, and considering our previous finding that the prolyl‐4‐hydroxylases PHD2 and PHD3 control EPO production in renin‐producing cells, this study analysed the significance of PHD2 and PHD3 for the endocrine function and plasticity of preglomerular pericyte‐like VSMCs. In addition, the role of PHD2 and PHD3 in the interstitial contractile pericytes was examined. To this end, PHD2, PHD3 or both were inducibly deleted under the the control of the Smmhc promotor, as SMMHC is a contractile protein expressed in VSMCs and interstitial contractile pericytes. The production of renin and EPO was analysed in the different PHD‐deficient mice, either under basal conditions (normal salt diet) or under renin‐stimulating conditions, by feeding the mice a low salt diet combined with the ACE inhibitor enalapril.

Under basal conditions, deletion of PHD2 and/or PHD3 did not affect the endocrine function of preglomerular VSMCs. No *Epo* induction was detectable in preglomerular VSMCs in any of the PHD‐deficient mouse models examined, and renin production remained intact. In contrast, robust *Epo* mRNA expression was observed in contractile pericytes of the outer medulla in normal‐salt‐fed SMMHC^CreERT2^ PHD2^ff^ and SMMHC^CreERT2^ PHD2^ff^ PHD3^ff^ mice (Fig. 4), with renal *Epo* expression and protein synthesis being significantly higher in the PHD2/PHD3‐KO animals compared with the PHD2‐KO animals (Fig. 2*E* and *H*). Accordingly, SMMHC^CreERT2^ PHD2^ff^ PHD3^ff^ mice displayed a greater number of interstitial *Epo* ^+^ contractile pericytes (Fig. 5). RNAscope analyses revealed that some contractile pericytes expressed only PHD2, while others expressed both PHD2 and PHD3. Given that PHD3 has been shown to increase the threshold for HIF‐2α stabilization (Firmke et al., 2025), the absence of both PHD2 and PHD3 explains the broader induction of EPO production in interstitial pericytes of PHD2/PHD3‐KO mice compared with PHD2‐KO mice. In line with this, the number of HIF‐2α positive interstitial cells in the outer medulla was higher in PHD2/PHD3‐KO mice than in PHD2‐KO mice (Fig. 6). These findings are consistent with a previous study identifying interstitial SMMHC^+^ contractile pericytes as a subpopulation of renal interstitial PDGFR‐β^+^ fibroblasts capable of producing EPO (Broeker et al., 2020).

The absence of EPO induction in preglomerular VSMCs could not be attributed to insufficient HIF‐2α stabilization, at least not in SMMHC^CreERT2^ PHD2^ff^ PHD3^ff^ mice, in which robust HIF‐2α accumulation was detectable (Fig. 6*D*). Moreover, induction of the HIF‐2α target gene *Adm* confirmed active HIF signalling within preglomerular VSMCs of these animals (Fig. 17). Sporadic *Epo* expression was only observed at the juxtaglomerular position in a few glomeruli of SMMHC^CreERT2^ PHD2^ff^ PHD3^ff^ mice (Fig. 3*D*), and *Renin* mRNA expression appeared downregulated in these cells. This aligns with our previous findings showing that, following PHD2/PHD3 codeletion, renin‐producing cells undergo metaplastic transformation into EPO‐producing cells (Broeker et al., 2021). Although SMMHC expression is considerably lower in renin‐producing cells than in non‐renin‐producing VSMCs, the transition between more endocrine and more contractile states of renin cells is known to be dynamic (Brunskill et al., 2011; Gomez & Lopez, 2017). It is therefore plausible that, in some renin cells, SMMHC promoter activity during the 3‐week tamoxifen induction period was sufficient to mediate PHD2 and PHD3 deletion, triggering metaplastic cell transformation. However, because only a small fraction of renin cells were affected, overall R*enin* expression and synthesis remained unchanged between the SMMHC^CreERT2^ PHD2^ff^ PHD3^ff^ mice and their respective controls (Fig. 2).

When we attempted to stimulate renin production in VSMCs by lowering systolic blood pressure through a low‐salt diet combined with the ACE inhibitor enalapril, PHD2/PHD3‐deficient preglomerular VSMCs no longer produced renin but instead began producing EPO. Consistent with previous studies (Neubauer et al., 2013; Pentz et al., 2012), LSE treatment in control mice triggered a strong induction of renin synthesis, accompanied by the characteristic transformation of preglomerular VSMCs and extraglomerular mesangial cells into renin‐producing cells, as confirmed by RNAscope (Figs 7 and 8) (Guessoum et al., 2020; Sequeira López et al., 2004). Similar responses were observed in LSE‐treated PHD2‐ and PHD3‐KO mice. In contrast, LSE‐treated SMMHC^CreERT2^ PHD2^ff^ PHD3^ff^ mice showed no renin induction in the preglomerular VSMCs. Additional *Renin* transcription was confined to extraglomerular mesangial cells, which was reflected in significantly lower renal *Renin* mRNA expression and plasma renin concentrations compared to controls (Figs 7 and 8). A previously reported HIF‐dependent induction of ACE (Yin et al., 2024; Zhang et al., 2009) that could diminish the efficacy of LSE treatment and thereby contribute to the blunted renin response in PHD2/PHD3‐deficient mice was not observed. Although plasma ACE levels were slightly elevated under LSE conditions in all analysed genotypes – as described previously (Fyhrquist et al., 1980; King et al., 1991) – they did not differ between LSE‐treated PHD2/PHD3‐KO mice and the respective controls. Moreover, blood pressure measurements confirmed that systolic pressure in SMMHC^CreERT2^ PHD2^ff^ PHD3^ff^ mice decreased by approximately 40 mmHg, reaching levels comparable to LSE‐treated controls (Table 4).

Instead of transforming into renin‐producing cells, RNAscope analyses indicated that preglomerular VSMCs transformed into EPO‐producing cells due to the LSE treatment. This was supported by the significant increase in renal *Epo* mRNA expression, elevated renal EPO protein, and higher plasma EPO concentrations compared with PHD2/PHD3‐KO mice on a normal‐salt diet. The unchanged number of interstitial EPO‐producing pericytes between LSE‐ and NS‐fed PHD2/PHD3‐KO mice further confirmed that the additional EPO originated from VSMCs (Figs 7 and 9). Moreover, the localization of the detected *Epo^+^* cells corresponded to the sites of HIF‐2α stabilization – along preglomerular VSMCs and in the interstitium of the outer medulla, although the intensity of the HIF‐2α immunostaining did not differ from that observed under NS conditions (Fig. 10).

The significant reduction in renal EPO synthesis observed in control animals following LSE treatment (Fig. 7) aligns with previous reports describing decreased renal *Epo* mRNA expression and lowered haematocrit after administration of angiotensin II type‐1 receptor antagonists or ACE inhibitors in rodents and humans. Increased renal cortical blood flow resulting from reduced angiotensin II‐mediated vasoconstriction is thought to enhance tissue oxygen availability, thereby suppressing EPO production (Rodrigues & Bader, 2023). Consistent with this, LSE treatment did not induce HIF‐2α stabilization in the kidneys of control mice. On the contrary, even fewer interstitial HIF‐2α positive cells were detectable in the corticomedullary region under LSE compared with NS conditions.

Notably, LSE‐induced transformation of PHD2/PHD3‐deficient VSMCs into EPO‐producing cells was reversible, despite the continuous HIF‐2α stabilization, analogous to the reversible recruitment of VSMCs into renin‐producing cells in control animals. After each LSE cycle, when PHD2/PHD3‐KO animals were switched back to a normal salt diet, plasma EPO concentrations returned to baseline levels that had been observed under NS conditions, in which only interstitial pericytes produced EPO. Upon renewed LSE administration, plasma EPO levels rose again, mirroring the cyclic modulation of plasma renin concentrations observed in control animals (Figs 11 and 12). Reversible renin stimulation still occurred in SMMHC^CreERT2^ PHD2^ff^ PHD3^ff^ animals during alternating LSE and NS cycles. However, as renin induction was confined to the extraglomerular mesangium, mean plasma renin levels reached significantly lower values under LSE compared with those of control mice.

These findings may explain an observation made by Kurt et al. (2015) in mice with a renin cell‐specific deletion of the von Hippel–Lindau protein (pVHL) that resulted in the transformation of juxtaglomerular renin‐producing cells into EPO‐producing cells under normal physiological (normal salt) conditions already during kidney development. Kurt et al. reported that plasma EPO concentrations in these mice increased even further following LSE treatment – an unexpected result given that the juxtaglomerular cells were already permanently converted into EPO‐producing cells under normal‐salt conditions. They interpreted this finding as indicating that pVHL‐deficient juxtaglomerular cells might retain some responsiveness to renin‐inducing stimuli (Kurt et al., 2015). However, in their model, pVHL deletion – and the resulting chronic HIF‐2α stabilization – occurred during renal development and therefore affected all renin lineage cells, including preglomerular VSMCs. In light of our results, it is more likely that the additional increase in EPO production in response to LSE treatment was driven by the transformation of preglomerular VSMCs into EPO‐producing cells, even though *Epo* mRNA expression had been restricted to the juxtaglomerular region under NS conditions. This interpretation is consistent with our finding that preglomerular VSMCs do not express EPO under NS conditions despite HIF‐2α stabilization.

Based on these findings, one may hypothesize that PHD2 and PHD3 determine the endocrine output of VSMCs by regulating HIF‐2α stabilization, without impairing their ability to reversibly assume endocrine functions. In line with this concept, targeted spatial transcript analysis indicated that HIF‐2α stabilization caused by PHD2/PHD3 deletion shifted the transcriptional profile of preglomerular VSMCs under NS conditions from a more contractile/renin cell‐like signature toward a more contractile/EPO cell‐like signature, without inducing EPO production.

For example, PHD2/PHD3‐deficient VSMCs no longer expressed *Akr1b7* mRNA, a characteristic marker of the renin cell lineage (Brunskill et al., 2011), but instead upregulated *Cd73* and *Adm*, markers of native EPO‐producing fibroblasts (Bachmann et al., 1993; Broeker et al., 2020; Maxwell et al., 1993). These observations align with our previous work demonstrating that renin‐producing cells undergo metaplastic conversion to EPO‐producing cells following PHD2/PHD3 codeletion (Broeker et al., 2021). Furthermore, hallmark transcriptional responses to LSE treatment in controls, such as co‐induction of *Cx40* and renin mRNA, or downregulation of *Smmhc* and *Rgs2*, were absent in VSMCs of LSE‐treated SMMHC^CreERT2^ PHD2^ff^ PHD3^ff^ mice. Instead, LSE triggered *Epo* transcription in these cells.

Expression of genes implicated in enabling endocrine transformation of VSMCs, including *Cx45* and the transcriptional cofactor *Creb‐bp* (CBP, CREB‐binding protein) did not differ between the analysed genotypes or treatment conditions (Pentz et al., 2012; Schweda et al., 2009).

Why, then, do preglomerular pericyte‐like VSMCs fail to produce EPO under NS conditions despite shifting toward an EPO cell‐like transcriptional pattern after PHD2/PHD3 deletion, while SMMHC^+^ interstitial pericytes readily initiate EPO synthesis? One possibility is that the microenvironment is decisive. PHD2/PHD3‐deficient VSMCs produced EPO only when systolic blood pressure dropped. In contrast, interstitial pericytes reside permanently in the low‐blood‐flow environment of the outer medulla along the vasa recta (Mattson, 2003). Therefore, interstitial pericytes may be continuously primed for endocrine activity, whereas pericyte‐like preglomerular VSMCs may require an additional physiological trigger to adopt an endocrine phenotype.

However, our data also show that short‐term HIF‐2α stabilization alone is insufficient to induce EPO production in preglomerular VSMCs or *Smmhc* ^+^ interstitial pericytes. Treatment of wild‐type mice with the PHD inhibitor roxadustat for 12 h did not trigger *Epo* mRNA expression in these cells under either normal‐salt or LSE conditions, despite clear HIF‐2α stabilization in VSMCs, interstitial fibroblasts/pericytes, juxtaglomerular renin cells and intraglomerular mesangial cells (Figs 13 and 14). Robust *Epo* transcription was observed only in interstitial fibroblasts of the cortex and the outer stripe of the outer medulla. These findings are consistent with our previous study showing that HIF‐2α stabilization alone is not sufficient to induce EPO production in deeper medullary fibroblasts, including contractile pericytes (Firmke et al., 2025).

Taken together, these findings suggest that two conditions must be met to induce EPO production in preglomerular VSMCs: long‐term HIF‐2α stabilization, which enables a shift toward an EPO cell‐like transcriptional signature, and a physiological stimulus such as reduced blood pressure, which permits the acquisition of endocrine function. Because short‐term HIF‐2α stabilization alone failed to induce EPO synthesis, it is plausible that prolonged HIF‐2α activation triggers epigenetic modifications that drive the transcriptional transition from a more contractile/renin cell‐like state to a more contractile/EPO cell‐like state. Indeed, epigenetic regulatory mechanisms influence both *Renin* and *Epo* mRNA expression (Chang et al., 2016; Smith et al., 2024), and HIF‐2α has been shown to affect epigenetic modifications such as histone modifications and DNA methylation (Nangaku et al., 2015). Future studies will therefore focus on identifying the molecular mechanisms that mediate this epigenetic reprogramming.

Moreover, long‐term studies examining the impact of PHD inhibitors (PHDi) on preglomerular VSMCs will be important, as many patients with chronic kidney disease receive ACE inhibitors such as enalapril and are advised to follow a low‐salt diet to control blood pressure. In addition, PHD inhibitors such as roxadustat are approved for the treatment of renal anaemia in patients with chronic kidney disease (CKD). It is important to note that, in the present study, the effects of PHD inhibition were assessed only in healthy kidneys and only after 12 h of treatment. Likewise, many studies investigating PHDi effects on *Epo* mRNA induction in mouse models of CKD commonly administer a single PHDi dose and analyse kidneys approximately 4 h later (Dahl, Pfundstein et al., 2022; Fuchs et al., 2021; Kobayashi et al., 2022). Since treatment with PHDi can cause hypertension in some patients and HIF stabilization across various organs may influence ACE production, such effects on blood pressure must be considered in future long‐term studies investigating renal endocrine VSMC plasticity (Yin et al., 2024; Zhang et al., 2009, 2024).

Nonetheless, we cannot exclude that repeated use of PHDi over a longer period of time could transform preglomerular VSMCs from contractile/renin‐like cells into more contractile/EPO‐like cells, similar to what we observed following genetic PHD2/PHD3 deletion. This effect may be further amplified by use of PHDi with longer half‐lives, such as desidustat, which likely produce more sustained HIF‐2α stabilization with each administration (Li & Ramli, 2025). Furthermore, it remains possible that preglomerular VSMCs or pericytes in CKD patients, who often receive LSE treatment and may exhibit interstitial fibrosis and thus reduced renal oxygen availability, become increasingly susceptible to PHDi‐induced EPO production over time, particularly as damaged kidneys contain fewer cortical fibroblasts capable of responding to PHD inhibition compared with healthy kidneys (Dahl, Pfundstein et al., 2022; Fuchs et al., 2021; Kobayashi et al., 2022).

In summary, this study demonstrates that the HIF‐regulating enzymes PHD2 and PHD3 play a central role in the endocrine function of preglomerular pericyte‐like VSMCs and interstitial contractile pericytes. Chronic HIF‐2α stabilization, mediated by SMMHC cell‐specific deletion of PHD2 or PHD2/PHD3, induced robust EPO production in interstitial contractile pericytes. In preglomerular VSMCs, prolonged HIF‐2α stabilization triggered a transcriptional shift from a contractile/renin cell‐like to a more contractile/EPO cell‐like signature. When these PHD2/PHD3‐deficient VSMCs were subsequently exposed to a physiological stimulus that normally transforms them into renin‐producing cells, they instead transformed reversibly in EPO‐producing cells.

These findings refine our understanding of reversible renin‐cell transformation by suggesting that it comprises two distinct processes: (i) a reversible transition of VSMCs from a primarily contractile state to a more endocrine state that enables synthesis of endocrine factors, and (ii) the production of the specific endocrine factor, which is dictated by the cell's current endocrine signature (renin‐like or EPO‐like).

Moreover, our results indicate that, beyond the known PDGFR‐β^+^ interstitial cell subpopulations capable of producing EPO – including contractile pericytes – additional, previously overlooked cell types such as preglomerular VSMCs also possess the potential to synthesize EPO. Elucidating the precise (including epigenetic) mechanisms governing the switch between renin and EPO production may reveal new insights into the regulation of both EPO and renin and identify therapeutic targets for selectively enhancing or suppressing these endocrine factors.

Such insights may be clinically relevant for CKD, where progressive loss of EPO‐competent interstitial fibroblasts limits the efficacy of PHD inhibitors. In light of our findings, future studies should therefore evaluate the long‐term effects of PHD inhibitor treatment on preglomerular VSMCs and contractile pericytes.

## Additional information

## Competing interests

None declared.

## Author contributions

K.A.E.B. conceived and designed the research studies, analysed and interpreted data and wrote the manuscript. K.A.E.B. and B.K.M.F. made the figures. K.A.E.B., B.K.M.F., L.M.S. and A.L.F. performed experiments and acquired and analysed data. B.K.M.F., A.L.F. and A.K. reviewed the manuscript. A.K. interpreted data and was regularly happy to discuss the data. All authors edited the manuscript. All authors have read and approved the final version of this manuscript and agree to be accountable for all aspects of the work in ensuring that questions related to the accuracy or integrity of any part of the work are appropriately investigated and resolved. All persons designated as authors qualify for authorship, and all those who qualify for authorship are listed.

## Funding

This work was funded by the Deutsche Forschungsgemeinschaft (DFG, German Research Foundation), project number 509149993, TRR 374, Project B1. Image analysis was funded by the Deutsche Forschungsgemeinschaft (DFG, German Research Foundation) – Projektnummer 471535567.

## Supporting information


Peer Review History

## Data Availability

All data supporting our results are included in the manuscript. Original data and micrographs are available on request. Please contact Dr Katharina Broeker (katharina.broeker@ur.de).

## References

[tjp70572-bib-0001] Bachmann, S. , Le Hir, M. , & Eckardt, K. U. (1993). Co‐localization of erythropoietin mRNA and ecto‐5’‐nucleotidase immunoreactivity in peritubular cells of rat renal cortex indicates that fibroblasts produce erythropoietin. Journal of Histochemistry and Cytochemistry, 41(3), 335–341. 8429197 10.1177/41.3.8429197

[tjp70572-bib-0002] Broeker, K. A. E. , Fuchs, M. A. A. , Schrankl, J. , Kurt, B. , Nolan, K. A. , Wenger, R. H. , Kramann, R. , Wagner, C. , & Kurtz, A. (2020). Different subpopulations of kidney interstitial cells produce erythropoietin and factors supporting tissue oxygenation in response to hypoxia in vivo. Kidney International, 98(4), 918–931. 32454122 10.1016/j.kint.2020.04.040

[tjp70572-bib-0003] Broeker, K. A. E. , Fuchs, M. A. A. , Schrankl, J. , Lehrmann, C. , Schley, G. , Todorov, V. T. , Hugo, C. , Wagner, C. , & Kurtz, A. (2021). Prolyl‐4‐hydroxylases 2 and 3 control erythropoietin production in renin expressing cells of mouse kidneys. The Journal of Physiology, 600(3), 671–694. 34863041 10.1113/JP282615

[tjp70572-bib-0004] Brunskill, E. W. , Sequeira‐Lopez, M. L. S. , Pentz, E. S. , Lin, E. , Yu, J. , Aronow, B. J. , Potter, S. S. , & Gomez, R. A. (2011). Genes that confer the identity of the renin cell. Journal of the American Society of Nephrology, 22(12), 2213–2225. 22034642 10.1681/ASN.2011040401 PMC3279933

[tjp70572-bib-0005] Chang, Y.‐T. , Yang, C.‐C. , Pan, S.‐Y. , Chou, Y.‐H. , Chang, F.‐C. , Lai, C.‐F. , Tsai, M.‐H. , Hsu, H.‐L. , Lin, C.‐H. , Chiang, W.‐C. , Wu, M.‐S. , Chu, T.‐S. , Chen, Y.‐M. , & Lin, S.‐L. (2016). DNA methyltransferase inhibition restores erythropoietin production in fibrotic murine kidneys. Journal of Clinical Investigation, 126(2), 721–731. 26731474 10.1172/JCI82819 PMC4731189

[tjp70572-bib-0006] Chomczynski, P. , & Sacchi, N. (1987). Single‐step method of RNA isolation by acid guanidinium thiocyanate‐phenol‐chloroform extraction. Analytical Biochemistry, 162(1), 156–159. 2440339 10.1006/abio.1987.9999

[tjp70572-bib-0007] Dahl, S. L. , Bapst, A. M. , Khodo, S. N. , Scholz, C. C. , & Wenger, R. H. (2022). Fount, fate, features, and function of renal erythropoietin‐producing cells. Pflügers Archiv – European Journal of Physiology, 474(8), 783–797. 35750861 10.1007/s00424-022-02714-7 PMC9338912

[tjp70572-bib-0008] Dahl, S. L. , Pfundstein, S. , Hunkeler, R. , Dong, X. , Knöpfel, T. , Spielmann, P. , Scholz, C. C. , Nolan, K. A. , & Wenger, R. H. (2022). Fate‐mapping of erythropoietin‐producing cells in mouse models of hypoxaemia and renal tissue remodelling reveals repeated recruitment and persistent functionality. Acta Physiology, 234(3), e13768. 10.1111/apha.13768 PMC9286872 34982511

[tjp70572-bib-0009] Eckardt, K.‐U. , Koury, S. T. , Tan, C. C. , Schuster, S. J. , Kaissling, B. , Ratcliffe, P. J. , & Kurtz, A. (1993). Distribution of erythropoietin producing cells in rat kidneys during hypoxic hypoxia. Kidney International, 43(4), 815–823. 8479117 10.1038/ki.1993.115

[tjp70572-bib-0010] Firmke, B. K. M. , Fuchs, M. A. A. , Süß, L. M. , Forst, A.‐L. , Kurtz, A. , & Broeker, K. A.‐E. (2025). Hypoxia‐inducible factor‐2 stabilization is not sufficient to induce erythropoietin production in deeper medullary fibroblasts. The Journal of Physiology, 603(19), 5777–5804. 40853869 10.1113/JP288798 PMC12487596

[tjp70572-bib-0011] Franke, K. , Kalucka, J. , Mamlouk, S. , Singh, R. P. , Muschter, A. , Weidemann, A. , Iyengar, V. , Jahn, S. , Wieczorek, K. , Geiger, K. , Muders, M. , Sykes, A. M. , Poitz, D. , Ripich, T. , Otto, T. , Bergmann, S. , Breier, G. , Baretton, G. , Fong, G.‐H. , … Wielockx, B. (2013). HIF‐1α is a protective factor in conditional PHD2‐deficient mice suffering from severe HIF‐2α‐induced excessive erythropoiesis. Blood, 121(8), 1436–1445. 23264599 10.1182/blood-2012-08-449181 PMC3628111

[tjp70572-bib-0012] Fuchs, M. A. A. , Broeker, K. A. E. , Schrankl, J. , Burzlaff, N. , Willam, C. , Wagner, C. , & Kurtz, A. (2021). Inhibition of transforming growth factor β1 signaling in resident interstitial cells attenuates profibrotic gene expression and preserves erythropoietin production during experimental kidney fibrosis in mice. Kidney International, 10(1), 122–137. 10.1016/j.kint.2021.02.035 33705825

[tjp70572-bib-0013] Fyhrquist, F. , Forslund, T. , Tikkanen, I. , & Grönhagen‐Riska, C. (1980). Induction of angiotensin I‐converting enzyme in rat lung with captopril (SQ 14225). European Journal of Pharmacology, 67(4), 473–475. 6256177 10.1016/0014-2999(80)90189-2

[tjp70572-bib-0014] Gerl, K. , Miquerol, L. , Todorov, V. T. , Hugo, C. P. M. , Adams, R. H. , Kurtz, A. , & Kurt, B. (2015). Inducible glomerular erythropoietin production in the adult kidney. Kidney International, 88(6), 1345–1355. 26398496 10.1038/ki.2015.274

[tjp70572-bib-0015] Gerl, K. , Nolan, K. A. , Karger, C. , Fuchs, M. , Wenger, R. H. , Stolt, C. C. , Willam, C. , Kurtz, A. , & Kurt, B. (2016). Erythropoietin production by PDGFR‐β+ cells. Pflügers Archiv – European Journal of Physiology, 468(8), 1479–1487. 27220347 10.1007/s00424-016-1829-2

[tjp70572-bib-0016] Gerl, K. , Steppan, D. , Fuchs, M. , Wagner, C. , Willam, C. , Kurtz, A. , & Kurt, B. (2017). Activation of hypoxia signaling in stromal progenitors impairs kidney development. American Journal of Pathology, 187(7), 1496–1511. 28527294 10.1016/j.ajpath.2017.03.014

[tjp70572-bib-0017] Gomez, R. A. , & Lopez, M. (2017). Plasticity of renin cells in the kidney vasculature. Current Hypertension Reports, 19(2), 14. 28233238 10.1007/s11906-017-0711-8 PMC5734911

[tjp70572-bib-0018] Grundy, D. (2015). Principles and standards for reporting animal experiments in The Journal of Physiology and Experimental Physiology. The Journal of Physiology, 593(12), 2547–2549. 26095019 10.1113/JP270818 PMC4500341

[tjp70572-bib-0019] Guessoum, O. , Zainab, M. , Sequeira‐Lopez, M. L. S. , & Gomez, R. A. (2020). Proliferation does not contribute to murine models of renin cell recruitment. Acta Physiologica, 230(3), e13532.

[tjp70572-bib-0020] Haase, V. H. (2013). Regulation of erythropoiesis by hypoxia‐inducible factors. Blood Reviews, 27(1), 41–53. 23291219 10.1016/j.blre.2012.12.003 PMC3731139

[tjp70572-bib-0021] Humphreys, B. D. , Lin, S.‐L. , Kobayashi, A. , Hudson, T. E. , Nowlin, B. T. , Bonventre, J. V. , Valerius, M. T. , McMahon, A. P. , & Duffield, J. S. (2010). Fate tracing reveals the pericyte and not epithelial origin of myofibroblasts in kidney fibrosis. American Journal of Pathology, 176(1), 85–97. 20008127 10.2353/ajpath.2010.090517 PMC2797872

[tjp70572-bib-0022] King, S. J. , Oparil, S. , & Berecek, K. H. (1991). Neuronal angiotensin‐converting enzyme (ACE) gene expression is increased by converting enzyme inhibitors (CEI). Molecular and Cellular Neuroscience, 2(1), 13–20. 19912779 10.1016/1044-7431(91)90035-m

[tjp70572-bib-0023] Kobayashi, H. , Davidoff, O. , Pujari‐Palmer, S. , Drevin, M. , & Haase, V. H. (2022). EPO synthesis induced by HIF‐PHD inhibition is dependent on myofibroblast transdifferentiation and colocalizes with non‐injured nephron segments in murine kidney fibrosis. Acta Physiol, 235(4), e13826. 10.1111/apha.13826 PMC9329237 35491502

[tjp70572-bib-0024] Kobayashi, H. , Liu, Q. , Binns, T. C. , Urrutia, A. A. , Davidoff, O. , Kapitsinou, P. P. , Pfaff, A. S. , Olauson, H. , Wernerson, A. , Fogo, A. B. , Fong, G.‐H. , Gross, K. W. , & Haase, V. H. (2016). Distinct subpopulations of FOXD1 stroma‐derived cells regulate renal erythropoietin. Journal of Clinical Investigation, 126(5), 1926–1938. 27088801 10.1172/JCI83551 PMC4855934

[tjp70572-bib-0025] Koivunen, P. , & Kietzmann, T. (2018). Hypoxia‐inducible factor prolyl 4‐hydroxylases and metabolism. Trends in Molecular Medicine, 24(12), 1021–1035. 30391126 10.1016/j.molmed.2018.10.004

[tjp70572-bib-0026] Kurt, B. , Gerl, K. , Karger, C. , Schwarzensteiner, I. , & Kurtz, A. (2015). Chronic hypoxia‐inducible transcription factor‐2 activation stably transforms juxtaglomerular renin cells into fibroblast‐like cells in vivo. Journal of the American Society of Nephrology, 2(3), 587–596. 10.1681/ASN.2013111152 PMC4341471 25071089

[tjp70572-bib-0027] Li, Q. , & Ramli, N. N. N. (2025). Hypoxia‐inducible factor prolyl hydroxylase (HIF‐PHD) inhibitors: A therapeutic double‐edged sword in immunity and inflammation. Journal of Molecular Pathology, 6(4), 25.

[tjp70572-bib-0028] Mattson, D. L. (2003). Importance of the renal medullary circulation in the control of sodium excretion and blood pressure. American Journal of Physiology‐Regulatory, Integrative and Comparative Physiology, 284(1), R13–R27. 12482743 10.1152/ajpregu.00321.2002

[tjp70572-bib-0029] Maxwell, P. H. , Osmond, M. K. , Pugh, C. W. , Heryet, A. , Nicholls, L. G. , Tan, C. C. , Doe, B. G. , Ferguson, D. J. , Johnson, M. H. , & Ratcliffe, P. J. (1993). Identification of the renal erythropoietin‐producing cells using transgenic mice. Kidney International, 44(5), 1149–1162. 8264149 10.1038/ki.1993.362

[tjp70572-bib-0030] Minamishima, Y. A. , Moslehi, J. , Bardeesy, N. , Cullen, D. , Bronson, R. T. , & Kaelin, W. G. (2008). Somatic inactivation of the PHD2 prolyl hydroxylase causes polycythemia and congestive heart failure. Blood, 111(6), 3236–3244. 18096761 10.1182/blood-2007-10-117812 PMC2265460

[tjp70572-bib-0031] Miyauchi, K. , Nakai, T. , Saito, S. , Yamamoto, T. , Sato, K. , Kato, K. , Nezu, M. , Miyazaki, M. , Ito, S. , Tamamoto, M. , & Suzuki, N. (2021). Renal interstitial fibroblasts coproduce erythropoietin and renin under anaemic conditions. EBioMedicine, 64, 103209. 33508746 10.1016/j.ebiom.2021.103209 PMC7841315

[tjp70572-bib-0032] Nangaku, M. , Inagi, R. , Mimura, I. , & Tanaka, T. (2015). Epigenetic changes induced by hypoxia‐inducible factor: A long way still to go as a target for therapy? Journal of the American Society of Nephrology, 26(7), 1478–1480. 25587069 10.1681/ASN.2014121161 PMC4483599

[tjp70572-bib-0033] Neubauer, B. , Machura, K. , Kettl, R. , Lopez, M. , Friebe, A. , & Kurtz, A. (2013). Endothelium‐derived nitric oxide supports renin cell recruitment through the nitric oxide–Sensitive guanylate cyclase pathway. Hypertension, 6(2), 400–407. 10.1161/HYPERTENSIONAHA.111.00221 PMC4037755 23297374

[tjp70572-bib-0034] Paliege, A. , Rosenberger, C. , Bondke, A. , Sciesielski, L. , Shina, A. , Heyman, S. N. , Flippin, L. A. , Arend, M. , Klaus, S. J. , & Bachmann, S. (2010). Hypoxia‐inducible factor‐2alpha‐expressing interstitial fibroblasts are the only renal cells that express erythropoietin under hypoxia‐inducible factor stabilization. Kidney International, 77(4), 312–318. 20016470 10.1038/ki.2009.460

[tjp70572-bib-0035] Pentz, E. S. , Cordaillat, M. , Carretero, O. A. , Tucker, A. E. , Sequeira Lopez, M. L. , & Gomez, R. A. (2012). Histone acetyl transferases CBP and p300 are necessary for maintenance of renin cell identity and transformation of smooth muscle cells to the renin phenotype. American Journal of Physiology‐Heart and Circulatory Physiology, 302(12), H2545–H2552. 22523253 10.1152/ajpheart.00782.2011 PMC3378259

[tjp70572-bib-0036] Rodrigues, A. F. , & Bader, M. (2023). The contribution of the AT1 receptor to erythropoiesis. Biochemical Pharmacology, 217, 115805. 37714274 10.1016/j.bcp.2023.115805

[tjp70572-bib-0037] Schweda, F. , Kurtz, L. , de Wit, C. , Janssen‐Bienhold, U. , Kurtz, A. , & Wagner, C. (2009). Substitution of connexin40 with connexin45 prevents hyperreninemia and attenuates hypertension. Kidney International, 75(5), 482–489. 19109587 10.1038/ki.2008.637

[tjp70572-bib-0038] Sequeira López, M. L. S. , Pentz, E. S. , Nomasa, T. , Smithies, O. , & Gomez, R. A. (2004). Renin cells are precursors for multiple cell types that switch to the renin phenotype when homeostasis is threatened. Developmental Cell, 6(5), 719–728. 15130496 10.1016/s1534-5807(04)00134-0

[tjp70572-bib-0039] Sequeira López, M. L. S. , Pentz, E. S. , Robert, B. , Abrahamson, D. R. , & Gomez, R. A. (2001). Embryonic origin and lineage of juxtaglomerular cells. American Journal of Physiology, 281(2), F345–F356. 11457727 10.1152/ajprenal.2001.281.2.F345

[tjp70572-bib-0040] Siedlecki, A. M. , Jin, X. , Thomas, W. , Hruska, K. A. , & Muslin, A. J. (2011). RGS4, a GTPase activator, improves renal function in ischemia‐reperfusion injury. Kidney International, 80(3), 263–271. 21412219 10.1038/ki.2011.63 PMC3221244

[tjp70572-bib-0041] Singh, R. P. , Franke, K. , Kalucka, J. , Mamlouk, S. , Muschter, A. , Gembarska, A. , Grinenko, T. , Willam, C. , Naumann, R. , Anastassiadis, K. , Stewart, A. F. , Bornstein, S. , Chavakis, T. , Breier, G. , Waskow, C. , & Wielockx, B. (2013). HIF prolyl hydroxylase 2 (PHD2) is a critical regulator of hematopoietic stem cell maintenance during steady‐state and stress. Blood, 121(26), 5158–5166. 23667053 10.1182/blood-2012-12-471185

[tjp70572-bib-0042] Smith, J. P. , Paxton, R. , Medrano, S. , Sheffield, N. C. , Sequeira‐Lopez, M. L. S. , & Gomez, R. A. (2024). Inhibition of Renin expression is regulated by an epigenetic switch from an active to a poised State. Hypertension, 81(9), 1869‐1882 38989586 10.1161/HYPERTENSIONAHA.124.22886 PMC11337216

[tjp70572-bib-0043] Stefanska, A. , Kenyon, C. , Christian, H. C. , Buckley, C. , Shaw, I. , Mullins, J. J. , & Péault, B. (2016). Human kidney pericytes produce renin. Kidney International, 90(6), 1251–1261. 27678158 10.1016/j.kint.2016.07.035 PMC5126097

[tjp70572-bib-0044] Takeda, K. , Ho, V. C. , Takeda, H. , Duan, L.‐J. , Nagy, A. , & Fong, G.‐H. (2006). Placental but not heart defects are associated with elevated hypoxia‐inducible factor α levels in mice lacking prolyl hydroxylase domain protein 2. Molecular and Cellular Biology, 26(22), 8336–8346. 16966370 10.1128/MCB.00425-06 PMC1636770

[tjp70572-bib-0045] Wang, F. , Flanagan, J. , Su, N. , Wang, L.‐C. , Bui, S. , Nielson, A. , Wu, X. , Vo, H.‐T. , Ma, X.‐J. , & Luo, Y. (2012). RNAscope: A novel in situ RNA analysis platform for formalin‐fixed, paraffin‐embedded tissues. The Journal of Molecular Diagnostics, 14(1), 22–29. 22166544 10.1016/j.jmoldx.2011.08.002 PMC3338343

[tjp70572-bib-0046] Wirth, A. , Benyó, Z. , Lukasova, M. , Leutgeb, B. , Wettschureck, N. , Gorbey, S. , Orsy, P. , Horváth, B. , Maser‐Gluth, C. , Greiner, E. , Lemmer, B. , Schütz, G. , Gutkind, J. S. , & Offermanns, S. (2008). G12‐G13‐LARG‐mediated signaling in vascular smooth muscle is required for salt‐induced hypertension. Nature Medicine, 14(1), 64–68. 10.1038/nm1666 18084302

[tjp70572-bib-0047] Yamaguchi, H. , Gomez, R. A. , & Sequeira‐Lopez, M. L. S. (2023). Renin cells, from vascular development to blood pressure sensing. Hypertension, 80(8), 1580–1589. 37313725 10.1161/HYPERTENSIONAHA.123.20577 PMC10526986

[tjp70572-bib-0048] Yin, A. , Fu, W. , Elengickal, A. , Kim, J. , Liu, Y. , Bigot, A. , Mamchaoui, K. , Call, J. A. , & Yin, H. (2024). Chronic hypoxia impairs skeletal muscle repair via HIF‐2α stabilization. Journal of Cachexia Sarcopenia Muscle, 15(2), 631–645. 38333911 10.1002/jcsm.13436 PMC10995261

[tjp70572-bib-0049] Zhang, R. , Wu, Y. , Zhao, M. , Liu, C. , Zhou, L. , Shen, S. , Liao, S. , Yang, K. , Li, Q. , & Wan, H. (2009). Role of HIF‐1α in the regulation ACE and ACE2 expression in hypoxic human pulmonary artery smooth muscle cells. American Journal of Physiology‐Lung Cellular and Molecular Physiology, 297(4), L631–L640. 19592460 10.1152/ajplung.90415.2008

[tjp70572-bib-0050] Zhang, W. , Li, Y. , & Wang, J.‐G. (2024). Hypertension induced by hypoxia‐inducible factor prolyl hydroxylase inhibitors in treating anemia in patients with chronic kidney disease: A mini‐review. Journal of Clinical Hypertension, 26(12), 1375–1383. 39494784 10.1111/jch.14924 PMC11654843

