# Supplementary material for: The endocrine product of renal (preglomerular) contractile pericytes depends on prolyl‐4‐hydroxylases 2 and 3
Source: J Physiol. 2026 May 4;604(12):5031–60. doi: 10.1113/JP290695 (PMC13267681; doi:10.1113/JP290695)
Supplement: Supplementary file 1 — Peer Review History [file TJP-604-5031-s001.pdf]

The Journal of Physiology

The Endocrine Product of Renal (Preglomerular) Contractile Pericytes Depends on the Prolyl-4-Hydroxylases 2 and 3

Bettina Katharina Mariana Firmke, Lena Marie Süß, Anna-Lena Forst, Armin Kurtz, and Katharina Anna-Elisabeth Broeker
DOI: 10.1113/JP290695

Corresponding author(s): Katharina Broeker (katharina.broeker@ur.de)

The referees have opted to remain anonymous.

Review Timeline:

Submission Date:	06-Dec-2025
Editorial Decision:	16-Jan-2026
Revision Received:	16-Mar-2026
Accepted:	17-Apr-2026

Senior Editor: Kim Barrett

Reviewing Editor: Sophie Møller

Transaction Report:

(Note: With the exception of the correction of typographical or spelling errors that could be a source of ambiguity, letters and reports are not edited. Depending on transfer agreements, referee reports obtained elsewhere may or may not be included in this compilation. Referee reports are anonymous unless the Referee chooses to sign their reports.)

Re: JP-RP-2025-290695 "**The Endocrine Product of Renal (Preglomerular) Contractile Pericytes Depends on the Prolyl-4-Hydroxylases 2 and 3**" by Bettina Katharina Mariana Firmke, Lena Marie Süß, Anna-Lena Forst, Armin Kurtz, and Katharina Anna-Elisabeth Broeker

Dear Dr Broeker,

Thank you for submitting your manuscript to The Journal of Physiology. It has been assessed by a Reviewing Editor and by 2 expert referees and we are pleased to tell you that it is potentially acceptable for publication following satisfactory major revision.

Please advise your co-authors of this decision as soon as possible.

The referee reports are copied at the end of this email.

Please address all the points raised and incorporate all requested revisions or explain in your Response to Referees why a change has not been made. We hope you will find the comments helpful and that you will be able to return your revised manuscript within 2 months. If your article is NOT for a Special Issue, you may have 9 months to revise. If you require an extension, please contact journal staff: jp@physoc.org. Please note that this letter does not constitute a guarantee for acceptance of your revised manuscript.

Your revised manuscript should be submitted online using the link in your Author Tasks: Link Not Available. This link is accessible via your account as Corresponding Author; it is not available to your co-authors. If this presents a problem, please contact journal staff (jp@physoc.org). Image files from the previous version are retained on the system. Please ensure you replace or remove any files that are being revised.

If you do not wish to submit a revised version of your manuscript, you must inform our journal staff (jp@physoc.org) or reply to this email to request withdrawal. Please note that a manuscript must be formally withdrawn from the peer review process at one journal before it may be submitted to another journal.

TRANSPARENT PEER REVIEW POLICY: To improve the transparency of its peer review process, The Journal of Physiology publishes online as supporting information the peer review history of all articles accepted for publication. Readers will have access to decision letters, including Editors' comments and referee reports, for each version of the manuscript, as well as any author responses to peer review comments. Referees can decide whether or not they wish to be named on the peer review history document.

ABSTRACT FIGURES: Authors are expected to use The Journal's premium BioRender account to create/redraw their Abstract Figures. Information on how to access this account is here: https://physoc.onlinelibrary.wiley.com/journal/14697793/biorender-access.

This will enable Authors to create and download high-resolution figures. If authors have used the free BioRender service, they can use the instructions provided in the link above to download a high-resolution version suitable for publication.

The link provided should only be used for the purposes of this submission. Authors will be charged for figures created on this account if they are not related to this manuscript submission.

LANGUAGE EDITING AND SUPPORT FOR PUBLICATION: If you would like help with English language editing, or other article preparation support, Wiley Editing Services offers expert help, including English Language Editing, as well as translation, manuscript formatting, and figure formatting at www.wileyauthors.com/eeo/preparation. You can also find resources for Preparing Your Article for general guidance about writing and preparing your manuscript at www.wileyauthors.com/eeo/prepresources.

RIGOUR AND REPRODUCIBILITY: We recommend authors consult The Journal's Rigour and Reproducibility requirements to ensure that their manuscript is suitable for publication: https://physoc.onlinelibrary.wiley.com/pb-assets/hub-assets/physoc/documents/TJP-Rigour-and-Reproducibility-Requirements.pdf

REVISION CHECKLIST:

Check that your Methods section conforms to journal policy: https://jp.msubmit.net/cgi-bin/main.plex?form_type=display_requirements#methods.

Check that data presented conforms to the statistics policy: https://jp.msubmit.net/cgi-bin/main.plex?form_type=display_requirements#statistics.

Upload a full Response to Referees file. To create your 'Response to Referees': copy all the reports, including any comments from the Senior and Reviewing Editors, into a Microsoft Word, or similar, file and respond to each point, using font or background colour to distinguish comments and responses and upload as the required file type.

Please upload two versions of your manuscript text: one with all relevant changes highlighted and one clean version with no changes tracked. The manuscript file should include all tables and figure legends, but each figure/graph should be uploaded as separate, high-resolution files.

You may also upload:

- 'Potential Cover Art' for consideration as the issue's cover image
- Appropriate Supporting Information (Video, audio or data set: see https://jp.msubmit.net/cgi-bin/main.plex?form_type=display_requirements#supp).

We look forward to receiving your revised submission.

If you have any queries, please reply to this email and we will be pleased to advise.

Yours sincerely,

Kim Barrett
Senior Editor
The Journal of Physiology

REQUIRED ITEMS

1) - Author photo and profile. First or joint first authors are asked to provide a short biography (no more than 100 words for one author or 150 words in total for joint first authors) and a portrait photograph. These should be uploaded and clearly labelled together in a Word document with the revised version of the manuscript. See Information for Authors for further details.

2) - Please upload separate high-quality figure files via the submission form.

3) - Please ensure that any tables are editable and in Word format, and wherever possible, embedded in the article file itself.

4) - Please ensure that the Article File you upload is a Word file.

5) - Papers must comply with the Statistics Policy: https://jp.msubmit.net/cgi-bin/main.plex?form_type=display_requirements#statistics.

In summary:

- If n {less than or equal to} 30, all data points must be plotted in the figure in a way that reveals their range and distribution. A bar graph with data points overlaid, a box and whisker plot or a violin plot (preferably with data points included) are acceptable formats.

- If n > 30, then the entire raw dataset must be made available either as supporting information, or hosted on a not-for-profit repository, e.g. FigShare, with access details provided in the manuscript.

- 'n' clearly defined (e.g. x cells from y slices in z animals) in the Methods. Authors should be mindful of pseudoreplication.

- All relevant 'n' values must be clearly stated in the main text, figures and tables.

- The most appropriate summary statistic (e.g. mean or median and standard deviation) must be used. Standard Error of the Mean (SEM) alone is not permitted.

- Exact p values must be stated. Authors must not use 'greater than' or 'less than'. Exact p values must be stated to three significant figures even when 'no statistical significance' is claimed.

6) - Please include an Abstract Figure file and an Abstract Figure legend. An appropriate figure legend, which should not exceed 150 words in length, should be included in the main manuscript file. The Abstract Figure is a piece of artwork designed to give readers an immediate understanding of the research and should summarise the main conclusions. If possible, the image should be easily 'readable' from left to right or top to bottom. It should show the physiological relevance of the manuscript so readers can assess the importance and content of its findings. Abstract Figures should not merely recapitulate other figures in the manuscript. Please try to keep the diagram as simple as possible and without superfluous information that may distract from the main conclusion(s). Abstract Figures must be provided by authors no later than the revised manuscript stage and should be uploaded as a separate file during online submission labelled as File Type 'Abstract Figure'. Please also ensure that you include the figure legend in the main article file. All Abstract Figures should be created using BioRender. Authors should use The Journal's premium BioRender account to export high-resolution images. Details on how to use and access the premium account are included as part of this email.

7) - Please ensure that all figures AND tables have a title and legend, and that they have been cited within the main article text.

EDITOR COMMENTS

Reviewing Editor:

Thank you for your submission to The Journal of Physiology. Your manuscript presents strong and visually compelling data on PHD2/3-dependent endocrine plasticity in renal pericytes and VSMCs. Two expert reviewers have evaluated the study and both highlighted that, while the descriptive data are impressive, the mechanistic support needed to substantiate your central conclusions currently is incomplete. In particular, several key datasets - detailed in the reviewer reports - will be essential to strengthen the mechanistic interpretation.

In some places the paper complies with the statistics policy and in others they do not. In several graphs, $P<0.05$ indicators (*, # etc) are listed and placed and in some graphs exact P-values are also listed, so there is mixed compliance.

Senior Editor:

There are inconsistencies in the way that data and p values are presented across the manuscript.

REFEREE COMMENTS

Referee #1:

Firmke and colleagues investigated the effects of prolyl hydroxylase (PHD) 2 and PHD3 expressed in smooth muscle myosin heavy chain cells on renal juxtaglomerular cells expressing renin. The reviewer requests that the following concerns be addressed prior to publication:

1. Given that a low salt diet and enalapril promote renin production by lowering blood pressure, and that roxadustat has been reported to induce hypertension as a side effect, PHD inhibition may downregulate renin by elevating blood pressure in non-anemic mice, irrespective of the mechanisms proposed in the present study. To address this concern, please provide data on blood pressure in mice.

2. Hypoxia-inducible factors (HIFs) have been reported to upregulate angiotensin-converting enzyme (ACE) in cells (doi: 10.1152/ajplung.90415.2008; 10.1002/jcsm.13436), raising the concern that PHD inhibition may downregulate renin by mitigating the pharmacological effects of ACE inhibition in mice treated with a low salt diet and enalapril, regardless of the proposed mechanisms. To address this, please provide data on plasma ACE concentrations in mice.

3. The current manuscript lacks analyses of erythropoietin protein expression beyond plasma erythropoietin concentrations, which may not accurately reflect renal expression, given that PHD inhibition reportedly promotes erythropoietin production even in anephric patients (doi: 10.1681/ASN.2010010116). Moreover, previous studies have shown that HK-2 cells-a proximal tubular cell line incapable of producing erythropoietin-express the EPO gene in response to exogenous stimuli (doi: 10.3390/cells10020414), suggesting that assessing EPO gene expression may not directly demonstrate erythropoietin production. Therefore, please perform protein expression analyses of erythropoietin in kidneys.

4. The absence of whole-kidney images showing HIF-2α expression makes it difficult to compare the distribution of

erythropoietin and hypoxia. Furthermore, whether a low salt diet and enalapril can induce hypoxic environments even without PHD inhibition remains unclear. Please re-examine HIF-2α expression to address these concerns.

5. Please provide data on hematocrit so that these values can be readily compared to those on plasma erythropoietin concentrations.

Referee #2:

see attachment.

END OF COMMENTS

Prolyl-4-hydroxylases (PHDs) are enzymes that catalyze the O_2-dependent hydroxylation of proline residues in specific target proteins including hypoxia-inducible factors HIF. The present study by Firmke et al investigated the roles of PHD2 and PHD3 in the endocrine production by preglomerular VSMCs in the kidney. The authors found that the co-deletion of PHD2 and PHD3 causes a transformation of VSMCs to erythropoietin (EPO) producing cells. Interestingly, salt depletion, which usually stimulates renin production in normal mice, stimulates EPO production in PHD2/3 deficient mice. The changes involves the hypoxia-inducible factor 2 α. The findings are very interesting. The microscopic images are of high quality, the data were well organized. The story could be strengthened. In its present form, the potential molecular mechanism underlying the observations seems not well interpreted.

Major comments:

1. Please number the lines and pages of the manuscript. It is very difficult to refer to specific contents in the manuscript.
2. Honestly, I am not an expert of development biology. However, I am concerned with the use of the term of “recruitment” of cells in the manuscript. The meaning of “recruitment” is pretty confusing in the context of the paper. Literally, it seems to me that, “recruitment” indicates spatial movement/migration of something that is already present somewhere. However, the increase of the number of specific cells producing renin or EPO is more likely the result of differentiation or functional transformation of the cells. The term “recruit/recruitment” is better replaced with other appropriate expression or needs to be defined more specifically if they are to be retained.
3. The terms “recruitment” and “transformation” are both used in the manuscript. Are they used to refer to the same scenarios or different ones? If they are used in the same sense, maybe it is better to stick to “transformation”.
4. In the second paragraph of Introduction, it is said that “increased EPO production involves the recruitment of additional interstitial PDGFR-β^+ fibroblasts/pericytes

from the corticomedullary border towards the cortical surface". In this sentence, the term "recruitment" clearly indicates a spatial migration of the cells for a very long distance. Is it possible that the pericytes in the corticomedullary border migrate for such a long distance to the cortical surface?

5. While the production of renin and EOP in kidney is nicely introduced in the Introduction, the introduction for the prolyl-4-hydroxylases (PHDs) is very weak. There is almost no description for the molecular roles of PHDs in the introduction. I could not understand the interaction between PHDs and HIF-2a until I searched literature to get some detailed for the PHDs. More molecular details are needed to define the PHDs. I noticed that there are some nice reviews and recent studies on PHD (e.g., Koivunen & Kietzmann, 2018, doi: 10.1016/j.molmed.2018.10.004; Myllykoski et al, 2021, JBC, doi: 10.1074/jbc.RA120.016542).
6. The Graphical Abstract is nice. However, it could be improved. Basically, the graphical abstract just tells the main phenotypes under different conditions and tells little about the potential molecular mechanism underlying the changes. There is no mention of HIF-2α in the diagram.
7. There is no mention of HIF-2α in the Abstract?
8. Several different types of kidney cells were examined in the present study, including vascular smooth muscle cells, renin-producing cells, EPO-producing cells, and pericytes etc. It seems that these cells were defined mainly by the expression of specific molecular markers, on the mRNA level. The microscopic images are all shown in low-magnifications. Can these types of cells be morphologically distinguished? It is worth to show some pictures in higher magnification with more morphological features of different types of cells.
9. The expression of major molecular markers was examined only on the transcript level by using RNAscope. Was the expression of key genes in the kidneys, such as the PHDs, ever examined by using antibodies?
10. If I understand correctly (by searching literature), PHDs causes hydroxylation of HIF-2α, which is O2 dependent. The hydroxylation results in the ubiquitination of HIF-2α. I wonder if there is a change in the ubiquitination level of HIF-2α in the

kidneys, since PHD deletion increases the stability of HIF-2α in mouse kidney under salt depletion as the authors observed.

11. It is very interesting that, in the absence of PHD, salt depletion triggers the activation of HIF-2α signaling, increased EPO production, enhancement of hematocrit, a set of responses similar to those to hypoxia. What is the relationship of PHDs and HIF-2α? Is the activation of HIF-2α signaling and increased EPO production by salt depletion the result of renal hypoxia (decrease in oxygen availability) and/or blood flow? Or is it just a mis-activation due to the deficient of PHDs? What could be the physiological implications of these findings?

Minors:

1. It seems that ACE was not defined throughout the manuscript.
2. There is a logo of the software vendor at the right bottom of the Graphical Abstract.
3. Page 30: Reference is required for the statement "In a previous study, we demonstrated that renin cell-specific deletion of PHD2 and PHD3 induces EPO and upregulates EPO-associated markers, while downregulating renin and renin cell markers" in the first paragraph of Section V.
4. Legend to Figure 1. Although italicized symbols indicate the levels of genes or transcripts according to the common norms, it would be more specific to write out the expression of "mRNA" or "transcript" of specific genes. There are many similar problems throughout the maintext.
5. Figure 7. Please indicate the treatment of normal diet in the left panels.
6. I saw no citation for the reference by Galkin F et al in the text.
7. Keep consistent for the terms. For example, HIF2α is used instead of HIF-2α in the legends to Figs. 5 and 11. Sometimes HIF-2 (without α) is used in the maintext.

Re: JP-RP-2025-290695 "**The Endocrine Product of Renal (Preglomerular) Contractile Pericytes Depends on the Prolyl-4-Hydroxylases 2 and 3**"

by Bettina Katharina Mariana Firmke, Lena Marie Süß, Anna-Lena Forst, Armin Kurtz, and Katharina Anna-Elisabeth Broeker

The authors would like to thank the editors and reviewers for their thorough evaluation of the manuscript and their many helpful comments. We hope that with this revision we have adequately addressed the reviewers' remaining questions and improved the overall quality of the manuscript.

EDITOR COMMENTS

Reviewing Editor:

Thank you for your submission to The Journal of Physiology. Your manuscript presents strong and visually compelling data on PHD2/3-dependent endocrine plasticity in renal pericytes and VSMCs. Two expert reviewers have evaluated the study and both highlighted that, while the descriptive data are impressive, the mechanistic support needed to substantiate your central conclusions currently is incomplete. In particular, several key datasets - detailed in the reviewer reports - will be essential to strengthen the mechanistic interpretation.

In some places the paper complies with the statistics policy and in others they do not. In several graphs, P<0.05 indicators (*, # etc) are listed and placed and in some graphs exact P-values are also listed, so there is mixed compliance.

Senior Editor:

There are inconsistencies in the way that data and p values are presented across the manuscript.

Answer: Dear Editors, thank you for drawing our attention to the inconsistencies in the presentation of the p-values. We have revised the manuscript accordingly to ensure full compliance with the journal's statistical reporting guidelines.

REFEREE COMMENTS

Referee #1:

Firmke and colleagues investigated the effects of prolyl hydroxylase (PHD) 2 and PHD3 expressed in smooth muscle myosin heavy chain cells on renal juxtaglomerular cells expressing renin. The reviewer requests that the following concerns be addressed prior to publication:

1. Given that a low salt diet and enalapril promote renin production by lowering blood pressure, and that roxadustat has been reported to induce hypertension as a side effect, PHD inhibition may downregulate renin by elevating blood pressure in non-anemic mice, irrespective of the mechanisms proposed in the present study. To address this concern, please provide data on blood pressure in mice.

Answer: Thank you for reviewing our manuscript. We measured systolic blood pressure using a tail cuff manometry system in SMMHC^{CreERT2} PHD2^{ff} PHD3^{ff} mice and the corresponding control animals under both normal salt (NS) diet and under low salt/enalapril (LSE) treatment. These data have been integrated into paragraph II of the manuscript as well as into the newly added summary Table 4. Moreover, their implications are discussed in the discussion section.

In brief, there was no difference in the systolic blood pressure between the control group and the SMMHC^{CreERT2} PHD2^{ff} PHD3^{ff} group under NS conditions with mean values of 135.8 ± 6.9 mmHg and 137.4 ± 5.9 mmHg, respectively. Under LSE treatment, systolic blood pressure decreased by approximately 40 mmHg in both groups, reaching 100.5 ± 5.4 mmHg in controls and 97.8 ± 3.7 mmHg in SMMHC-specific PHD2/PHD3 knockout animals, again without a significant difference between genotypes.

Blood pressure measurements under NS conditions were performed 5 weeks after tamoxifen-induced SMMHC-cell-specific deletion of PHD2 and PHD3, at which time knockout mice exhibited markedly elevated hematocrit values (67.2 ± 4.7 %). The sex- and age-matched control group had hematocrit values of 49.5 ± 2.0 %. Systolic blood pressure under LSE conditions was assessed in the same cohort on days 10–14 of LSE treatment.

Collectively, these findings indicate that SMMHC-cell-specific deletion of PHD2 and PHD3 does not alter basal systolic blood pressure under NS conditions and does not impair blood pressure regulation during LSE treatment.

In patients treated with roxadustat, hypertension developed over the course of several weeks of treatment [25]. Therefore, in our experimental study, in which wild-type mice received either NS or LSE for two weeks and were then treated with roxadustat for only 12 hours on the final day, it is unlikely that roxadustat administration affected systolic blood pressure within such a short treatment period. The significant HIF-2α stabilization in the VSMCs did not lead to EPO induction, even though strong renin induction was observed in the VSMCs of LSE-treated animals along the preglomerular vessels suggesting a transformation of these cells to a more endocrine phenotype. This rather indicates that the transformation of VSMCs from renin-like cells to more EPO-like cells requires HIF-2α stabilization for a longer period of time (longer than 12 hours).

Given that systolic blood pressure did not differ between control and SMMHC^{CreERT2} PHD2^{ff} PHD3^{ff} mice under either NS or LSE conditions, and considering that neither the

SMMHC^{CreERT2} PHD2^{ff} nor the SMMHC^{CreERT2} PHD3^{ff} mice exhibited HIF-2α stabilization in VSMCs and both genotypes showed renin induction in response to LSE treatment comparable to LSE-treated control animals, we did not anticipate any deviations in systolic blood pressure. To minimize unnecessary experimental stress for the animals, we therefore chose to omit blood pressure measurements in these groups.

2. Hypoxia-inducible factors (HIFs) have been reported to upregulate angiotensin-converting enzyme (ACE) in cells (doi: 10.1152/ajplung.90415.2008; 10.1002/jcsm.13436), raising the concern that PHD inhibition may downregulate renin by mitigating the pharmacological effects of ACE inhibition in mice treated with a low salt diet and enalapril, regardless of the proposed mechanisms. To address this, please provide data on plasma ACE concentrations in mice.

Answer: Thank you for drawing our attention to the HIF-dependent induction of ACE. Accordingly, we measured plasma ACE concentrations in the different genotypes and treatment groups. The corresponding values have been included in Figures 2 and 7 as well as in Table 4 and the data have been integrated into the results paragraphs I and II of the manuscript. Moreover, their implications are discussed in the discussion section.

Summary of the results: Cell-specific deletion of PHD2, PHD3, or PHD2/PHD3 in SMMHC^{+} cells had no effect on plasma ACE concentrations under NS conditions compared with the controls. In all LSE-treated groups plasma ACE concentrations were elevated by approximately 100 ng/ml compared with ACE levels under a normal-salt diet (from about 300 ng/ml under NS conditions to about 400 ng/ml under LSE conditions). However, there was no significant difference in plasma ACE concentrations between the LSE-treated controls and the respective LSE-treated PHD-KO groups.

These findings provide no evidence that the pharmacological ACE inhibition by LSE treatment is impaired in the PHD2/PHD3-KO animals in a way that would attenuate renin induction. Instead, they support our conclusion that the LSE treatment induces EPO rather than renin production in PHD2/PHD3-deficient VSMCs, that have been transformed into an EPO-cell-like phenotype HIF-2α–dependently.

Additionally, we measured plasma ACE concentrations in the wildtype mice treated either with NS + roxadustat or LSE + roxadustat. Mean ACE levels increased to 683.6 ± 262.2 ng/ml and 1321.7 ± 544.6 ng/ml, respectively (control NS: 344.3 ± 83.0 ng/ml; control LSE: 453.4 ± 134.0 ng/ml). These findings are consistent with a HIF-dependent induction of ACE expression, as reported in the mentioned studies [23, 24]. However, ACE may be induced not only in the kidney (if at all) but also in the lungs and other organs. Whether elevated plasma ACE concentrations would diminish the antihypertensive efficacy of LSE treatment over longer durations remains unclear and would require long-term investigation. Within the short 12-hour observation window examined here, any potential counteracting effect appears minimal, as preglomerular VSMCs could still be observed in their renin-producing endocrine state.

3. The current manuscript lacks analyses of erythropoietin protein expression beyond plasma erythropoietin concentrations, which may not accurately reflect renal expression, given that PHD inhibition reportedly promotes erythropoietin production even in anephric patients (doi: 10.1681/ASN.2010010116). Moreover, previous studies have shown that HK-2 cells-a proximal tubular cell line incapable of producing erythropoietin-express the EPO gene in response to exogenous stimuli (doi: 10.3390/cells10020414), suggesting that assessing EPO gene expression may not directly demonstrate erythropoietin production. Therefore, please perform protein expression analyses of erythropoietin in kidneys.

Answer: We assessed renal EPO protein production by determining the EPO concentration in kidney homogenates in relation to total protein concentrations. The corresponding values have been included in Figures 2 and 7 as well as in Table 4 and the data have been integrated into the results (paragraphs I and II) and discussion section of the manuscript.

Briefly, renal EPO protein concentrations increased significantly from 9.4 ± 1.4 pg/mg in control mice to 14.2 ± 1.8 pg/mg in PHD2-KO mice and to 21.5 ± 2.7 pg/mg in PHD2/PHD3-KO mice under normal salt conditions. PHD3-KO mice had EPO protein concentrations of 9.1 ± 1.3 pg/mg, comparable to controls. Correspondingly, plasma EPO levels rose to 375.3 ± 42.1 pg/ml in PHD2-KO mice and to 656.6 ± 139.3 pg/ml in PHD2/PHD3-KO mice, compared with 195.1 ± 42.8 pg/ml in controls. PHD3-KO mice showed no changes in plasma EPO (218.4 ± 62.4 pg/ml) compared to the control group (see Table 4 in the manuscript).

LSE treatment significantly reduced renal EPO protein concentrations in control and PHD3-KO mice to 6.5 ± 1.0 pg/mg, 7.1 ± 1.5 pg/mg, respectively compared to NS conditions. In LSE-treated PHD2-KO mice renal EPO protein (13.4 ± 2.3 pg/mg) remained unchanged compared to NS conditions, while renal EPO protein concentrations significantly increased in PHD2/PHD3-KO mice (30.7 ± 3.9 pg/mg). This resulted in plasma EPO concentrations of 155.6 ± 26.7 pg/ml in controls, 189.0 ± 45.0 pg/ml in PHD3-KO mice, 357.0 ± 68.1 pg/ml in PHD2-KO mice and 979.0 ± 98.4 pg/ml in PHD2/PHD3-KO mice. Please see also the following table:

	Epo mRNA/ Rpl32 mRNA	Kidney EPO [pg/mg]	Hct [%]	Plasma-EPO [pg/ml]
**control**	1.0 ± 0.6	9.4 ± 1.4	51.3 ± 2.4	195.1 ± 42.8
**control + LSE**	0.1 ± 0.2	6.5 ± 1.0	43.9 ± 3.7	155.6 ± 26.7
**SMMHC^{Cre} PHD2 ^{ff}**	3.2 ± 0.9	14.2 ± 1.8	57.0 ± 4.9	375.3 ± 42.1
**SMMHC^{Cre} PHD2 ^{ff} + LSE**	2.4 ± 0.6	13.4 ± 2.3	56.6 ± 2.1	357.0 ± 68.1
**SMMHC^{Cre} PHD3 ^{ff}**	1.3 ± 0.8	9.1 ± 1.3	49.1 ± 1.8	218.4 ± 62.4
**SMMHC^{Cre} PHD3 ^{ff} + LSE**	0.2 ± 0.3	7.1 ± 1.5	42.8 ± 4.1	189.0 ± 45.0
**SMMHC^{Cre} PHD2 ^{ff} PHD3 ^{ff}**	5.0 ± 1.1	21.5 ± 2.7	68.8 ± 6.4	656.6 ± 139.3
**SMMHC^{Cre} PHD2 ^{ff} PHD3 ^{ff} + LSE**	8.1 ± 1.8	30.7 ± 3.9	75.1 ± 4.8	979.0 ± 98.4
**Wildtype + PHDi**	/	866.8 ± 317.8	48.2 ± 2.1	113,000.0 ± 30,012.2
**Wildtype + LSE + PHDi**	/	777.9 ± 248.2	44.2 ± 1.9	109,807.5 ± 31,408.6

The increase in renal EPO protein concentrations (pg/mg) observed in SMMHC^{CreERT2} PHD2^{ff} and SMMHC^{CreERT2} PHD2^{ff} PHD3^{ff} mice under NS conditions as well as the further elevation of renal EPO protein in SMMHC^{CreERT2} PHD2^{ff} PHD3^{ff} mice following LSE treatment, corresponds well with the respective increases in renal *Epo* mRNA expression and plasma EPO concentrations. The reduced renal EPO protein concentrations determined in LSE treated control and PHD3-KO mice also align with the reduced renal Epo mRNA expression levels seen in these groups compared with their respective NS groups.

For comparison, we also included livers and lungs of control animals and SMMHC^{CreERT2} PHD2^{ff} PHD3^{ff} mice under NS and LSE conditions. However, the EPO protein content remained below the detection limit in both organs under both conditions.

Treatment of wildtype mice with roxadustat (HIF-2α stabilization for 12 hours) resulted in renal EPO protein concentrations of approximately 870 pg/mg (NS + roxadustat) and 780 pg/mg (LSE + roxadustat). Additionally, we detected an EPO protein content of 61.3 ± 7.9 pg/mg (n = 5) and 66.1 ± 4.0 pg/mg (n=5) in the livers of the NS + roxadustat or LSE + roxadustat treated wildtype mice, whereas EPO protein levels in the lungs of these animals remained below the detection limit.

4. The absence of whole-kidney images showing HIF-2α expression makes it difficult to compare the distribution of erythropoietin and hypoxia. Furthermore, whether a low salt diet and enalapril can induce hypoxic environments even without PHD inhibition remains unclear. Please re-examine HIF-2α expression to address these concerns.

Answer: We revised the previous figure depicting HIF-2α staining to more clearly illustrate the zonal distribution of HIF-2α stabilization. The former Figure 5, which had shown only cortical sections, has been updated and now appears as the new Figure 6 in the manuscript. In addition, we generated a new figure (Figure 10) that presents HIF-2α stabilization across the different renal zones in kidneys from both control animals and PHD2/PHD3-KO mice following 14 days of LSE treatment. Because the contrast of the staining is relatively low and HIF-2α–positive signals would be lost in whole-kidney overviews, we opted for a zonal presentation to more clearly illustrate the localization of the HIF-2α–positive cells.

LSE treatment did not induce hypoxia in the kidneys of control animals. Only occasional single HIF-2α positive cells were detectable in the interstitium of the corticomedullary region, similar to what was observed in kidneys from NS-fed controls. VSMCs and interstitial cells in the medullary region were negative for HIF-2α in these LSE-treated control kidneys. Likewise, the distribution and intensity of HIF-2α–positive signals in LSE-treated PHD2/PHD3-KO animals were indistinguishable from those in PHD2/PHD3-KO mice maintained on an NS diet. Here preglomerular VSMCs and interstitial cells in the medullary region were clearly positive for HIF-2α.

5. Please provide data on hematocrit so that these values can be readily compared to those on plasma erythropoietin concentrations.

Answer: We have summarized the hematocrit values together with plasma EPO concentrations, renal EPO mRNA expression, plasma renin concentrations and additional parameters into a single table to allow direct comparison of all metrics at a glance. This overview is presented in Table 4.

Referee #2:

Prolyl-4-hydroxylases (PHDs) are enzymes that catalyze the O2-dependent hydroxylation of proline residues in specific target proteins including hypoxia-inducible factors HIF. The present study by Firmke et al investigated the roles of PHD2 and PHD3in the endocrine production by preglomerular VSMCs in the kidney. The authors found that the co-deletion of PHD2 and PHD3 causes a transformation of VSMCs to erythropoietin (EPO) producing cells. Interestingly, salt depletion, which usually stimulates renin production in normal mice, stimulates EPO production in PHD2/3 deficient mice. The changes involves the hypoxia-inducible factor 2 α. The findings are very interesting. The microscopic images are of high quality, the data were well involveed. The story could be strengthened. In its present form, the potential molecular mechanism underlying the observations seems not well interpreted.

Major comments:

1. Please number the lines and pages of the manuscript. It is very difficult to refer to specific contents in the manuscript.

Answer: Thank you for reviewing our manuscript. We have now added line and page numbers to the manuscript.

2. Honestly, I am not an expert of development biology. However, I am concerned with the use of the term of "recruitment" of cells in the manuscript. The meaning of "recruitment" is pretty confusing in the context of the paper. Literally, it seems to me that, "recruitment" indicates spatial movement/migration of something that is already present somewhere. However, the increase of the number of specific cells producing renin or EPO is more likely the result of differentiation or functional transformation of the cells. The term "recruit/recruitment" is better replaced with other appropriate expression or needs to be defined more specifically if they are to be retained.

Answer: We apologize that our use of the term "recruitment" may have made the manuscript more difficult to understand or introduced ambiguity. Our use of this term was based on its established application in the renin and EPO literature, where it is commonly employed to describe a resident cell that – depending on physiological conditions – begins to produce EPO or renin by converting from a non–hormone-producing into a hormone-producing state (please see [2, 4, 10, 19]). Unfortunately, when writing the manuscript, we did not consider that the term carries additional meanings and may therefore lead to misinterpretation without a clear definition.

To improve the clarity of the manuscript and to describe the underlying physiological processes more precisely, we have replaced the term "recruitment" throughout the manuscript with more explicit terms such as "transformation."

3. The terms "recruitment" and "transformation" are both used in the manuscript. Are they used to refer to the same scenarios or different ones? If they are used in the same sense, maybe it is better to stick to "transformation".

Answer: Yes, both terms were used synonymously to describe the transformation of cells into renin- or EPO-producing cells, indicating that renin or EPO transcription is being induced/activated in those cells. As stated above, we have removed the term 'recruitment' and now consistently use 'transformation' or other context-appropriate terminology throughout the manuscript to enhance clarity.

4. In the second paragraph of Introduction, it is said that “increased EPO production involves the recruitment of additional interstitial PDGFR-β^{+} fibroblasts/pericytes from the corticomedullary border towards the cortical surface”. In this sentence, the term “recruitment” clearly indicates a spatial migration of the cells for a very long distance. Is it possible that the pericytes in the corticomedullary border migrate for such a long distance to the cortical surface?

Answer: We appreciate your comment. With this statement, we did not intend to suggest that the cells migrate from the corticomedullary junction toward the cortical surface. As stated above, our use of the term “recruitment” was based on its usage in the renin and EPO literature, where it is commonly employed to describe a resident cell that—depending on physiological conditions such as decreased oxygen availability or hypotension—begins to produce EPO or renin by converting from a non–hormone-producing into a hormone-producing state (please see [2, 4, 10, 19]). In this context, the term does not imply spatial migration but rather functional activation/transformation to fulfill a physiological task.

To clarify the mechanism accurately: Renal EPO production is carried out by resident PDGFRβ^{+} fibroblasts. Under normoxic conditions, the few resident PDGFRβ^{+} fibroblasts that are producing EPO are located predominantly at the corticomedullary border [9, 18]. When tissue oxygen levels decline, EPO expression is induced in additional interstitial PDGFRβ^{+} fibroblasts. This induction follows a characteristic spatial pattern: initially, more fibroblasts in the deep cortex and the outer stripe of the outer medulla begin producing EPO. If tissue oxygenation declines further, even more interstitial fibroblasts are activated to produce EPO - first in the mid-cortex and finally in the superficial cortex [4]. It was this gradual, spatially expanding pattern of activation that we intended to describe.

We have revised the respective paragraph in the *Introduction* section to clarify that increased EPO production results from the activation/induction of *Epo* transcription in resident PDGFRβ^{+} interstitial fibroblasts located in the cortex and the outer stripe of the outer medulla.

5. While the production of renin and EPO in kidney is nicely introduced in the Introduction, the introduction for the prolyl-4-hydroxylases (PHDs) is very weak. There is almost no description for the molecular roles of PHDs in the introduction. I could not understand the interaction between PHDs and HIF-2a until I searched literature to get some details for the PHDs. More molecular details are needed to define the PHDs. I noticed that there are some nice reviews and recent studies on PHD (e.g., Koivunen&Kietzmann, 2018, doi: 10.1016/j.molmed.2018.10.004; Myllykoski et al, 2021, JBC, doi: 10.1074/jbc.RA120.016542).

Answer: We thank the reviewer for this important and constructive comment. We understand that the introduction did not sufficiently describe the molecular functions of the prolyl-4-hydroxylases (PHDs) and their mechanistic interaction with HIF-2α. In response, we have substantially revised the relevant paragraph in the Introduction to provide a clear explanation of the roles of PHD2 and PHD3 for HIF-2α regulation in the context of renal erythropoietin production.

6. The Graphical Abstract is nice. However, it could be improved. Basically, the graphical abstract just tells the main phenotypes under different conditions and tells little about the potential molecular mechanism underlying the changes. There is no mention of HIF-2α in the diagram.

Answer: We thank the reviewer for these suggestions to improve our graphical abstract. We have substantially revised the graphical abstract and hope that we were able to present the potential molecular mechanism in a clear and accessible manner. In addition, we explicitly incorporated the medullary interstitial *Smmhc*$^{+}$ pericytes.

7. There is no mention of HIF-2α in the Abstract?

Answer: We appreciate you bringing this to our attention. We have revised the abstract to clarify that PHD2/PHD3 codeletion leads to HIF-2α stabilization in VSMCs, and that the respective PHD2 or PHD2/PHD3 deletions result in HIF-2α stabilization in interstitial contractile pericytes.

8. Several different types of kidney cells were examined in the present study, including vascular smooth muscle cells, renin-producing cells, EPO-producing cells, and pericytes etc. It seems that these cells were defined mainly by the expression of specific molecular markers, on the mRNA level. The microscopic images are all shown in low-magnifications. Can these types of cells be morphologically distinguished? It is worth to show some pictures in higher magnification with more morphological features of different types of cells.

Answer: Yes, the cells can in principle be distinguished based on their morphology.

To better illustrate the varying morphology of the analyzed cells, we have revised several figures. In some cases, we adjusted the selected image sections to show them at slightly higher magnification or added insets that display individual cells or regions in greater detail (please see Figures 1, 3, 8, 13 and 14). However, because RNAscope signals for target genes that are only weakly to moderately expressed per cell appear as single dots or small clusters of dots within the cytoplasm, it remains difficult to clearly visualize cell morphology in such cases—even at higher magnification.

This issue is particularly relevant for the interstitial contractile pericytes: both marker genes, Pdgfrb mRNA and Smmhc mRNA, are expressed at moderate or low levels per cell. As a result, the characteristic stellate morphology of these cells—relatively small, partly flattened cells with numerous cellular processes—is not easily discernible using RNAscope alone.

For this reason, we combined SMMHC immunofluorescence (IF) with *Epo* RNAscope to better visualize the morphology of medullary contractile pericytes in control kidneys as well as kidneys from PHD2-, PHD3-, and PHD2/PHD3-knockout mice and simultaneously demonstrate the induction of *Epo* transcription in a subset of these pericytes following PHD2 or PHD2/PHD3 deletion. These results are presented in the newly added Figure 4.

However, this approach is not suitable for all analyzed markers or target genes, because protein detection by immunofluorescence (IF) and mRNA detection by RNAscope require fundamentally different conditions regarding tissue fixation as well as blocking and pretreatment steps. For optimal mRNA detection, we perfuse the kidneys with 10% neutral buffered formalin (NBF) (see Methods section) and then post-fix the tissue for an additional 30–36 hours with gentle agitation. The tissue is subsequently dehydrated through an ascending ethanol–isopropanol series and embedded in paraffin. During the RNAscope

protocol, a key step is tissue permeabilization, which includes a protease treatment. However, the combination of strong fixation and protease digestion makes it difficult (e.g., renin) or even impossible to detect certain proteins with antibodies.

For IF or chromogenic HIF-2α staining, the kidneys are perfused with 3% paraformaldehyde (PFA) and immediately dehydrated through an ascending methanol–isopropanol series before paraffin embedding. This shorter fixation is sufficient for reliable protein detection, but it is too mild to yield robust RNAscope signals.

The co-detection assay developed by ACD/Bio-Techne enables binding of a primary antibody by using a slightly stronger target-retrieval step prior to antibody incubation. After, the tissue sections are then post-fixed with 10% NBF before proceeding with the RNAscope assay, including the protease treatment. However, in this co-detection workflow, both the IF and RNAscope signals are generally somewhat weaker compared with conventional IF or RNAscope performed alone. Thus, target mRNAs with lower expression levels are no longer reliably detectable using this workflow (for example *Phd2* or *Phd3* mRNA expression). Moreover, as mentioned above, not all antibodies that work reliably in standard IF remain compatible with this co-detection protocol.

Since the focus of this study was on *renin* and *Epo* expression and production, as well as changes in cell marker expression, we consistently used the multiplex RNAscope assay to optimally detect the localization as well as changes in expression patterns and expression levels on a per-cell basis.

To illustrate the differing morphology of the analyzed cell types in a direct comparison, we have added an additional Figure 1 here, which shows the morphology of these cells more clearly using immunofluorescence stainings. The figure also includes a detail in higher magnification of a cortical native EPO-producing cell, demonstrating *Epo* mRNA expression codetected with PDGFR-β immunofluorescence.

Briefly, juxtaglomerular renin-producing cells are relatively large, cuboid-like cells located in the media layer of the afferent arteriole at its entry into the glomerulus. Preglomerular vascular smooth muscle cells, in contrast, are more elongated and slender, forming the wall of the afferent arterioles and interlobular arteries [15].

EPO-producing interstitial fibroblasts and interstitial contractile pericytes both belong to the population of PDGFR-β–positive interstitial cells [1]. Both cell types exhibit a stellate morphology because they extend numerous processes that interconnect with processes of other interstitial fibroblasts/pericytes or make close contact with capillary endothelial cells—and, in the case of interstitial fibroblasts, also with tubular epithelial cells [12].

Based on morphology alone (at least not with IF stainings), interstitial fibroblasts (including native EPO-producing cells) cannot be distinguished from interstitial contractile SMMHC-positive pericytes. Both populations are PDGFR-β–positive, but only the contractile pericytes are SMMHC-positive.

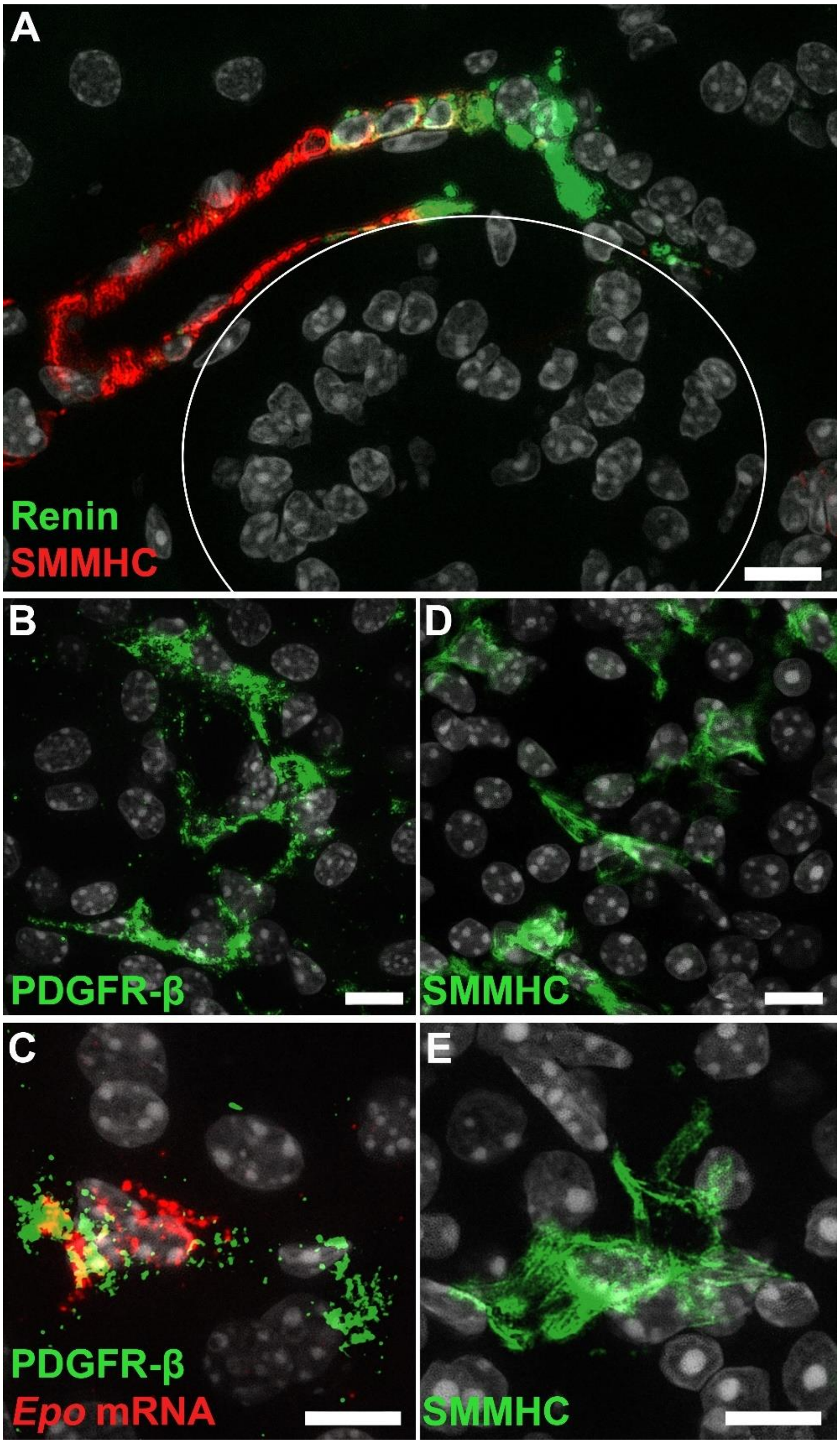

Figure 1: Morphology of renal renin-producing cells, VSMCs, cortical interstitial (EPO-producing) fibroblasts, and contractile pericytes in a wild-type mouse kidney.

(A) Renin-producing cells (green) display a voluminous, cuboid-like morphology and are located in the afferent arteriole at the entrance into the glomerulus (circle). The vascular smooth muscle cells labeled by SMMHC immunofluorescence (red) are slender and elongated.

(B) Cortical interstitial fibroblasts exhibit a characteristic stellate shape, visualized by PDGFR-β immunofluorescence.

(C) A native EPO-producing cell identified by co-detection of PDGFR-β immunofluorescence and *Epo* mRNA using RNAscope.

(D, E) Interstitial contractile pericytes in the outer medulla, visualized by SMMHC immunofluorescence.

All images were acquired using a 63x objective. Nuclei counterstained with DAPI. Scale bars: 10 µm.

9. The expression of major molecular markers was examined only on the transcript level by using RNAscope. Was the expression of key genes in the kidneys, such as the PHDs, ever examined by using antibodies?

Answer: We have previously analyzed typical cellular markers such as PDGFR-β, SMMHC, and renin in earlier studies and could confirm that the expression patterns of the detected mRNAs and their corresponding proteins are consistent [1, 8, 9].

In addition to assessing renin expression by RNAscope and measuring plasma renin concentrations in this study, we also examined the localization of renin-producing cells by immunofluorescence in all genotypes and experimental conditions included in this study. Importantly, we did not observe any differences between the renin-cell localization detected by immunofluorescence and that detected by RNAscope.

As an example, Figure 2 illustrates the localization of renin-producing cells by IF in control animals as well as in SMMHC^{CreERT2} PHD2^{ff} PHD3^{ff} mice under NS and LSE conditions (the corresponding figure in the manuscript is Figure 8).

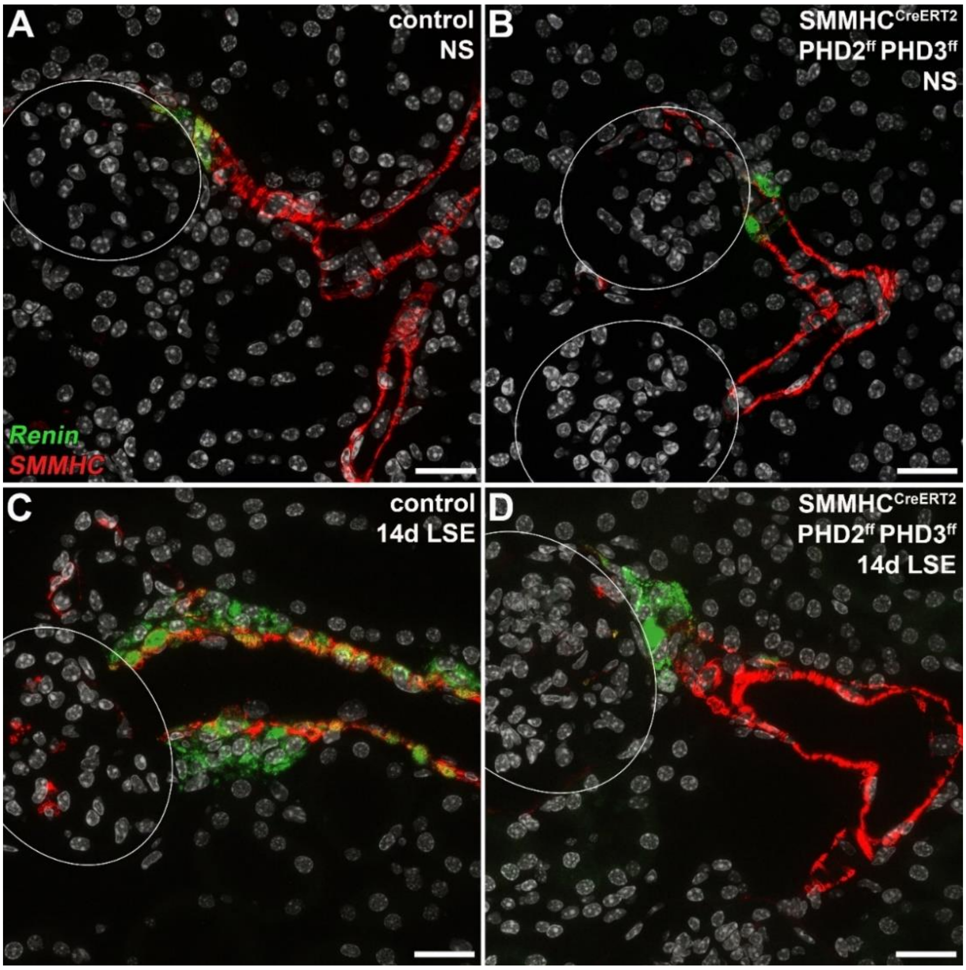

Figure 2: Extent and pattern of Renin production under normal-salt (NS) diet or low-salt/enalapril (LSE) conditions on kidney sections from control and SMMHC^{CreERT2} PHD2^{ff} PHD3^{ff} mice.
A/B: Renin (green) and SMMHC (blue) immunofluorescence on kidney sections from control and PHD2/PHD3-KO mice on a normal salt diet.
C/D: Renin (green) and SMMHC (blue) immunofluorescence on kidney sections from control and PHD2/PHD3-KO mice under LSE conditions. In control mice VSMCs along the afferent arteriole as well as extraglomerular mesangial cells were transformed to additional renin-producing cells. In PHD2/PHD3-KO mice only extraglomerular mesangial cells were transformed to renin-producing cells. Circles highlight glomeruli. Nuclei were counterstained with Dapi (grey). Scale bars: 20µm.

In response to your question, we also examined the distribution of PHD2 and PHD3 in kidneys of control mice by immunofluorescence, using an anti-rabbit HRP-linked secondary antibody (dilution 1:200; CS7074; Cell Signaling Technology, Danvers, MA) in combination with the TSA-based fluorophore Vivid570 (Bio-Techne) to amplify detection signals. To identify the PHD2 or PHD3 positive cells consecutive sections were used. However, we were only able to detect PHD2 protein in distal tubular segments, most prominently in the cortical collecting duct, as demonstrated by its co-localization with aquaporin-2 (AQP2). In contrast, vascular smooth muscle cells, interstitial fibroblasts/pericytes, glomerular cells, and proximal tubular epithelial cells were negative for PHD2 protein (Fig. 3), despite showing clear PHD2 mRNA signals (see Figure 1 in the manuscript and [5]).

Similarly, we were unable to detect PHD3 protein in vascular smooth muscle cells. However, a subset of interstitial fibroblasts/pericytes throughout the kidney displayed clear PHD3 immunofluorescence (Fig. 4). In addition, cortical and medullary collecting duct cells exhibited strong PHD3 protein expression. Occasionally, intraglomerular cells were also positive for PHD3.

These findings are consistent with a previous study that systematically analyzed the mRNA and protein expression patterns of the PHD isoforms in the rat kidney [20]. In agreement with our observations, the authors reported no detectable PHD2 or PHD3 protein in vascular smooth muscle cells. They also identified PHD3 protein—but not PHD2 protein—in interstitial fibroblasts using immunohistochemistry. Furthermore, both PHD2 and PHD3 mRNA and protein were detected in distal tubular segments, whereas in proximal tubules, only mRNA—but no corresponding protein—was detectable [20]. Other groups, however, report for example PHD2 protein immunodetection in proximal tubular cells or PHD3 immunofluorescence in endothelial cells [7, 21]. Thus, the absence of detectable PHD2 or PHD3 protein in VSMCs does not necessarily mean that certain cells do not translate the respective mRNAs. Rather, it suggests that protein abundance lies below the detection threshold of the performed immunofluorescence or immunohistochemistry protocol/antibody. Functional studies support this interpretation. Kobayashi et al. demonstrated that cell-type-specific deletion of PHD2 in FoxD1^{+} stromal cells (which include renal interstitial fibroblasts) was sufficient to induce HIF-2α–dependent EPO expression in a subset of renal interstitial fibroblasts [14], indicating that PHD2 protein must be present and biologically active in these cells even though it is not detectable by IF.

Consistent with these findings, our own previous work using PDGFRβ-specific PHD2, PHD3 or PHD2/PHD3 knockout models demonstrated the presence of two fibroblast subsets with respect to PHD expression—one expressing only PHD2 and another expressing both PHD2 and PHD3. Specifically, PDGFRβ-specific PHD2 deletion alone stabilized HIF-2α and induced EPO expression in a subset of interstitial fibroblasts throughout the kidney, whereas combined PHD2/PHD3 deletion approximately doubled the number of EPO-positive fibroblasts. In contrast, PDGFRβ-specific PHD3 deletion did not induce EPO production [5]. These findings further support the conclusion that interstitial fibroblasts indeed contain functional PHD2 protein, even when it cannot be visualized directly.

In parallel, although we were only able to detect PHD2 and PHD3 at the mRNA level in VSMCs, the findings obtained from the SMMHC-specific PHD deletion models also support the conclusion that these cells possess functional PHD2 and PHD3 proteins: Neither the deletion of PHD2 alone nor the deletion of PHD3 alone was sufficient to stabilize HIF-2α in VSMCs, whereas combined deletion of both PHD2 and PHD3 resulted in robust HIF-2α stabilization.

Consistent with our findings in the PDGFRβ^{+} interstitial cell population, SMMHC-specific deletion of PHD2 alone was sufficient to stabilize HIF-2α in a subset of contractile pericytes

and to induce EPO expression. Combined deletion of PHD2 and PHD3 further increased the number of EPO-expressing pericytes. Together, these results support the conclusion that all contractile pericytes possess functional PHD2, while an additional subset also expresses functional PHD3, in agreement with the staining patterns.

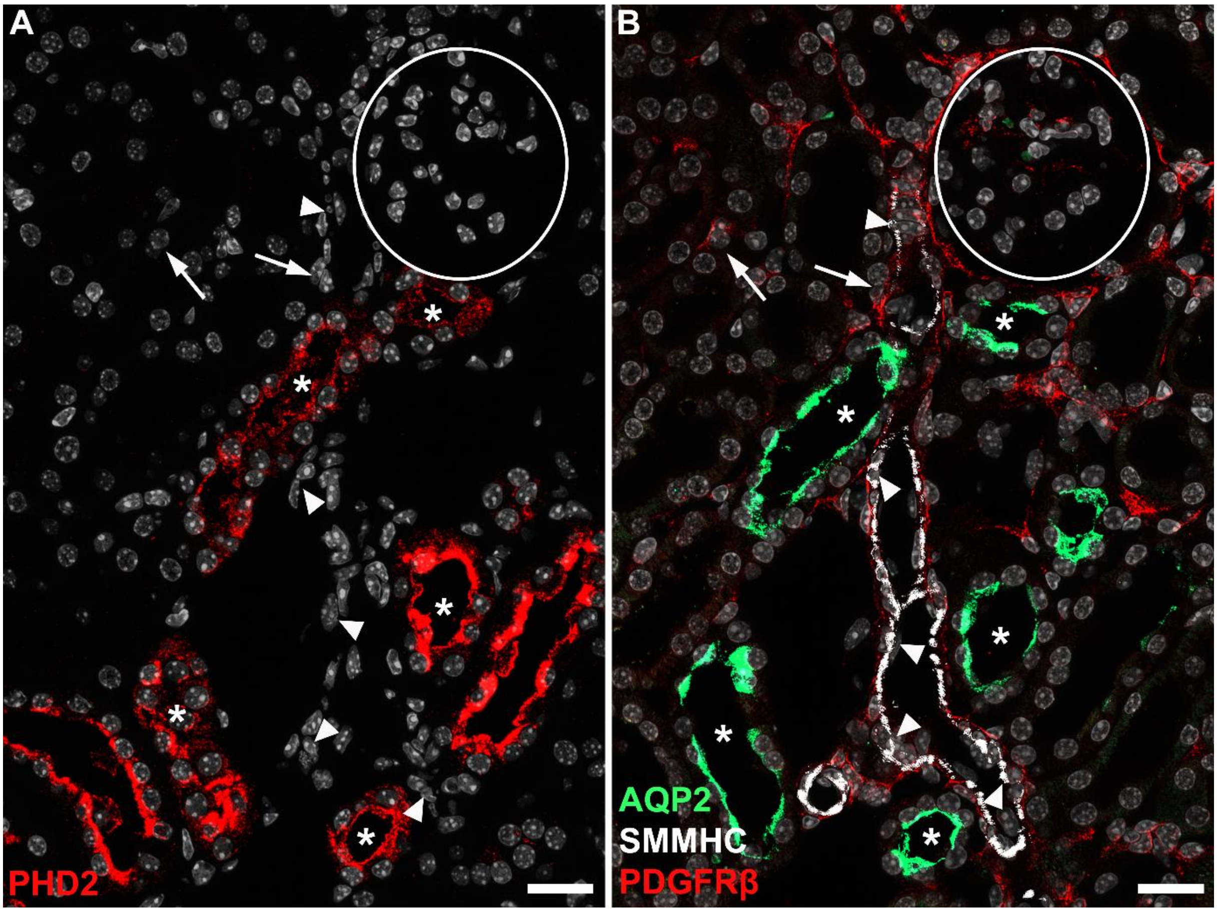

Figure 3: Immunofluorescent detection of PHD2-positive cells in a kidney section from a wild-type mouse**.**

To accurately identify the PHD2-positive cells (red, A) detected using the HRP-TSA–based signal amplification method, corresponding cell-type markers were stained by classical immunofluorescence on the consecutive section (B). The labeled cells were then correlated between the two sections to determine the specific cell type of the PHD2-positive cells. SMMHC (white) served as a marker for VSMCs, PDGFR-β (red) as a marker for interstitial fibroblasts, and AQP2 (green) as a marker for collecting ducts. Asterisks indicate corresponding $PHD2^+/AQP2^+$ collecting ducts. VSMCs (arrowheads) and interstitial fibroblasts (arrows) were negative for PHD2.

Circles highlight the glomerulus. Nuclei were counterstained with Dapi (grey). Scale bars: 20µm.

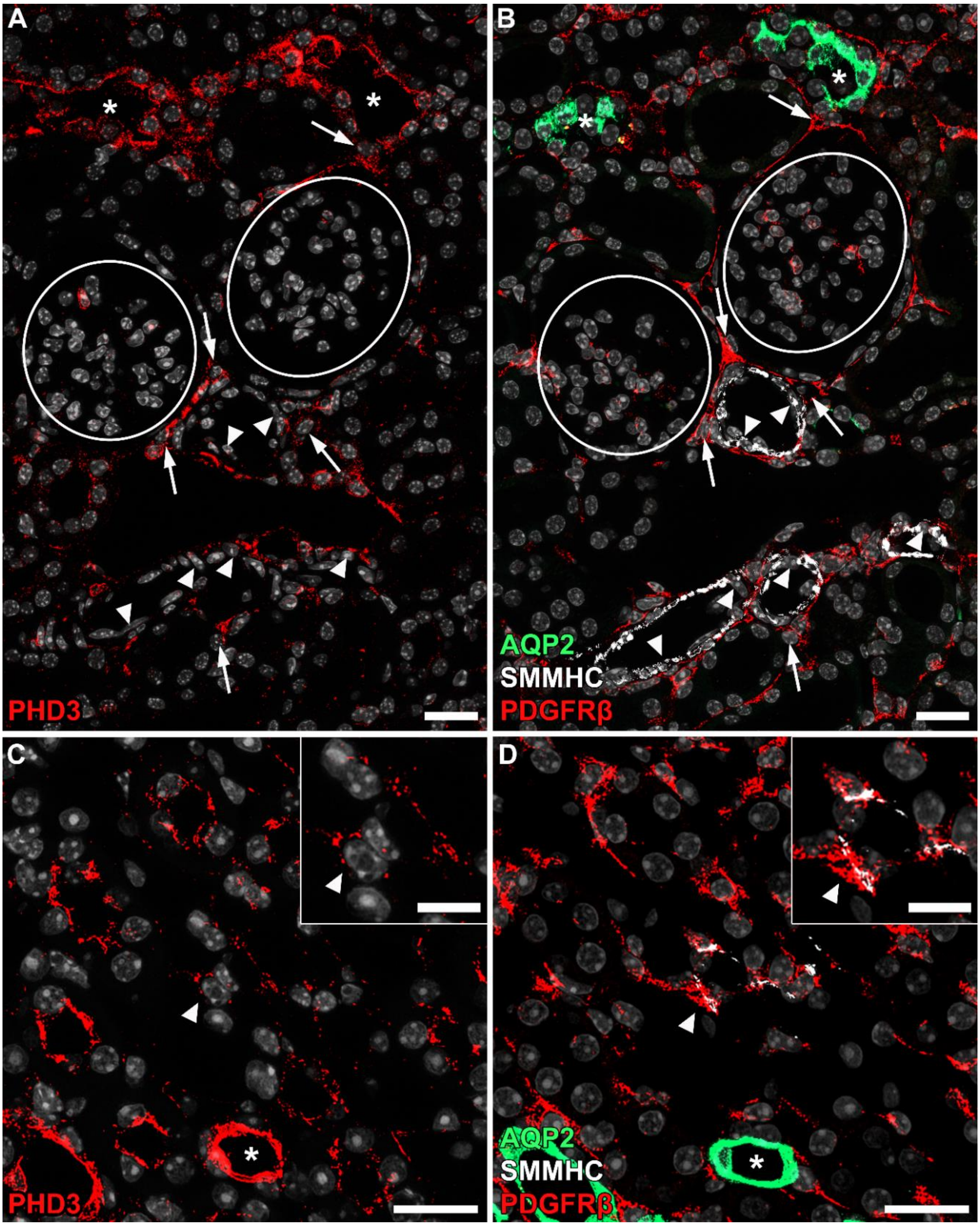

Figure 4: Immunofluorescent detection of PHD3-positive cells in a kidney section from a wild-type mouse**.**

To accurately identify the PHD3-positive cells (red, A/C), corresponding cell-type markers were stained by immunofluorescence on the consecutive section (B/D). The labeled cells were then correlated between the two sections to determine the specific cell types of the PHD3-positive cells. SMMHC (white) was used as a marker for VSMCs, PDGFR-β (red) as a marker for interstitial fibroblasts, and AQP2 (green) as a marker for collecting ducts. Asterisks indicate corresponding $PHD3^+/AQP2^+$ collecting duct cells. Arrows mark $PHD3^+$ interstitial fibroblasts, while arrowheads highlight VSMCs, which were negative for PHD3. Circles denote the glomerulus. Panels C and D show a corresponding detail from the outer medulla. The arrowhead in these panels marks a $PHD3^+/SMMHC^+/PDGFR\text{-}\beta^+$ contractile pericyte; the inset in the upper right corner displays this cell at higher magnification (scale bar: 10 µm). Nuclei were counterstained with DAPI (grey). Scale bars: 20 µm.

Immunofluorescence

Kidney sections (5 µm) were blocked in phosphate-buffered saline containing 10% horse serum and 1% bovine serum albumin before incubation with primary antibodies overnight at 4 °C. After three washes in PBS, sections were incubated with Cy2-, Cy5-, or tetramethylrhodamine-isothiocyanate–conjugated secondary antibodies (Dianova, Hamburg, Germany). Finally, the slides were counterstained with Dapi and mounted using DakoCytomation Glycergel mounting medium (DakoCytomation, Glostrup, Denmark).

For immunodetection of PHD2 and PHD3, the sections were permeabilized in Target Retrieval Solution (Agilent Technologies, Waldbronn, Germany), treated with a 3% hydrogen peroxide solution, and blocked with Serum-Free Protein Block (Agilent Technologies). The sections were then incubated at 4 °C overnight with the respective primary antibody, diluted in 1% BSA/PBS (1:100). The next day, the sections were washed with PBS, incubated with donkey anti-rabbit HRP-linked antibody (dilution 1:200; CS7074; Cell Signaling Technology, Danvers, MA) for 30 minutes at room temperature. For signal detection the TSA-based fluorophore Vivid570 (Bio-Techne) was used. The slides were counterstained with Dapi and mounted using Prolong Gold Antiface mountant (Thermo Fisher Scientific Inc.).

Primary Antibodies:

- SMMHC: rabbit polyclonal to smooth muscle myosin heavy chain 11, dilution 1:100; ab53219, Abcam Inc., Waltham, MA, USA
- Renin: goat polyclonal to renin 1, dilution 1:400; AF4277, Bio-Techne, Minneapolis, MN, USA
- PDGFR-β: rabbit monoclonal to platelet derived growth factor receptor beta, dilution 1:50; ab32570, Abcam Inc.
- PHD2: rabbit polyclonal to EGLN1/PHD2, dilution 1:100; NB100-137; Bio-Techne
- PHD3: rabbit polyclonal to EGLN3/PHD3, dilution 1:100; NB100-139; Bio-Techne
- AQP2: goat polyclonal to aquaporine 2, dilution 1:100; sc-9882, Santa Cruz Biotechnology Inc., Heidelberg, Deutschland

10. If I understand correctly (by searching literature), PHDs causes hydroxylation of HIF-2α, which is O_2 dependent. The hydroxylation results in the ubiquitination of HIF-2α. I wonder if there is a change in the ubiquitination level of HIF-2α in the kidneys, since PHD deletion increases the stability of HIF-2α in mouse kidney under salt depletion as the authors observed.

Answer: You are correct. In principle, the PHD enzymes (PHD2 and PHD3 in our study) use oxygen to hydroxylate the HIF-α subunits at two distinct proline residues. This hydroxylation enables the von Hippel–Lindau protein complex (pVHL), which possesses E3 ubiquitin ligase activity, to recognize and ubiquitinate the HIF-2α subunit. Ubiquitination then marks the corresponding HIF-2α protein for proteasomal degradation [16].

If oxygen levels decline or if the PHD isoforms are deleted, this hydroxylation reaction is reduced or does not occur at all. Without hydroxylation, the HIF-2α subunit is no longer ubiquitinated by pVHL and therefore escapes proteasomal degradation. As a result, the stabilized HIF-2α protein can translocate into the nucleus, where it forms a complex with HIF-1β and additional co-factors (CBP and p300). This complex binds to hypoxia-response elements (HREs) and activates transcription of the corresponding target genes (in our case, for example, Epo) (see schematic Fig. 5 below) [11, 16].

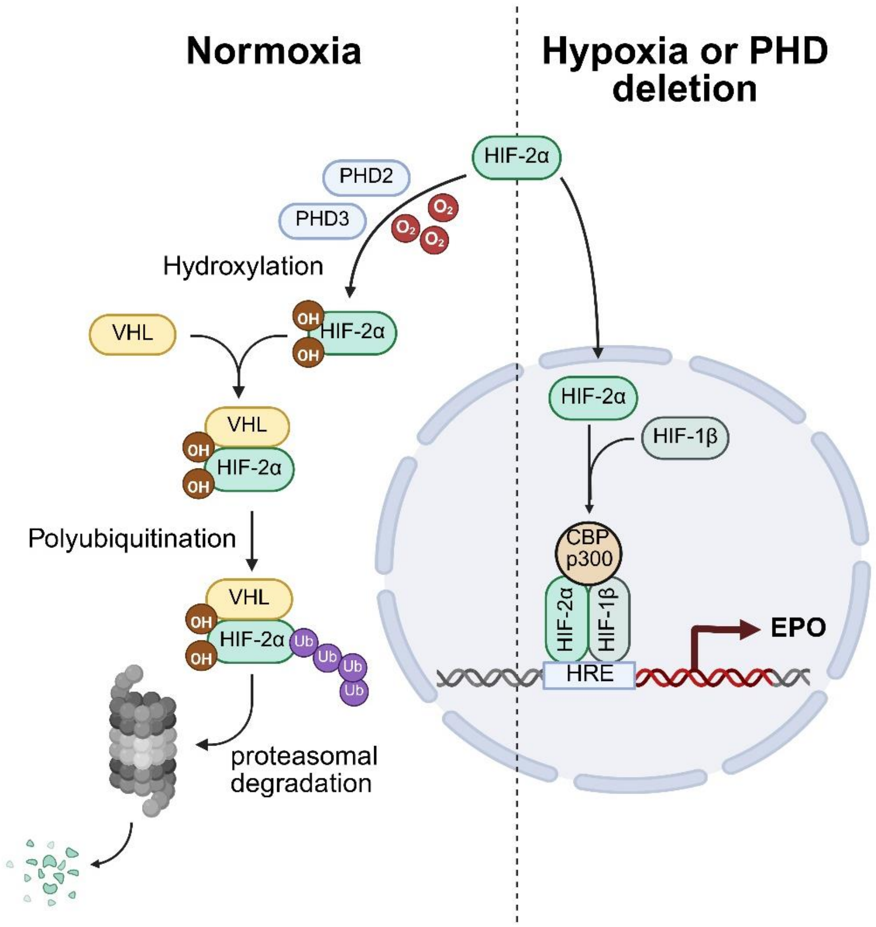

Figure 5: Schematic Representation of the HIF Signaling Pathway Illustrated by the Regulation of Erythropoietin Transcription via HIF-2α. Created with BioRender.com®

A commonly used method to detect ubiquitinated substrates in tissues, according to the literature, is to enrich ubiquitinated proteins from lysates using anti-ubiquitin antibodies and then identify both the ubiquitinated proteins and their ubiquitination sites with highly sensitive methods like mass spectrometry [22]. To detect a specific ubiquitinated substrate (described usually in cultivated cells), the classical method described in the literature is the Western blot. In this approach, proteins are separated by conventional SDS-PAGE, transferred onto a membrane, and detected using specific anti-ubiquitin and specific target-protein antibodies. Ubiquitination alters the molecular weight and thus the migration pattern of a protein, allowing ubiquitinated forms to be visualized. However, the literature also states that immunoblots often lack accuracy when it comes to quantitatively analyzing ubiquitination levels [3].

Because SMMHC^{+} VSMCs and contractile pericytes represent only a small proportion of all kidney cells, only a minor subset of renal cells in our respective SMMHC-PHD deletion models actually carries the respective PHD deletion and would therefore show reduced ubiquitination levels. The vast majority of HIF-2α expressing renal cells remain unchanged. Consequently, we consider the immunoblot method insufficiently sensitive to directly assess, cell-type–restricted changes in HIF-2α ubiquitination in our mouse models.

Based on the established mechanism of HIF-α degradation and stabilization, we hypothesize that HIF-2α ubiquitination is reduced in SMMHC$^+$ VSMCs in which both PHD2 and PHD3 have been deleted, meaning HIF-2α is stabilized in these VSMCs. In SMMHC$^+$ VSMCs where only PHD2 or only PHD3 is deleted, the remaining PHD isoform can still hydroxylate the HIF-2α subunit, allowing it to be ubiquitinated and subsequently degraded.

For the SMMHC$^+$ contractile pericytes, it is necessary to distinguish whether they express only PHD2 or both PHD2 and PHD3.

- In the subpopulation that expresses only PHD2, deletion of PHD2 leads to reduced ubiquitination of HIF-2α and thus to HIF-2α stabilization.
- In the subpopulation that expresses both PHD2 and PHD3, ubiquitination of HIF-2α decreases (markedly) only when **both** isoforms are deleted.

Our immunohistochemical analyses of HIF-2α confirm these hypotheses. Only in cells in which HIF-2α protein is not hydroxylated, ubiquitinated, and degraded—either due to a lack of oxygen (hypoxia) or because PHDs have been deleted or inhibited and therefore cannot exert their enzymatic function—does the non-hydroxylated (= stabilized) HIF-2α translocate to the nucleus. HIF-2α can then be detected as a brown nuclear staining.

Accordingly, in control kidneys we observed only very few interstitial cells with brown nuclear staining, indicating HIF-2α stabilization. These HIF-2α–positive cells were mainly located along the corticomedullary border or the deeper cortex, where the native EPO-producing cells are found.

SMMHC-specific deletion of PHD3 alone had no detectable effect on HIF-2α stabilization in VSMCs or contractile pericytes. The overall staining pattern was comparable to that observed in control kidneys. After SMMHC-specific PHD2 deletion, some HIF-2α–positive nuclei could be detected in the interstitium of the outer medulla. However, VSMCs remained negative for HIF-2α, suggesting that PHD3 is sufficient to hydroxylate HIF-2α, thereby preventing detectable HIF-2α stabilization.

In contrast, after combined PHD2/PHD3 deletion, we observed clear HIF-2α nuclear staining in both VSMCs and interstitial cells of the outer medulla, with the number of HIF-2α–positive interstitial nuclei exceeding those observed in the PHD2-KO mice (please see also Figure 6 in the manuscript).

We also examined kidneys from control and PHD2/PHD3-KO mice treated with LSE. In control animals, LSE treatment did not induce renal hypoxia. Only rarely, HIF-2α positive cells were detected within the interstitium of the corticomedullary region, closely resembling the pattern observed in kidneys from NS-fed control mice (but even less HIF-2α positive cells than in NS controls). VSMCs and interstitial cells in the medullary region were negative for HIF-2α in LSE-treated controls.

Similarly, in LSE-treated PHD2/PHD3-KO mice, the distribution and intensity of HIF-2α positive nuclear signals did not differ from those observed in PHD2/PHD3-KO animals maintained on an NS diet. In both conditions, VSMCs and interstitial cells in the medullary region showed clear nuclear HIF-2α positivity.

To further assess changes in HIF-2α stabilization – and thereby indirectly evaluate alterations in ubiquitination – we performed classical HIF-2α Western blot analyses using whole-kidney lysates from NS- and LSE-treated control and PHD2/PHD3-KO mice. In control animals, HIF-2α levels were barely detectable by immunoblotting, consistent with the fact that under normoxic conditions HIF-2α is not stabilized but instead continuously hydroxylated and degraded. In contrast, PHD2/PHD3 deletion resulted in a significant increase in HIF-2α

stabilization under normal salt conditions (p = 0.0443). LSE treatment led to a slight, though not statistically significant, reduction in renal HIF-2α stabilization in LSE-treated controls compared with NS-fed controls. Conversely, LSE treatment led to a significant further increase in HIF-2α stabilization in PHD2/PHD3-KO mice compared with the PHD2/PHD3-KO group under NS conditions (p = 0.0327).

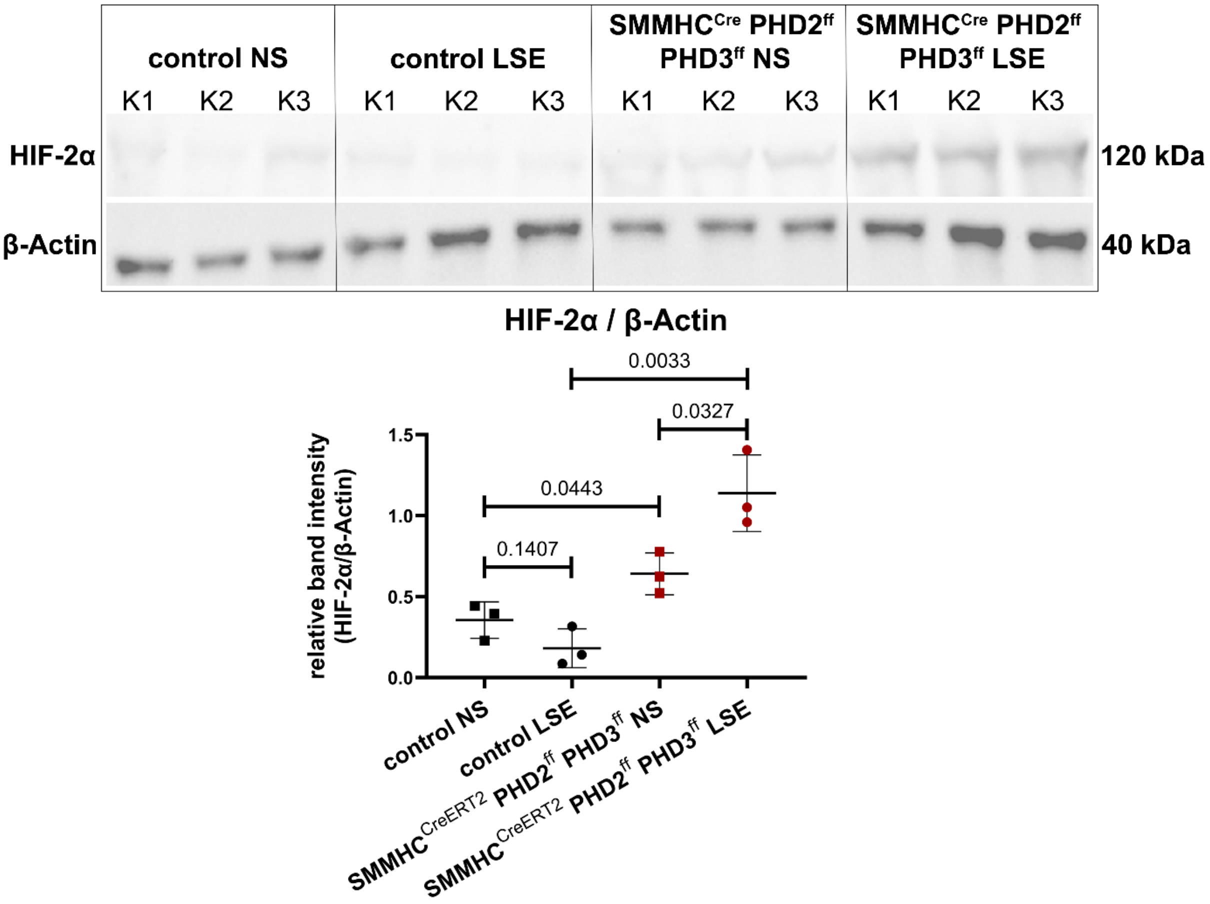

Figure 6: Immunoblot showing HIF-2α stabilization in NS- and LSE-treated control and SMMHC^{CreERT2} PHD2^{ff} PHD3^{ff} mice

(A) Western blots for HIF-2α and β-Actin in kidneys of control and SMMHC^{CreERT2} PHD2^{ff} PHD3^{ff} mice fed either a normal salt (NS) diet or a low salt diet in combination with enalapril (LSE) (n = 3 per genotype and condition).

(B) Densitometric quantification of HIF-2α expression. Data are presented as mean ± SD. Statistical significance was determined using unpaired t-test, two-tailed. The respective p values are stated above the lines.

The stronger HIF-2α band intensities observed in LSE-treated PHD2/PHD3-deficient mice compared with NS-fed PHD2/PHD3-deficient mice support our hypothesis that the LSE-induced drop in systolic blood pressure promotes the transition of contractile/EPO-like VSMCs into a more endocrine-competent state. Under NS conditions, chronic HIF-2α stabilization resulting from PHD2/PHD3 deletion shifts these VSMCs from a contractile/renin-like toward a contractile/EPO-like transcriptional signature without inducing EPO production, although HIF-2α target genes such as Adm and Cd73 are readily expressed. Hypotension then acts as the physiological trigger—analogous to renin induction in native preglomerular VSMCs during low-salt/ACE-inhibitor–induced hypotension—enabling these reprogrammed cells to initiate an endocrine response.

This transition likely allows the HIF-2α/HIF-1β complex to access and bind the *Epo* hypoxia-response element (HRE), thereby initiating *Epo* transcription. As more HIF-2α becomes DNA-bound—to the *Epo* HRE in addition to the binding to the other HRE-regulated loci such as *Adm* and *Cd73*—its stability and detectability may increase, accounting for the enhanced band intensity under LSE conditions. Notably, this interpretation aligns with our immunohistochemistry data, which show no increase in the number of HIF-2α–positive cells following LSE treatment compared with NS conditions.

Immunoblot analysis of HIF-2α and β-Actin

Kidneys were sonified in lysis buffer, and the lysates were centrifuged for 5 minutes at 10,000 x g at 4 °C. Protein concentrations were determined in the supernatants, and equal amounts of total protein were separated by SDS-PAGE on 10% polyacrylamide gels. Proteins were transferred to nitrocellulose membranes, which were blocked for 5 minutes in EveryBlot Blocking Buffer (Bio-Rad Laboratories, Inc., Hercules, CA, USA) and subsequently incubated overnight at 4 °C with primary antibodies against HIF-2α (NB100-122; 1:500; Bio-Techne) or β-actin (ab8227; 1:2500; Abcam).

Following washing, membranes were incubated for 1 hour at room temperature with the appropriate HRP-conjugated secondary antibodies (1:2000; Santa Cruz Biotechnology). Signal detection was performed using a chemiluminescence-based detection system. The band densities were measured using ImageJ.

11. It is very interesting that, in the absence of PHD, salt depletion triggers the activation of HIF-2α signaling, increased EPO production, enhancement of hematocrit, a set of responses similar to those to hypoxia. What is the relationship of PHDs and HIF-2α? Is the activation of HIF-2α signaling and increased EPO production by salt depletion the result of renal hypoxia (decrease in oxygen availability) and/or blood flow? Or is it just a mis-activation due to the deficiency of PHDs? What could be the physiological implications of these findings?

Answer:

Based on our findings, we propose the following mechanism:

- Loss of PHD2 and PHD3 in preglomerular VSMCs results in constitutive HIF-2α stabilization already under normal salt conditions, independent of oxygen availability.
- This chronic stabilization shifts the transcriptional signature from a contractile/renin-like state toward a more contractile/EPO-like identity but does not induce EPO production.

- Only when these PHD-deficient VSMCs are subsequently exposed to a physiological stimulus that normally induces renin (i.e., hypotension from salt depletion and ACE inhibition) do they transform into EPO-producing cells.
- In control mice, salt depletion and ACE inhibition do not stabilize HIF-2α but induce a reversible switch of preglomerular VSMCs into a renin-producing endocrine state.
- In interstitial contractile pericytes, PHD2 or combined PHD2/PHD3 deletion induces robust HIF-2α–dependent EPO production irrespective of systolic blood pressure. However, their localization in the outer medulla along the vasa recta, a permanent low blood flow environment [17], may continuously prime them for endocrine activity.

Therefore, the salt depletion–induced EPO production in PHD-deficient VSMCs reflects a mis-activation of the endocrine response due to the altered HIF-2α-dependent transcriptional state, rather than reflecting renal hypoxia or reduced blood flow. In other words, hypotension provides the trigger for endocrine activation, but the specific endocrine product (renin vs. EPO) is dictated by the HIF-2α-dependent transcriptional signature.

Physiological implications

1. **Endocrine plasticity of VSMCs involves two separable steps.**
   Our data suggest that the reversible transformation of VSMCs into renin-producing cells is not a single process but consists of:
   (i) a reversible transition from a contractile to a more endocrine-permissive state
   (ii) the production of a specific endocrine factor, determined by the cell's current transcriptional signature (renin-like vs. EPO-like)
   Chronic HIF-2α stabilization alters this signature, redirecting the endocrine output from renin to EPO.

2. **PHD2 and PHD3 act as central regulators of endocrine identity.**
   By controlling HIF-2α stabilization, PHD2 and PHD3 determine whether VSMCs adopt a renin- or EPO-producing phenotype, while not impairing the overall plasticity that allows these transitions to remain reversible.

3. **Previously unrecognized EPO-producing cell populations exist.**
   Beyond the established PDGFRβ^{+} interstitial cell populations capable of producing EPO (including contractile pericytes) our findings indicate that additional, previously overlooked cell types such as preglomerular VSMCs also possess the capacity to synthesize EPO. Identifying the mechanisms that govern the switch between renin and EPO production may deepen our understanding of their regulation and uncover therapeutic strategies to selectively modulate these factors.

4. **Potential clinical relevance for CKD and PHD inhibitor therapy.**
   In chronic kidney disease, progressive loss or dedifferentiation of canonical EPO-producing fibroblasts reduces responsiveness to PHD inhibitors [6, 13]. Our results raise the possibility that preglomerular VSMCs and contractile pericytes might serve as additional or alternative EPO-producing cell types under chronic HIF-2α stabilization. Long-term studies will be needed to determine whether prolonged PHD inhibitor treatment can reprogram these cells toward an EPO-competent state.

Minors:

1. It seems that ACE was not defined throughout the manuscript.

Answer: We appreciate you bringing this to our attention. We have now defined ACE as angiotensin-converting enzyme in the introduction.

2. There is a logo of the software vendor at the right bottom of the Graphical Abstract.

Answer: The previous logo has been removed. Following the revision, we gained access to the journal's official BioRender account. The graphical abstract has now been updated to display the official Journal of Physiology logo in accordance with the guidelines.

3. Page 30: Reference is required for the statement "in a previous study, we demonstrated that renin cell-specific deletion of PHD2 and PHD3 induces EPO and upregulates EPO-associated markers, while downregulating renin and renin cell markers" in the first paragraph of Section V.

Answer: We have added the appropriate reference.

4. Legend to Figure 1. Although italicized symbols indicate the levels of genes or transcripts according to the common norms, it would be more specific to write out the expression of "mRNA" or "transcript" of specific genes. There are many similar problems throughout the main text.

Answer: We have revised the manuscript accordingly and added the term 'mRNA' or 'transcript' when referring to the expression of the target genes in both the figure legends and the main text, to improve clarity and comprehensibility throughout.

5. Figure 7. Please indicate the treatment of normal diet in the left panels.

Answer: We updated Figure 7 accordingly.

6. I saw no citation for the reference by Galkin F et al in the text.

Answer: Thank you for bringing this to our attention. We removed the incorrect reference.

7. Keep consistent with the terms. For example, HIF2α is used instead of HIF-2α in the legends to Figs. 5 and 11. Sometimes HIF-2 (without α) is used in the main text.

Answer: Thank you for drawing our attention to this issue. We have revised the manuscript accordingly and standardized the terminology to 'HIF-2α' throughout to ensure clarity and consistency.

References

1. Broeker KAE, Fuchs MAA, Schrankl J, Kurt B, Nolan KA, Wenger RH, Kramann R, Wagner C, Kurtz A (2020) Different subpopulations of kidney interstitial cells produce erythropoietin and factors supporting tissue oxygenation in response to hypoxia in vivo. Kidney Int 98:918–931. doi: 10.1016/j.kint.2020.04.040

2. Dahl SL, Pfundstein S, Hunkeler R, Dong X, Knöpfel T, Spielmann P, Scholz CC, Nolan KA, Wenger RH Fate-mapping of erythropoietin-producing cells in mouse models of hypoxaemia and renal tissue remodelling reveals repeated recruitment and persistent functionality. Acta Physiol n/a:e13768. doi: 10.1111/apha.13768

3. De Silva ARI, Page RC (2023) Ubiquitination detection techniques. Exp Biol Med 248:1333–1346. doi: 10.1177/15353702231191186

4. Eckardt K-U, Koury ST, Tan CC, Schuster SJ, Kaissling B, Ratcliffe PJ, Kurtz A (1993) Distribution of erythropoietin producing cells in rat kidneys during hypoxic hypoxia. Kidney Int 43:815–823. doi: 10.1038/ki.1993.115

5. Firmke BKM, Fuchs MAA, Süß LM, Forst A-L, Kurtz A, Broeker KA-E (2025) Hypoxia-inducible factor-2 stabilization is not sufficient to induce erythropoietin production in deeper medullary fibroblasts. J Physiol 603:5777–5804. doi: 10.1113/JP288798

6. Fuchs MAA, Broeker KAE, Schrankl J, Burzlaff N, Willam C, Wagner C, Kurtz A (2021) Inhibition of transforming growth factor β1 signaling in resident interstitial cells attenuates profibrotic gene expression and preserves erythropoietin production during experimental kidney fibrosis in mice. Kidney Int 100:122–137. doi: 10.1016/j.kint.2021.02.035

7. Futatsugi K, Tokuyama H, Shibata S, Naitoh M, Kanda T, Minakuchi H, Yamaguchi S, Hayashi K, Minamishima YA, Yanagita M, Wakino S, Itoh H (2016) Obesity-induced kidney injury is attenuated by amelioration of aberrant PHD2 activation in proximal tubules. Sci Rep 6:36533. doi: 10.1038/srep36533

8. Gerl K, Miquerol L, Todorov VT, Hugo CPM, Adams RH, Kurtz A, Kurt B (2015) Inducible glomerular erythropoietin production in the adult kidney. Kidney Int 88:1345–1355. doi: 10.1038/ki.2015.274

9. Gerl K, Nolan KA, Karger C, Fuchs M, Wenger RH, Stolt CC, Willam C, Kurtz A, Kurt B (2016) Erythropoietin production by PDGFR-β+ cells. Pflüg Arch - Eur J Physiol 468:1479–1487. doi: 10.1007/s00424-016-1829-2

10. Guessoum O, Zainab M, Sequeira- Lopez MLS, Gomez RA (2020) Proliferation does not contribute to murine models of renin cell recruitment. Acta Physiol Oxf Engl 230:e13532. doi: 10.1111/apha.13532

11. Haase VH (2013) Hypoxic regulation of erythropoiesis and iron metabolism. Am J Physiol - Ren Physiol 304:F1–F13

12. Kaissling B, Le Hir M (2008) The renal cortical interstitium: morphological and functional aspects. Histochem Cell Biol 130:247–262. doi: 10.1007/s00418-008-0452-5

13. Kobayashi H, Davidoff O, Pujari-Palmer S, Drevin M, Haase VH (2022) EPO synthesis induced by HIF-PHD inhibition is dependent on myofibroblast transdifferentiation and colocalizes with non-injured nephron segments in murine kidney fibrosis. Acta Physiol 235:e13826. doi: 10.1111/apha.13826

14. Kobayashi H, Liu Q, Binns TC, Urrutia AA, Davidoff O, Kapitsinou PP, Pfaff AS, Olauson H, Wernerson A, Fogo AB, Fong G-H, Gross KW, Haase VH (2016) Distinct subpopulations of FOXD1 stroma-derived cells regulate renal erythropoietin. J Clin Invest 126:1926–1938. doi: 10.1172/JCI83551

15. Kurt B, Kurtz A (2015) Plasticity of renal endocrine function. Am J Physiol Regul Integr Comp Physiol 308:R455-466. doi: 10.1152/ajpregu.00568.2013

16. Lee P, Chandel NS, Simon MC (2020) Cellular adaptation to hypoxia through hypoxia inducible factors and beyond. Nat Rev Mol Cell Biol 21:268–283. doi: 10.1038/s41580-020-0227-y

17. Mattson DL (2003) Importance of the renal medullary circulation in the control of sodium excretion and blood pressure. Am J Physiol-Regul Integr Comp Physiol 284:R13–R27. doi: 10.1152/ajpregu.00321.2002

18. Maxwell PH, Osmond MK, Pugh CW, Heryet A, Nicholls LG, Tan CC, Doe BG, Ferguson DJ, Johnson MH, Ratcliffe PJ (1993) Identification of the renal erythropoietin-producing cells using transgenic mice. Kidney Int 44:1149–1162. doi: 10.1038/ki.1993.362

19. Neubauer B, Machura K, Kettl R, Lopez MLSS, Friebe A, Kurtz A (2013) Endothelium-Derived Nitric Oxide Supports Renin Cell Recruitment Through the Nitric Oxide–Sensitive Guanylate Cyclase Pathway. Hypertension 61:400–407. doi: 10.1161/HYPERTENSIONAHA.111.00221

20. Schödel J, Klanke B, Weidemann A, Buchholz B, Bernhardt W, Bertog M, Amann K, Korbmacher C, Wiesener M, Warnecke C, Kurtz A, Eckardt K-U, Willam C (2009) HIF-prolyl hydroxylases in the rat kidney: physiologic expression patterns and regulation in acute kidney injury. Am J Pathol 174:1663–1674. doi: 10.2353/ajpath.2009.080687

21. Sharma R, Tiwari R, Zhou Y, O'Sullivan J, Maekawa H, Borkowski GS, Bukaric H, An SY, Thorp EB, Chandel NS, Quaggin SE, Kapitsinou PP (2026) Endothelial prolyl hydroxylase 3 mitigates maladaptive inflammation to promote post-ischemic kidney repair. Kidney Int S0085-2538(26)00089-X. doi: 10.1016/j.kint.2026.01.017

22. Sun M, Zhang X (2022) Current methodologies in protein ubiquitination characterization: from ubiquitinated protein to ubiquitin chain architecture. Cell Biosci 12:126. doi: 10.1186/s13578-022-00870-y

23. Yin A, Fu W, Elengickal A, Kim J, Liu Y, Bigot A, Mamchaoui K, Call JA, Yin H (2024) Chronic hypoxia impairs skeletal muscle repair via HIF-2α stabilization. J Cachexia Sarcopenia Muscle 15:631–645. doi: 10.1002/jcsm.13436

24. Zhang R, Wu Y, Zhao M, Liu C, Zhou L, Shen S, Liao S, Yang K, Li Q, Wan H (2009) Role of HIF-1α in the regulation ACE and ACE2 expression in hypoxic human pulmonary artery smooth muscle cells. Am J Physiol-Lung Cell Mol Physiol 297:L631–L640. doi: 10.1152/ajplung.90415.2008

25. Zhang W, Li Y, Wang J-G (2024) Hypertension Induced by Hypoxia-Inducible Factor Prolyl Hydroxylase Inhibitors in Treating Anemia in Patients With Chronic Kidney Disease: A Mini-Review. J Clin Hypertens 26:1375–1383. doi: 10.1111/jch.14924

Dear Dr Broeker,

Re: JP-RP-2026-290695R1 "**The Endocrine Product of Renal (Preglomerular) Contractile Pericytes Depends on the Prolyl-4-Hydroxylases 2 and 3**" by Bettina Katharina Mariana Firmke, Lena Marie Süß, Anna-Lena Forst, Armin Kurtz, and Katharina Anna-Elisabeth Broeker

We are pleased to tell you that your paper has been accepted for publication in The Journal of Physiology.

The last Word (or similar) version of the manuscript provided will be used by the Production Editor to prepare your proof. When this is ready you will receive an email containing a link to Wiley's Online Proofing System. The proof should be thoroughly checked and corrected as promptly as possible.

Authors should note that it is too late at this point to offer corrections prior to proofreading. Major corrections at proof stage, such as changes to figures, will be referred to the Editors for approval before they can be incorporated. Only minor changes, such as to style and consistency, should be made at proof stage. Changes that need to be made after proof stage will usually require a formal correction notice.

All queries at proof stage should be sent to: TJP@wiley.com.

Following copyediting and typesetting the accepted version will be published online.

Yours sincerely,

Kim Barrett
Senior Editor
The Journal of Physiology

IMPORTANT POINTS TO NOTE FOLLOWING ACCEPTANCE OF YOUR PAPER:

• IMPORTANT NOTICE ABOUT OPEN ACCESS: To assist authors whose funding agencies mandate immediate public access to published research findings, The Journal of Physiology allows authors to pay an Open Access (OA) fee to have their papers made freely available immediately on publication.

The Corresponding Author will receive an email from Wiley with details on how to register or log in to Wiley Authors where you will be able to place an order.

• You can check if your funder or institution has a Wiley Open Access Account here:
https://authors.wiley.com/author-resources/Journal-Authors/open-access/author-compliance-tool.html

• TRANSPARENT PEER REVIEW POLICY: To improve the transparency of its peer review process, The Journal of Physiology publishes online as supporting information the peer review history of all articles accepted for publication. Readers will have access to decision letters, including Editors' comments and referee reports, for each version of the manuscript as well as any author responses to peer review comments. Referees can decide whether or not they wish to be named on the peer review history document.

• You can help your research get the attention it deserves! Check out Wiley's free Promotion Guide for best-practice recommendations for promoting your work at: www.wileyauthors.com/eeo/guide. You can learn more about Wiley Editing Services which offers professional video, design, and writing services to create shareable video abstracts, infographics, conference posters, lay summaries, and research news stories for your research at: www.wileyauthors.com/eeo/promotion.

• If you would like to receive our 'Research Roundup', a monthly newsletter highlighting the cutting-edge research published in The Physiological Society's family of journals (The Journal of Physiology, Experimental Physiology, Physiological Reports, The Journal of Nutritional Physiology and The Journal of Precision Medicine: Health and Disease), please click this link, fill in your name and email address and select 'Research Roundup':
https://www.physoc.org/journals-and-media/membernews

EDITOR COMMENTS

Reviewing Editor:

Thank you for your careful and thorough revision. The reviewers agree that their comments have been comprehensively addressed and that the manuscript has benefited substantially from the changes made. The study is now clear, well supported and presents a strong and convince body of work. We appreciate your thoughtful engagement with the review process.

REFEREE COMMENTS

Referee #1:

All of my concerns have been sufficiently addressed. I have no further comments at this time.

Referee #2:

The authors were highly responsive to the reviewers' comments. The manuscript has been substantially improved. All my concerns have been addressed. This is a very strong study. Congratulations to the authors.
